# Supplementary material for: In vivo systematic detection of the outcomes of CRISPR-Cas9-mediated DNA repair in skeletal muscle stem cells
Source: Mol Ther. 2026 Apr 20;34(7):3924–48. doi: 10.1016/j.ymthe.2026.04.032 (PMC13330020; doi:10.1016/j.ymthe.2026.04.032)
Supplement: Supplementary file 1 [file mmc1.pdf]

YMTHE, Volume *34*

Supplemental Information

In vivo systematic detection of the outcomes of CRISPR-Cas9-mediated DNA repair in skeletal muscle stem cells

Liangqiang He, Yang Fu, Ziliu Wang, Qin Zhou, Hao Sun, and Huating Wang

Supplemental Material

List of Supplemental Tables

Table S1. List of single, dual sgRNA, IDM-Seq, and Endo-LI donor sites.

Table S2. SIQ run information.

Table S3. SIQ outcomes for single-sgRNA sites.

Table S4. Jaccard similarity scores between target sites.

Table S5. SIQ outcomes for dual-sgRNA sites.

Table S6. SIQ outcomes for endo-LI donor samples.

Table S7. IDM-Seq indel profiles and statistics.

Table S8. SIQ outcomes for AAV dose groups.

Table S9. SIQ outcomes for *in vivo* perturbation.

Table S10. Sequences of oligos used in this study.

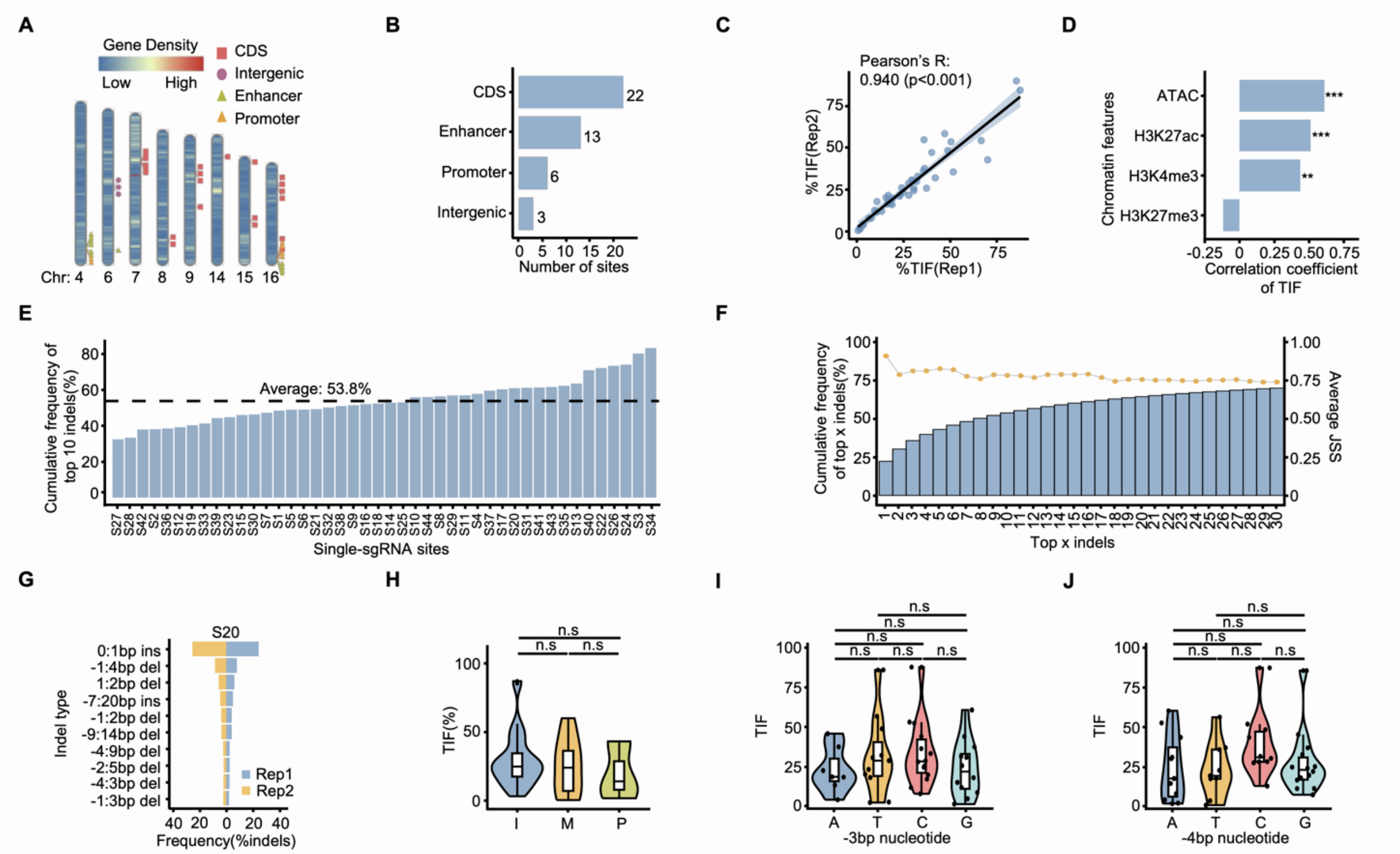

Figure S1. ***In vivo*** **mapping of CRISPR/Cas9 induced indel profiles in MuSCs using single-sgRNAs.** (**A**) Distribution of the 44 single-sgRNA targets across chromosomes. Genomic annotation of each target is shown. (**B**) The number of target sites with the indicated genomic annotations. (**C**) Correlation of TIF between two biological replicates across all 44 targets. (**D**) Spearman's correlation between TIF and indicated chromatin features. (**E**) The cumulative indel frequency of the top ten most frequent indels for each target. The average frequency across all sites is shown by the dash line. (**F**) The cumulative indel frequency of the top 1 to 30 most frequent indels across 44 targets, along with the corresponding average JSS of paired replicates across all targets. (**G**) Frequency of the top ten indel types in each replicate of S20. The y-axis displays the indel identity (start coordinate relative to the breakpoint): (indel size and type). (**H**) The TIF in I, M and P groups, respectively. (**I-J**) The TIF at sites with A, T, C, G at -3 bp (**I**) or -4 bp (**J**) upstream of the PAM motif. Statistical significance for (**H**) - (**J**) was calculated using a t-test. ** $p < 0.01$, ***$p < 0.001$ and ns, no significance.

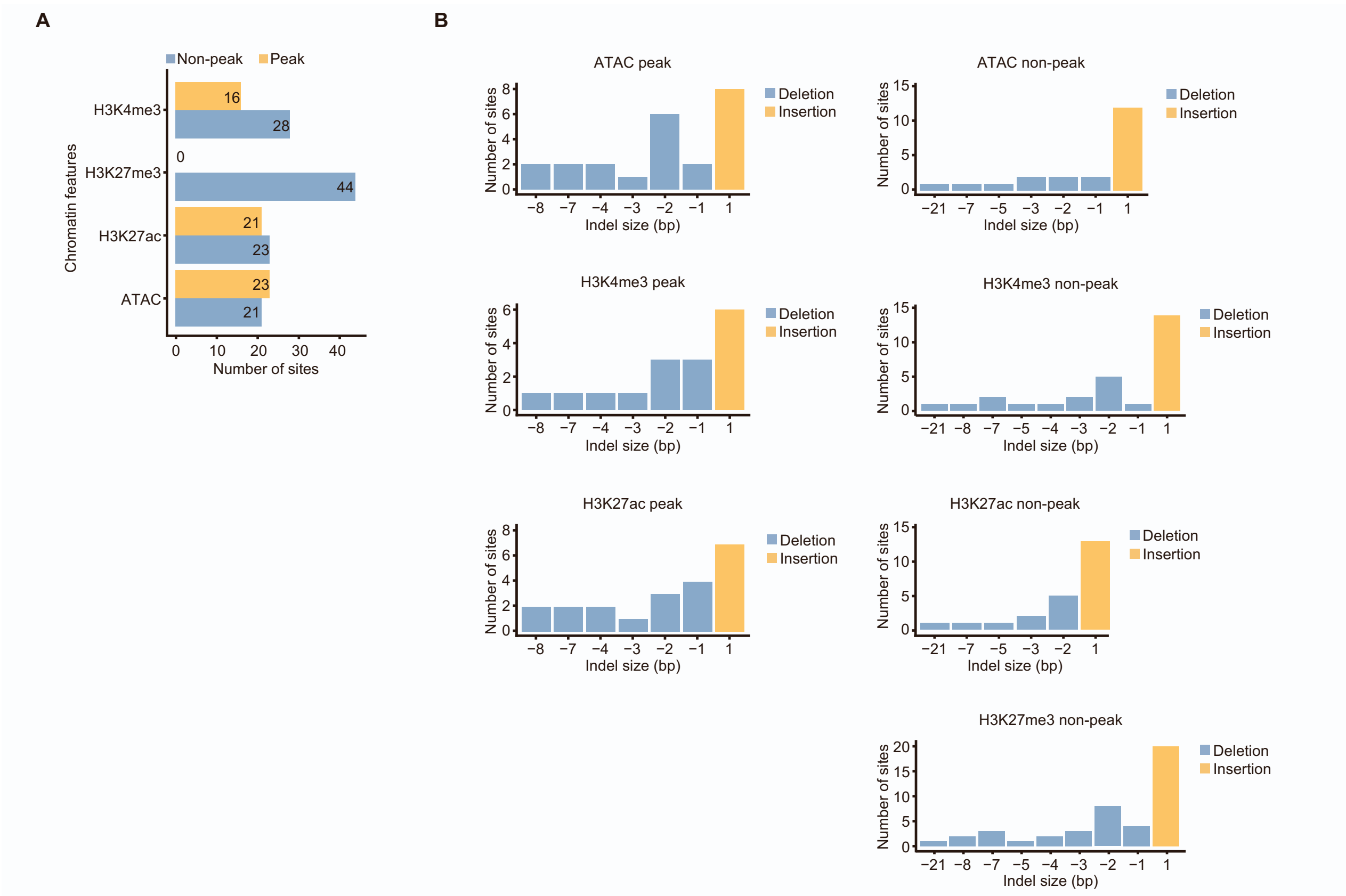

Figure S2. Impact of chromatin states on editing precision. (**A**) Numbers of sites located in peak or non-peak regions of each indicated chromatin feature. (**B**) The number of sites with the indicated type of top 1 indels in peak or non-peak regions of each indicated chromatin feature.

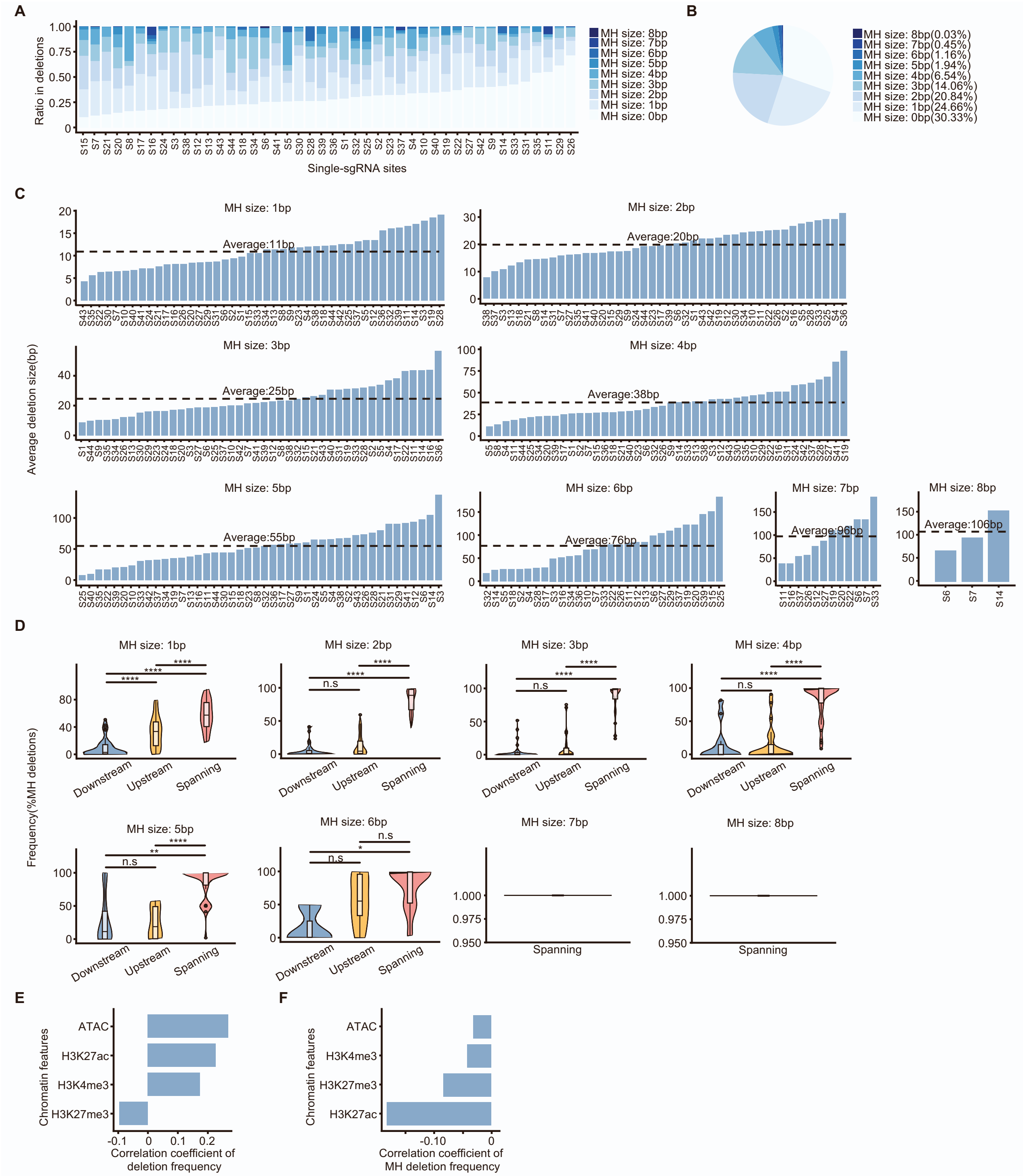

Figure S3. Extensive use of microhomology in CRISPR/Cas9 induced deletions in MuSCs *in vivo*. (A) Ratio of deletions with MH of varying sizes at each site. (**B**) Pie chart showing the average frequency of deletions with MH of varying sizes across all sites. (**C**) The average deletion sizes categorized by varying

MH lengths. (**D**) Violin plots illustrating the frequency of deletions with varying MH sizes categorized by direction. Statistical significance was assessed using a t-test. *$p < 0.05$, ** $p < 0.01$, ****$p < 0.0001$ and ns, no significance. (**E**-**F**) Spearman's correlation between deletion frequency (**E**) or MH frequency in deletions (**F**) and indicated chromatin features.

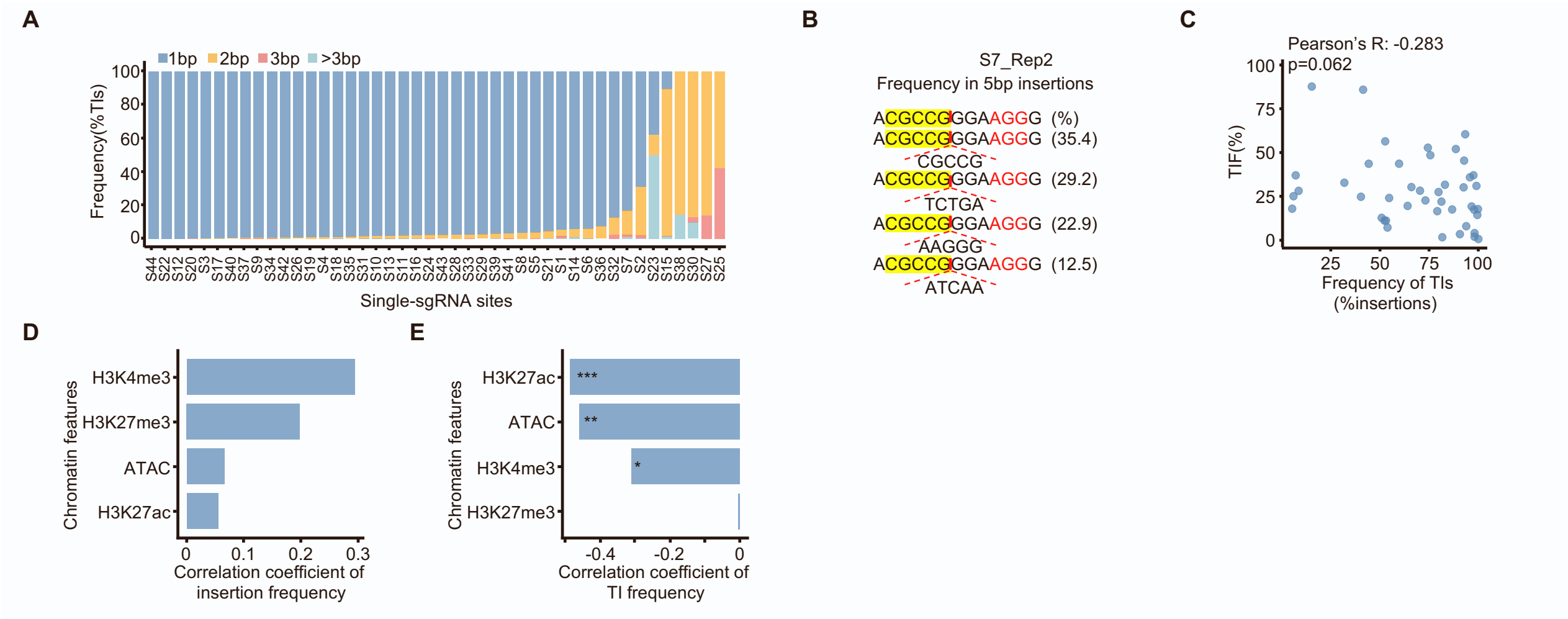

Figure S4. CRISPR/Cas9-mediated short insertions in MuSCs *in vivo* are templated and predictable. (**A**) Frequency of TIs with varying sizes in all TIs for each target site. (**B**) An example of > 3 bp TIs on S7. The nucleotides at -8 bp to -4 bp upstream of the PAM motif are highlighted in yellow. The frequency is presented as percentage in 5 bp insertions. (**C**) Correlation between TIF and TI frequency in all insertions. (**D**-**E**) Spearman's correlation between insertion frequency (**D**) or TI frequency (**E**) and indicated chromatin features. *p < 0.05, ** p < 0.01, ***p < 0.001.

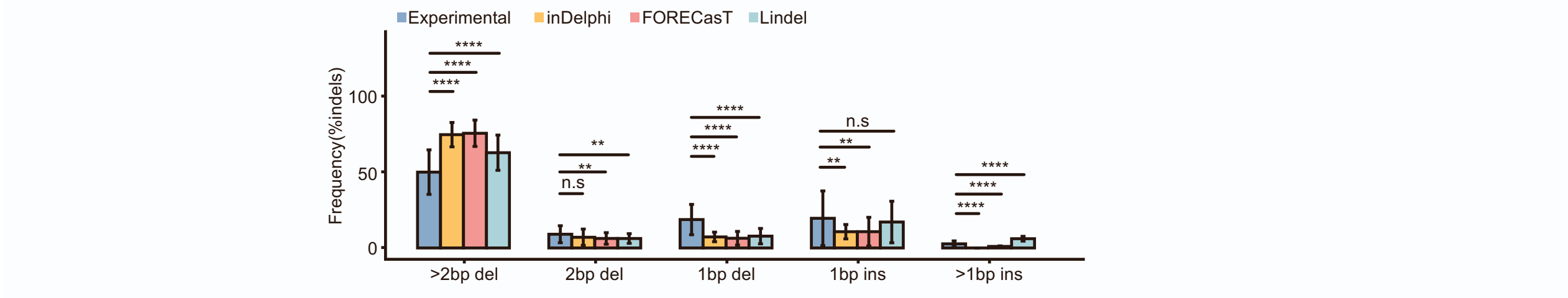

Figure S5. Limited predicting power of the machine learning models *in vivo*. Comparison between the frequency in total indels predicted by inDelphi, FORECasT, and Lindel and the experimentally measured for > 2 bp deletions, 2 bp deletions, 1 bp deletions, 1 bp insertions and > 1 bp insertions across the 44 sites, respectively. Statistical significance was calculated using a t-test. ** $p < 0.01$, ****$p < 0.0001$ and ns, no significance.

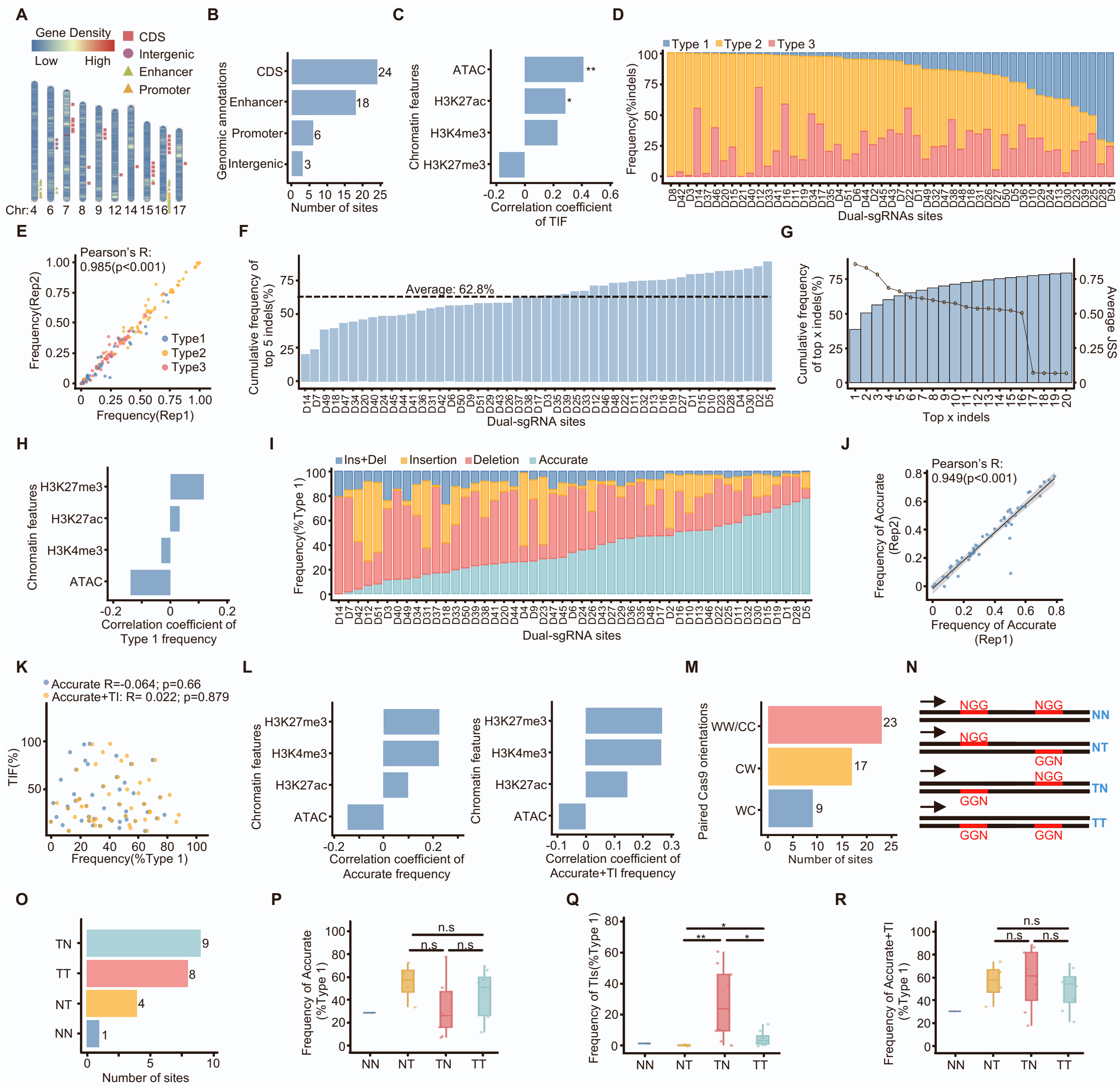

Figure S6. *In vivo* mapping of CRISPR/Cas9 induced indel profiles in MuSCs using dual-sgRNAs. (**A**) Distribution of the 51 dual-sgRNA targets across chromosomes. Genomic annotation of each target is shown. (**B**) The number of target sites with the indicated genomic annotations. (**C**) Spearman's correlation between TIF and indicated chromatin features. (**D**) Frequency of Type 1, Type2 and Type 3 indels for each target. (**E**) Correlation of Type 1, Type 2 and Type 3 indel frequency between two biological replicates across all 51 targets. (**F**) The cumulative indel frequency of the top five most frequent indels for each site. The average cumulative indel frequency across all sites is shown. (**G**) The cumulative indel frequency of

the top 1 to 20 most frequent indels across 51 targets, along with the corresponding average JSS of paired replicates across all targets. (**H**) Spearman's correlation between Type 1 frequency and indicated chromatin features. (**I**) Frequency of different subtypes of indel within Type 1 group for each target. 49 targets with over 100 Type 1 events were included for subtype analysis. (**J**) Pearson's correlation of the frequency of accurate ligation (Accurate) in Type 1 events between two biological replicates across targets. (**K**) Correlation of TIF with the frequency of Accurate (blue) or Accurate + TI (orange) in Type 1 indels. (**L**) Spearman's correlation between the frequency of Accurate (Left) or Accurate + TI (Right) in Type 1 indels and indicated chromatin features. (**M**) Numbers of target sites categorized by WW/CC, CW and WC PAM orientation. (**N**) Schematic showing four Cas9 PAM orientations for dual sgRNAs related to the transcription direction. TT, TN, NT, and NN orientations are determined by the recognition of Cas9 to the PAM motif on either the transcribed (T) or non-transcribed strand (N). Black arrows indicate the direction of transcription. (**O**) Numbers of target sites categorized by TT, TN, NT, and NN orientation. (**P**-**R**) Boxplot illustrating the frequency of Accurate (**P**), TIs (**Q**), Accurate + TI (**R**) in total Type 1 on targets with TT, TN, NT, and NN orientation. Statistical significance was calculated using a t-test for categories with $n \geq 3$ in (**P**) - (**R**). *$p < 0.05$, ** $p < 0.01$ and ns, no significance.

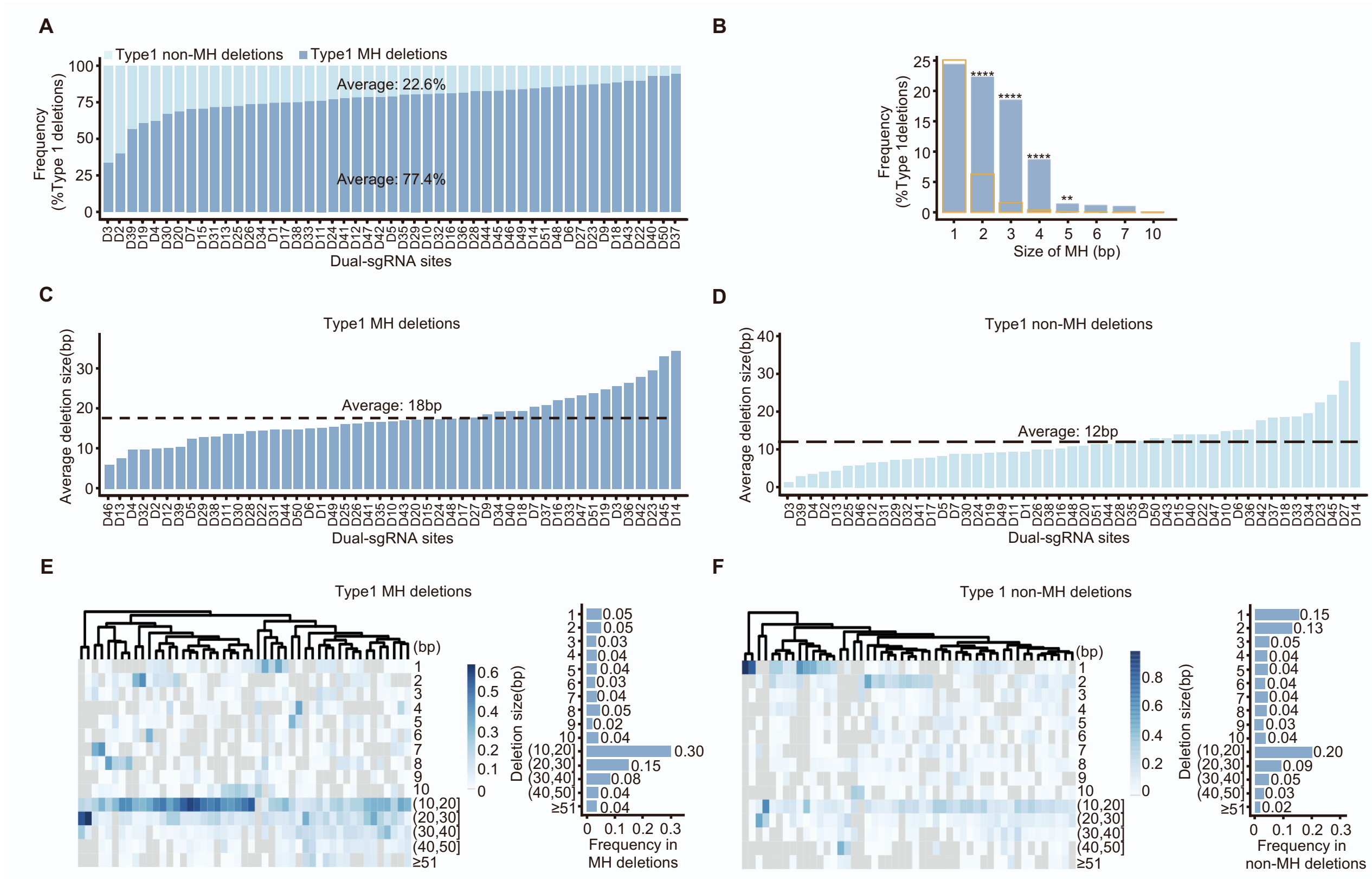

Figure S7. Extensive use of microhomology in dual sgRNAs induced deletion in MuSCs *in vivo*. (**A**) Frequency of MH and non-MH mediated deletions in Type 1 deletions for each dual-sgRNA target. The average frequency of MH and non-MH mediated deletions across all sites is shown. (**B**) Frequency of deletions with MH of varying sizes in Type 1 deletions. The orange bar represents the expected frequency. (**C**-**D**) Average deletion size for MH (**C**) and non-MH (**D**) mediated deletions for each site. The average deletion size for MH and non-MH mediated deletions across all dual-sgRNA sites is shown by dash line. (**E**) Left: Heatmaps illustrating the frequency of MH mediated deletions categorized by size across dual-sgRNA targets. The targets are clustered using the "complete" hierarchical clustering. Right: The average frequency across all targets for each category with varying deletion size in MH mediated deletions. (**F**) The above analysis was conducted in non-MH mediated deletions. Statistical significance was calculated using a one-tailed t-test for (**B**). ** $p < 0.01$, ****$p < 0.0001$.

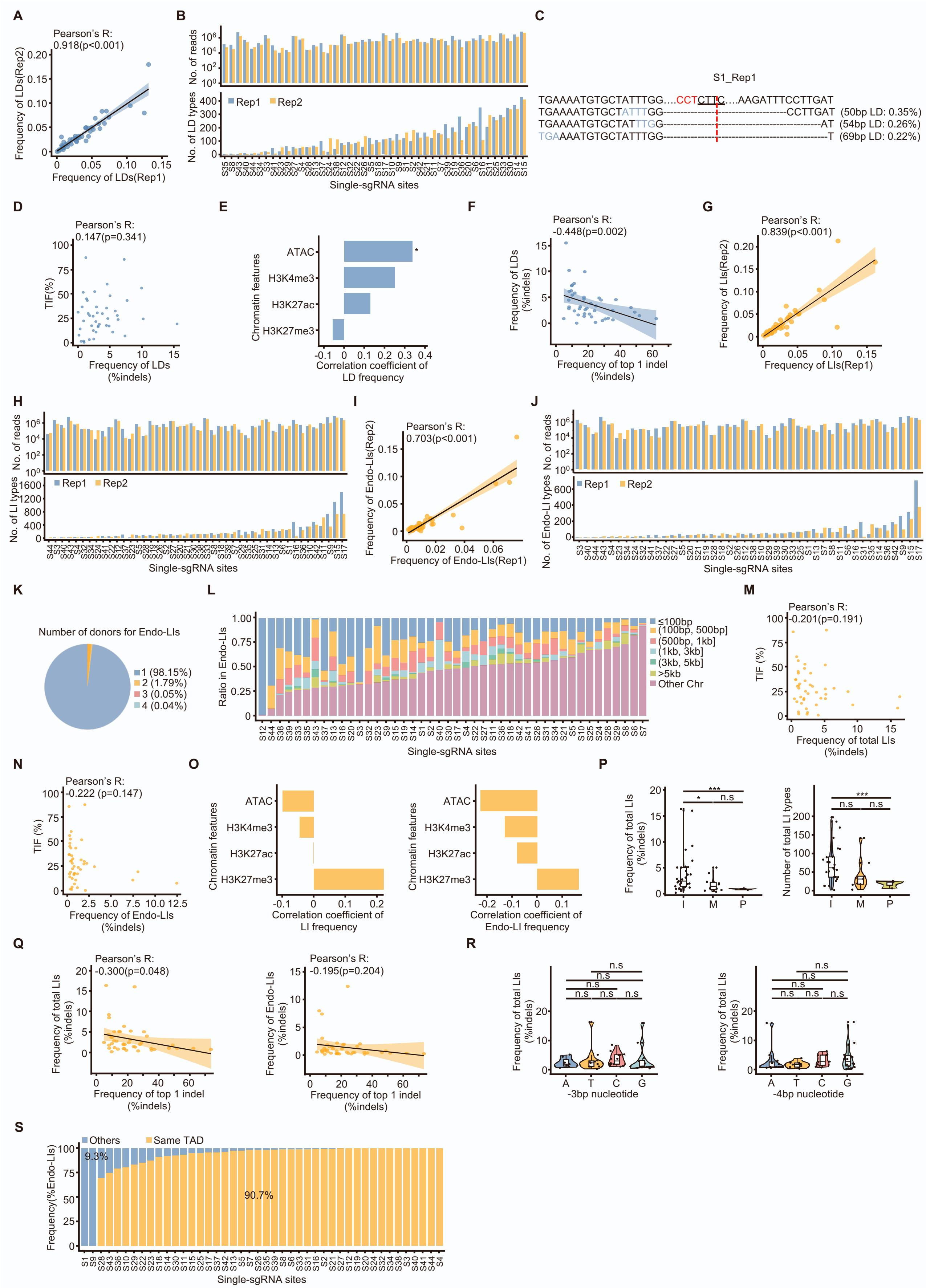
A
Pearson's R:
0.918(p<0.001)
Frequency of LDs(Rep2)
Frequency of LDs(Rep1)
B
No. of reads
No. of LD types
Rep1
Rep2
Single-sgRNA sites
C
S1_Rep1
TGAAAATGTGCTATTTGG....CCTCTTC....AAGATTTCCTTGAT
TGAAAATGTGCTATTTGG------------------------------CCTTGAT (50bp LD: 0.35%)
TGAAAATGTGCTATTTGG------------------------------AT (54bp LD: 0.26%)
TGAAAATGTGCTATTTGG------------------------------T (69bp LD: 0.22%)
D
Pearson's R:
0.147(p=0.341)
TIF(%)
Frequency of LDs
(%indels)
E
ATAC
H3K4me3
H3K27ac
H3K27me3
Chromatin features
Correlation coefficient of
LD frequency
F
Pearson's R:
-0.448(p=0.002)
Frequency of LDs
(%indels)
Frequency of top 1 indel
(%indels)
G
Pearson's R:
0.839(p<0.001)
Frequency of LIs(Rep2)
Frequency of LIs(Rep1)
H
No. of reads
No. of LI types
Rep1
Rep2
Single-sgRNA sites
I
Pearson's R:
0.703(p<0.001)
Frequency of Endo-LIs(Rep2)
Frequency of Endo-LIs(Rep1)
J
No. of reads
No. of Endo-LI types
Rep1
Rep2
Single-sgRNA sites
K
Number of donors for Endo-LIs
1 (98.15%)
2 (1.79%)
3 (0.05%)
4 (0.04%)
L
Ratio in Endo-LIs
≤100bp
(100bp, 500bp]
(500bp, 1kb]
(1kb, 3kb]
(3kb, 5kb]
>5kb
Other Chr
Single-sgRNA sites
M
Pearson's R:
-0.201(p=0.191)
TIF (%)
Frequency of total LIs
(%indels)
N
Pearson's R:
-0.222 (p=0.147)
TIF (%)
Frequency of Endo-LIs
(%indels)
O
ATAC
H3K4me3
H3K27ac
H3K27me3
Chromatin features
Correlation coefficient of
LI frequency
Correlation coefficient of
Endo-LI frequency
P
Frequency of total LIs
(%indels)
Number of total LI types
I
M
P
n.s
Q
Pearson's R:
-0.300(p=0.048)
Frequency of total LIs
(%indels)
Frequency of top 1 indel
(%indels)
Pearson's R:
-0.195(p=0.204)
Frequency of Endo-LIs
(%indels)
R
Frequency of total LIs
(%indels)
-3bp nucleotide
-4bp nucleotide
n.s
S
Others
Same TAD
9.3%
90.7%
Frequency(%Endo-LIs)
Single-sgRNA sites

Figure S8. Analysis of CRISPR/Cas9 induced large on-target genomic modifications in MuSCs *in vivo*. (**A**) Pearson's correlation of LD frequency in total indels between two biological replicates across single-sgRNA targets. (**B**) Number of LD types defined by their start sites and the deleted sequences in each biological replicate across all targets (Lower) and number of sequencing reads for each sample (Upper). (**C**) An example showing the frequency of LDs with MH on S1. The MH tracks are marked as blue. (**D**) Pearson's correlation between TIF and LD frequency. (**E**) Spearman's correlation between LD frequency in total indels and indicated chromatin features. (**F**) Pearson's correlation between LD frequency in all indels and editing precision determined by the frequency of top 1 indel. (**G**) Pearson's correlation of LI frequency in total indels between two biological replicates across single-sgRNA targets. (**H**) Number of LI types defined by their start sites and the inserted sequences in each biological replicate across all targets (Lower) and number of sequencing reads for each sample (Upper). (**I**) Pearson's correlation of Endo-LI frequency between two biological replicates across single-sgRNA targets. (**J**) Number of Endo-LI types defined by their start sites and the inserted sequences in each biological replicate across all targets (Lower) and number of sequencing reads for each sample (Upper). (**K**) Pie charting showing the number distribution of donors for Endo-LIs. (**L**) Distance distribution between insertion donors for Endo-LIs and associated DSBs for each site. (**M**-**N**) Pearson's correlation of TIF with total LI (**M**) and Endo-LI (**N**) frequency. (**O**) Spearman's correlation between the frequency of total LIs (Left) or Endo-LIs (Right) and indicated chromatin features. (**P**) Violin plot illustrating the frequency of total LIs (Left) and number of total LI types (Right) in I, M, P groups. (**Q**) Pearson's correlation between the frequency of total LI (Left) or Endo-LI (Right) and editing precision determined by the frequency of top 1 indel. (**R**) Frequency of total LIs at sites with A, T, C, G at -3 bp (Left) of -4 bp (Right) upstream of the PAM motif. (**S**) Frequency of donor sequences originating from the DSB-containing TADs or other loci on the same chromosome for each site. Statistical significance was calculated using a t-test for (**P**) and (**R**). $*p < 0.05$, $***p < 0.001$ and ns, no significance.

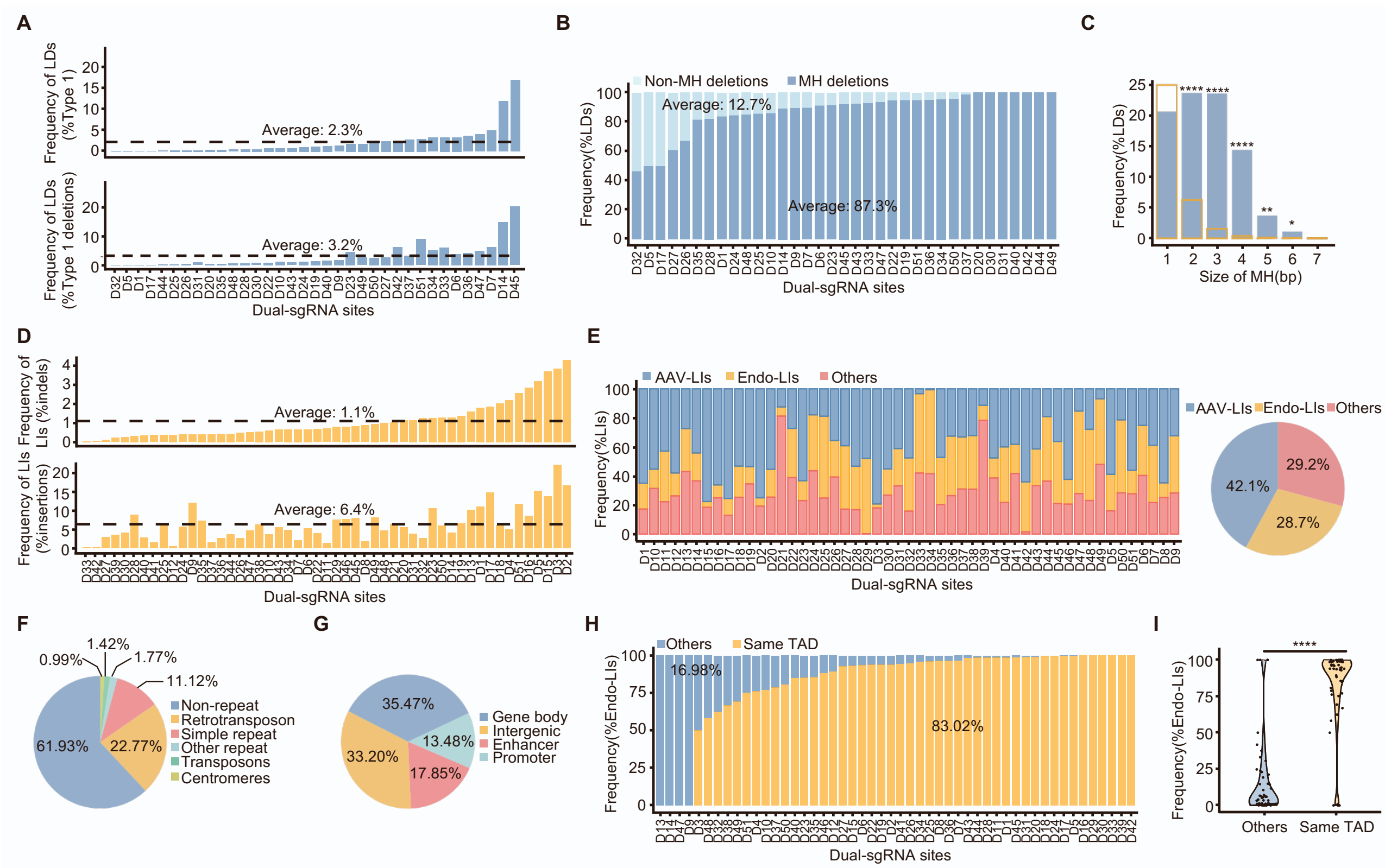

Figure S9. Analysis of CRISPR/Cas9 induced large on-target genomic modifications induced by dual-sgRNAs in MuSCs *in vivo*. (**A**) Frequency of ≥ 50 bp LDs for each dual-sgRNA site, calculated as percentage in total Type 1 events (Upper) and total Type 1 deletions (Lower). The average frequency of LDs across all sites is shown by dash line. (**B**) Frequency of MH and non-MH mediated LDs for each dual-sgRNA target. The average frequency of MH and non-MH mediated LDs across all sites is shown. (**C**) Frequency of LDs with MH of varying sizes. The orange bar represents the expected frequency for each MH size. (**D**) Frequency of ≥ 20 bp LIs for each dual-sgRNA site, calculated as percentage in total indels (Upper) and total insertions (Lower). The average frequency of LIs is shown by dash line. (**E**) Left: Frequency of different types of LIs for each dual-sgRNA target. Right: Pie chart showing the average frequency of each LI type across all sites. (**F**) Pie chart showing the frequency of donor sequence categorized by types of repetitive elements for Endo-LIs. (**G**) Pie chart showing the frequency of donor sequence categorized by genomic annotations for Endo-LIs. (**H**) Frequency of donor sequences originating from the DSB-containing TADs or other loci on the same chromosome for each dual-sgRNA site. (**I**) Violin

plot showing significantly higher frequency of donor sequences originating from DSB-containing TADs compared to other loci on the same chromosome. A one-tail t-test was used for (**C**) and a paired t-test was used for (**I**). *p < 0.05, ** p < 0.01, ****p < 0.0001.

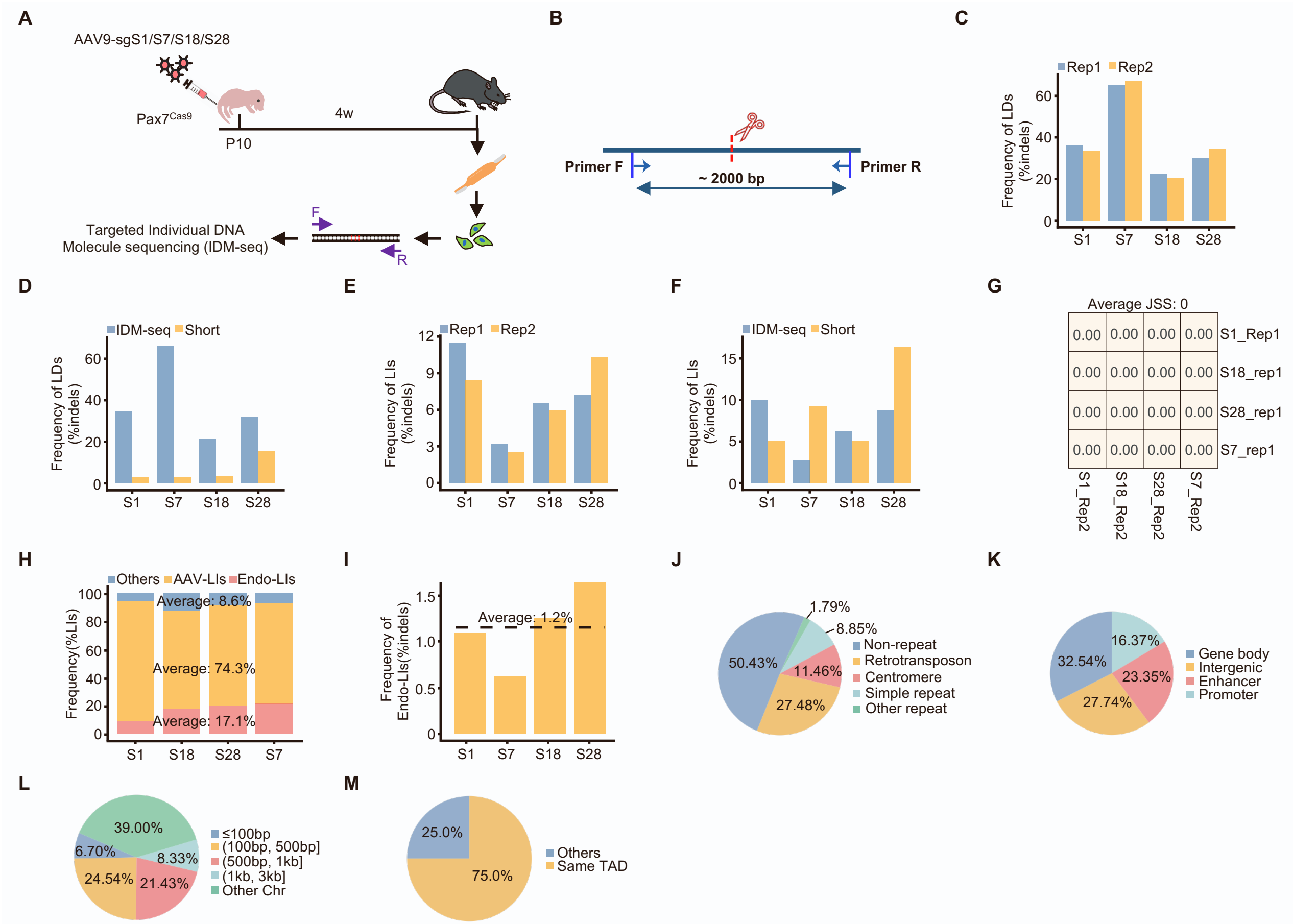

Figure S10. Detection of large on-target modifications by long-read sequencing. (**A**) Schematic showing experimental design for detecting large on-target modifications by IDM-seq at S1, S7, S18 and S28. 5×10^{11} vg of AAV9-dual sgRNA virus targeting each site was IM injected into Pax7^{Cas9} mouse at P10, followed by MuSC isolation four weeks after injection. Sequences encompassing each site were amplified and subjected to IDM-seq. (**B**) Schematic showing the amplification range for IDM-seq. An approximately 2,000 bp region flanking the Cas9 cut site was amplified for each site. (**C**) Frequency of ≥ 50 bp LDs for each site, calculated as percentage in total indels. (**D**) Comparison of the average LD frequency detected by IDM-seq vs. short-read NGS. (**E**) Frequency of ≥ 20 bp LIs for each site, calculated as percentage in total indels. (**F**) Comparison of the average LI frequency detected by IDM-seq vs. short-read NGS. (**G**) JSSs for LIs detected by IDM-seq. Each square shows the JSS between all LIs of corresponding targets. The average JSS of paired replicates across all targets is shown. (**H**) Frequency of different types of LIs.

The average frequency of each type across all sites is shown. (**I**) Frequency of ≥ 20 bp Endo-LIs for each site, calculated as percentage in total indels. The average frequency is shown by dash line. (**J**) Pie chart showing the frequency of donor sequence categorized by types of repetitive elements for Endo-LIs. (**K**) Pie chart showing the frequency of donor sequence categorized by genomic annotations for Endo-LIs. (**L**) Pie chart showing distribution of distances between insertion donors for Endo-LIs and associated DSBs. (**M**) Pie chart showing the frequency of donor sequences originating from the DSB-containing TADs or other loci on the same chromosome.

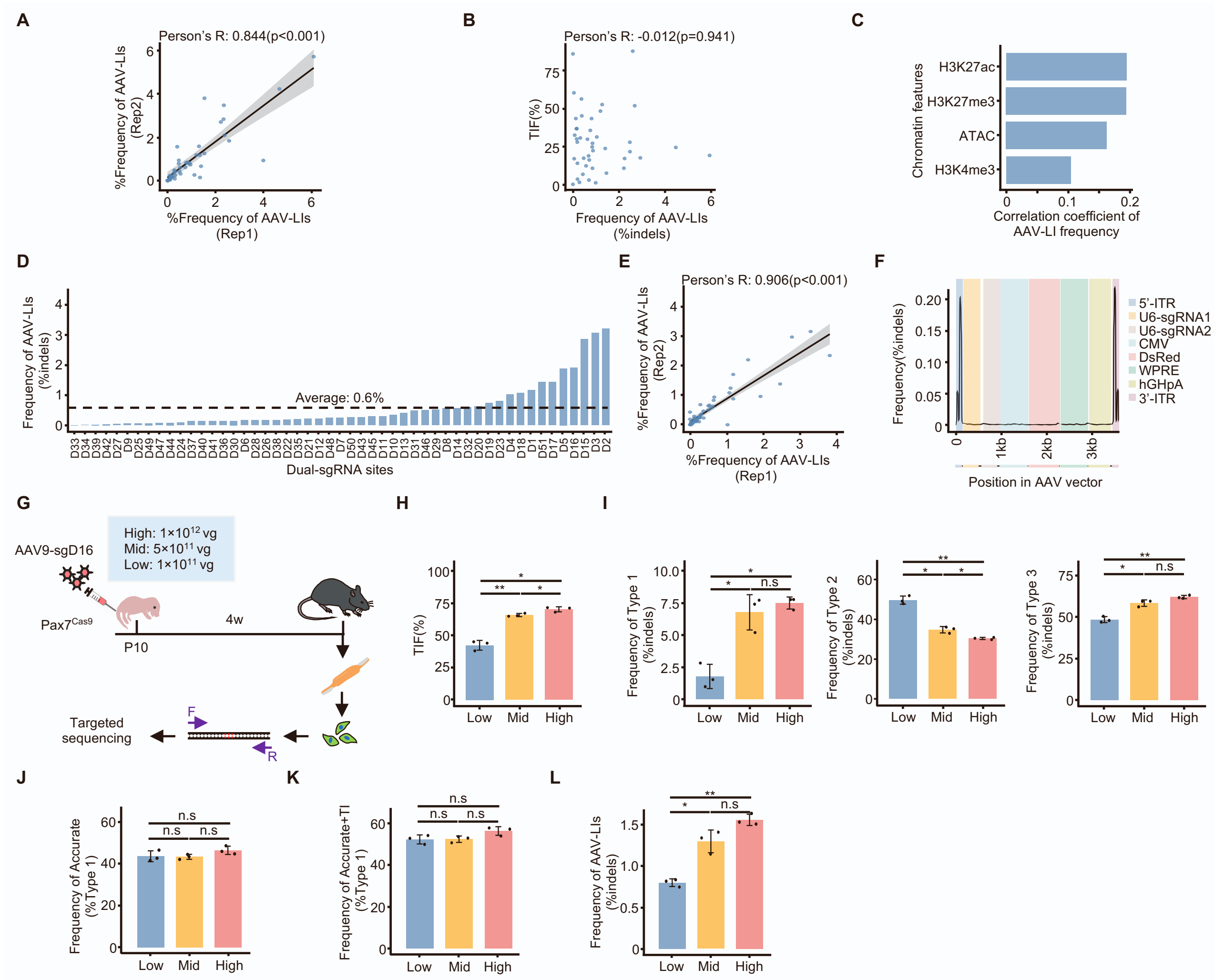

Figure S11. AAV integration is a general outcome of CRISPR/Cas9-induced editing in MuSCs *in vivo*. (**A**) Pearson's correlation of AAV-LI frequency in total indels between two biological replicates across single-sgRNA targets. (**B**) Pearson's correlation of TIF with AAV-LI frequency. (**C**) Spearman's correlation between AAV-LI frequency and the indicated chromatin features. (**D**) Frequency of AAV-LIs in dual-sgRNA group, calculated as percentage in total indels. The average frequency of AAV-LIs across all sites is shown by the dash line. (**E**) Pearson's correlation of AAV-LI frequency between two biological replicates across dual-sgRNA targets. (**F**) Average insertion frequency of indicated AAV-dual sgRNA vector regions. (**G**) Schematic showing experimental design for assessing the effect of AAV dosage on AAV-LI at the D16 site. High (1×10^{12} vg), Mid (5×10^{11} vg) or Low (1×10^{11} vg) dose of AAV9-dual sgRNA virus targeting the

D16 site were IM injected into Pax7^{Cas9} mouse at P10 (n=3 mice), followed by MuSC isolation four weeks after injection. Sequences encompassing the D16 site were amplified and subjected to unbiased deep-sequencing and analysis. (**H**-**L**) Barplot illustrating TIF (**H**), frequency of Type 1 indels (**I**, Left), Type 2 indels (**I**, Middle), Type 3 indels (**I**, Right), Accurate events (**J**), Accurate + TI events (**K**) and AAV-LIs (**L**) for the three dosage groups (n=3 mice in each group). Statistical significance was calculated using a t-test for (**H**) - (**L**). *$p < 0.05$, **$p < 0.01$ and ns, no significance.

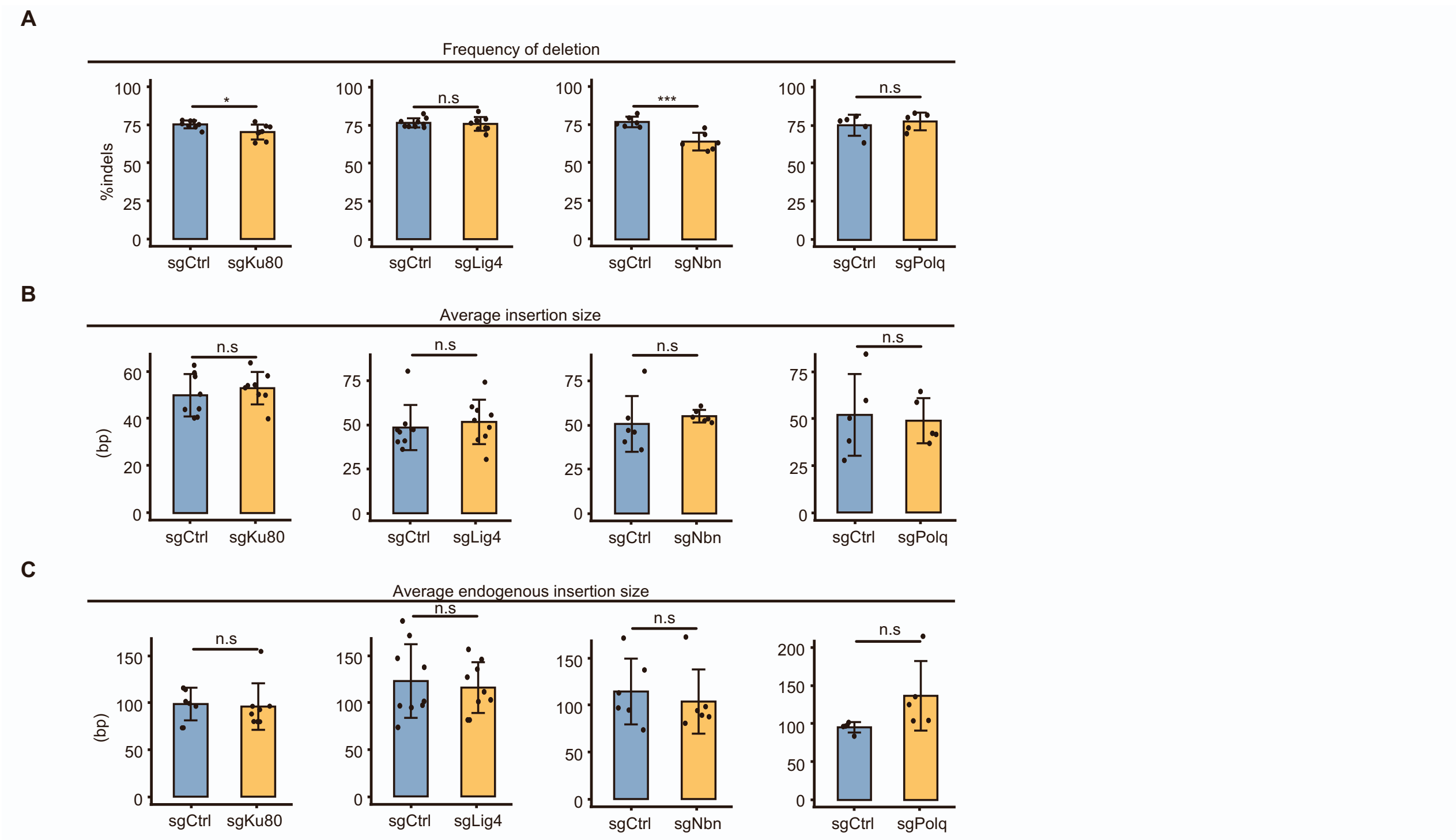

Figure S12. *In vivo* perturbation of NHEJ/MMEJ pathways can modulate CRISPR/Cas9 editing in MuSCs. (**A**-**C**) Frequency of deletions (**A**), average insertion size (**B**) and average endogenous insertion size (**C**) after *in vivo* depletion of KU80, LIG4, NBN or POLQ is shown. n=5 to 9 mice in each group. Statistical significance was calculated using a paired t-test for (**A**) - (**C**). *p < 0.05, ***p < 0.001 and ns, no significance.

Intended	Small deletion
	Small insertion
Un-intended	Large deletion
	Large insertion(Vector intergration; Endo-LI)
	Genomic rearrangement
	Chromosome truncation
	Copy-neutral loss of heterozygosity
	Whole chromosome loss
	Chromothripsis

Figure S13. Summary of main repair outcomes mediated by CRISPR/Cas9.

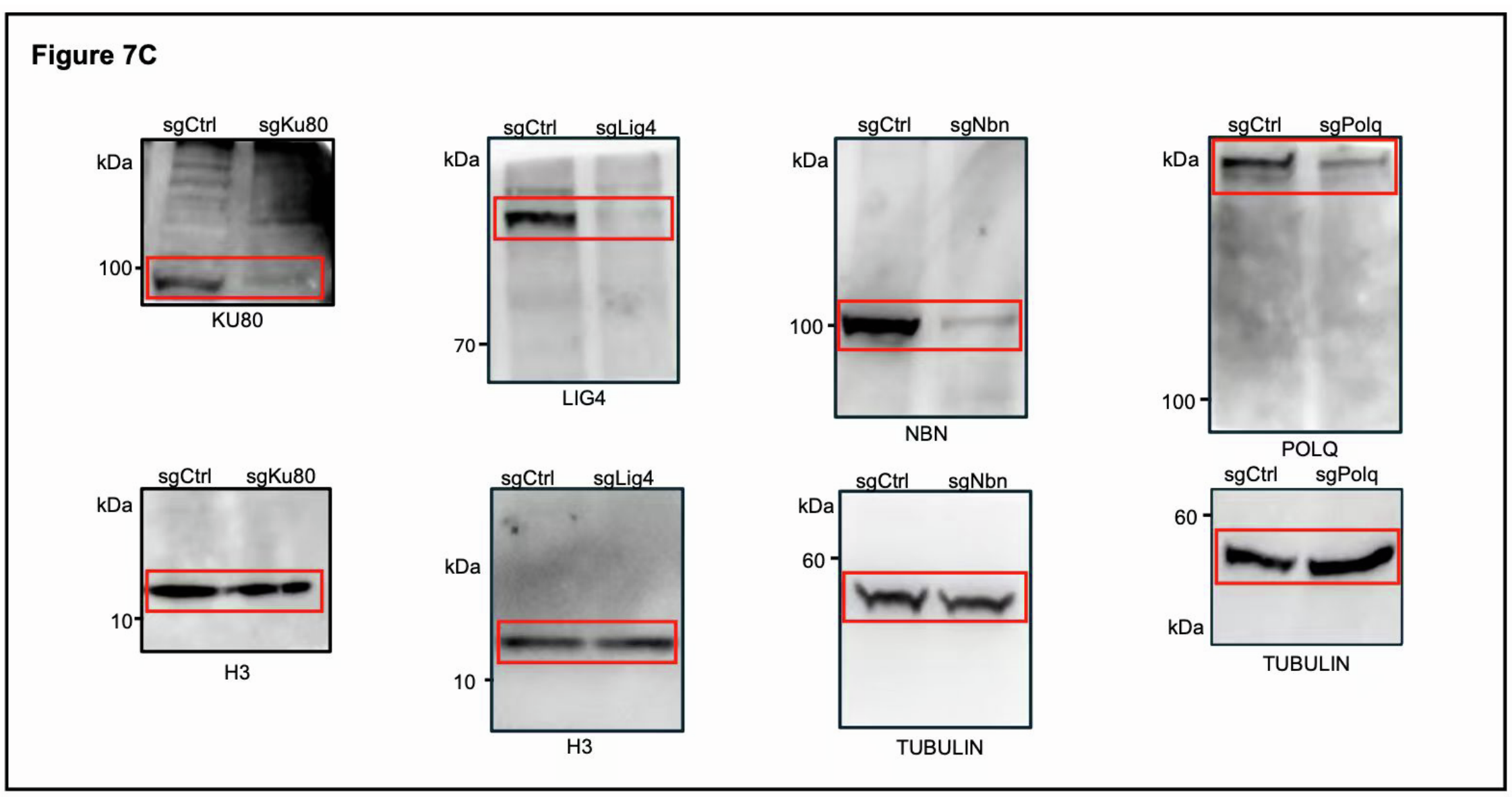

Figure S14. Uncropped Western blot images.
