# Supplementary material for: In vivo systematic detection of the outcomes of CRISPR-Cas9-mediated DNA repair in skeletal muscle stem cells
Source: Mol Ther. 2026 Apr 20;34(7):3924–48. doi: 10.1016/j.ymthe.2026.04.032 (PMC13330020; doi:10.1016/j.ymthe.2026.04.032)
Supplement: Supplementary file 2 [file mmc12.pdf]

Molecular Therapy
Original Article

In vivo systematic detection of the outcomes of CRISPR-Cas9-mediated DNA repair in skeletal muscle stem cells

Liangqiang He,[1,2,4] Yang Fu,[1,4] Ziliu Wang,[1] Qin Zhou,[1] Hao Sun,[3] and Huating Wang[1,2]

[1]Department of Orthopaedics and Traumatology, Li Ka Shing Institute of Health Sciences, The Chinese University of Hong Kong, Hong Kong SAR, China; [2]InnoHK Center for Neuromusculoskeletal Restorative Medicine, Hong Kong Science Park, Hong Kong SAR, China; [3]Department of Bioinformatics, Warshel Institute for Computational Biology, CUHK-Shenzhen-Boao International Hospital Joint Laboratory for Precision Medicine Diagnostics, School of Medicine, The Chinese University of Hong Kong (Shenzhen), Shenzhen, Guangdong, China

Clustered regularly interspaced short palindromic repeats (CRISPR)-CRSIPR-associated protein 9 (Cas9) has revolutionized genome editing with broad therapeutic applications, yet its repair patterns *in vivo* remain poorly understood. Here, we systematically profile CRISPR-Cas9 editing outcomes at 95 loci using our established CRISPR-Cas9/adeno-associated virus (AAV)9-single guide RNA (sgRNA) system in skeletal muscle stem cells (MuSCs). Through comprehensive characterization of the repair outcomes, our findings demonstrate that the general rules governing CRISPR-Cas9-mediated editing *in vivo* largely align with those observed *in vitro*. In addition to the anticipated small editing insertions or deletions (indels), such as microhomology-mediated end joining (MMEJ)-mediated deletions and non-homologous end joining (NHEJ)-mediated templated insertions, we uncover a prevalent occurrence of large on-target modifications, including large deletions (LDs) characterized by microhomology (MH) and large insertions (LIs). Notably, the LIs comprise not only exogenous AAV vector integrations but also endogenous genomic DNA fragments (Endo-LIs). Endo-LIs preferentially originate from active genomic regions, with their integration shaped by 3D chromatin architecture. By disrupting key components of the NHEJ and MMEJ repair pathways *in vivo*, we identify their distinct roles in regulating the large on-target modifications. Together, our work systematically profiles CRISPR-Cas9 repair outcomes *in vivo* and offers valuable guidance for improving the safety of CRISPR-Cas9-based gene therapies.

INTRODUCTION

Clustered regularly interspaced short palindromic repeats (CRISPR)-CRSIPR-associated protein 9 (Cas9) represents a groundbreaking genome editing technology that has revolutionized molecular biology and the development of potential therapies for human genetic disorders.[1,2] It initiates targeted DNA modifications by the recognition of a target sequence through the help of a CRISPR RNA (crRNA) and a *trans*-activating crRNA (tracrRNA), which can be integrated into a single guide RNA (sgRNA) to mimic their function. The sgRNA binds to the Cas9 protein, forming a ribonucleoprotein complex to locate its target next to a protospacer adjacent motif (PAM), typically "NGG" in the case of *Streptococcus pyogenes* Cas9 (SpCas9). Upon PAM recognition, the sgRNA hybridizes with the complementary DNA strand, forming an R-loop structure, where the non-target strand remains single stranded. The Cas9 protein harbors two nuclease domains, HNH and RuvC, which cleave the target and non-target DNA strands, respectively.[1,2] Typically, both cleavages occur 3 bp upstream of the PAM, leading to a blunt-ended double-strand break (DSB). However, accumulating evidence suggests that while the HNH domain cleaves precisely, the RuvC domain exhibits flexibility, cutting at positions −3 bp, −4 bp, −5 bp, or even further upstream, leading to the formation of both blunt and 5′ staggered ends.[3,4]

In eukaryotic cells, CRISPR-Cas9-generated DSBs are primarily repaired through three competing pathways: homology-directed repair (HDR), non-homologous end joining (NHEJ), and microhomology-mediated end joining (MMEJ).[5] HDR is a high-fidelity repair mechanism that uses a homologous DNA template to precisely repair the break. However, HDR is primarily active during the S and G2 phases of the cell cycle, with significantly diminished activity in non-dividing or terminally differentiated cells. In contrast, NHEJ is the predominant repair pathway used in most cells and is characterized by its rapid kinetics. This pathway acts without needing a homologous template and typically introduces small insertions or deletions (indels) at the break site, normally within 2 bp.[6]

Received 10 November 2025; accepted 14 April 2026;
https://doi.org/10.1016/j.ymthe.2026.04.032.

[4]These authors contributed equally

Correspondence: Hao Sun, Department of Bioinformatics, Warshel Institute for Computational Biology, CUHK-Shenzhen-Boao International Hospital Joint Laboratory for Precision Medicine Diagnostics, School of Medicine, The Chinese University of Hong Kong (Shenzhen), Shenzhen, Guangdong, China.
E-mail: sunhao100@cuhk.edu.cn
Correspondence: Huating Wang, Department of Orthopaedics and Traumatology, Li Ka Shing Institute of Health Sciences, The Chinese University of Hong Kong, Hong Kong SAR, China.
E-mail: huating.wang@cuhk.edu.hk

 © 2026 The Author(s).
Published by Elsevier Inc. on behalf of The American Society of Gene and Cell Therapy.
This is an open access article under the CC BY license (http://creativecommons.org/licenses/by/4.0/).

Although NHEJ is generally considered error-prone, emerging evidence from cultured cells *in vitro* or massively synthetic target sequences suggests that it can mediate precise and predictable repair of Cas9-induced DSBs.[3,4,7,8] Once a DSB forms, NHEJ directly ligates the two broken DNA ends without modifications, leading to repeated cycles of Cas9 cleavage until an indel occurs to prevent further sgRNA target recognition.[5,9] When staggered ends with 5′ overhangs arise due to the flexible cutting of the RuvC domain, the NHEJ pathway fills in the gaps and joins the two ends together, resulting in insertions that are templated by the sequence upstream of the cut site. In single-sgRNA-induced editing, the most consistently predictable outcome is the insertion of a single nucleotide identical to the nucleotide located −4 bp upstream of the PAM.[3,8] Accordingly, in dual-sgRNA-induced fragment excision, the two DSBs are preferentially ligated accurately by NHEJ, resulting in precise deletions or templated insertions (TIs).[7] Beyond HDR and NHEJ, alternative repair pathways, such as MMEJ or polymerase theta-mediated end joining (TMEJ), as recently described,[10,11] can also contribute to DSB repair. Like NHEJ, MMEJ does not require a template to repair the damaged DNA but utilizes short microhomologies (MHs) present near the broken DNA ends to repair the DSBs.[5,6] MMEJ is normally responsible for generating small deletions ($\geq$3 bp) within 10 bp,[6,12] but a growing number of studies suggest that MMEJ can also lead to the occurrence of large deletions (LDs; >50 bp).[10,13,14]

The general indel patterns induced by CRISPR-Cas9 have been extensively studied *in vitro*.[8,15–17] High-throughput profiling of repair outcomes indicates that the indels resulting from end-joining repair of these DSBs are predictable and significantly influenced by the sequence context surrounding the targeted site.[8,15–17] Based on these findings, several sophisticated machine learning models have been developed to predict the predominant indels of Cas9-induced DSBs. Besides, the local chromatin structure also impacts Cas9-generated indel patterns.[18] Notably, NHEJ-mediated repair of Cas9-induced DSBs is more efficient in euchromatin, whereas MMEJ is preferentially active in specific heterochromatic regions. Mechanistically, NHEJ is typically initiated by the KU70–KU80 heterodimer following DSB formation, which binds to broken DNA ends to prevent resection.[5,6] This complex subsequently recruits DNA-dependent protein kinase catalytic subunit (DNA-PKcs) and DNA ligase IV (LIG4) to facilitate end ligation.[5,6] In contrast, HDR and MMEJ require end resection by the MRE11-RAD50-NBN (MRN) complex and its cofactor C-terminal binding protein (CtBP) interacting protein (CtIP) to expose MHs. Strategies to regulate the DSB repair pathway choice by modulating the activities of these key components have been developed to facilitate more efficient and precise editing. In CRISPR-Cas9-related *ex vivo* gene therapy, a common strategy is to enhance HDR levels by simultaneously suppressing MMEJ and NHEJ activity using specific inhibitors targeting key factors of these pathways.[5,6]

Other than the predictable small indels, recent studies have identified undesirable editing byproducts: complex on-target large gene modifications, which raise critical concerns about the safety in clinical applications of CRISPR-Cas9.[13,19,20] Gross genomic modifications can occur at the target site, including LDs, large insertions (LIs)—especially the integration of exogenous vector DNA—and even chromosomal structural rearrangements and chromosomal loss.[21–23] These unintended alterations arise from the cellular repair triggered by Cas9-induced DSBs and thus cannot be avoided by using high-fidelity Cas9 variants. Overall, the current understanding of on-target large genomic modifications remains limited. It is shown that MHs are enriched at LD breakpoint junctions, and LD frequency increases in NHEJ-deficient cells but declines in cells with impaired MMEJ activity, implicating MMEJ in LD formation.[13,14] On the other hand, LIs frequently arise from the integration of exogenous vector fragments into DSBs within the host genome. The mechanisms underlying vector integration remain unclear; however, abolishing either the NHEJ or MMEJ pathway can inhibit random integration, indicating that both pathways may contribute to CRISPR-Cas9-induced large exogenous vector insertions.[24,25] In addition, recent studies have revealed that LIs at CRISPR-Cas9-induced DSBs can also originate from endogenous genomic sequences.[23,26] Comprehensive characterization of these endogenous LIs (Endo-LIs) is currently lacking, and their mechanisms of formation remain poorly understood.

CRISPR-based gene editing has made remarkable progress in treating diverse human genetic disorders.[1,27] In particular, *in vivo* genome editing, which delivers CRISPR machinery directly to affected tissues via localized or systemic administration, offers the promise of targeting a broader spectrum of diseases. Advanced delivery platforms leverage both non-viral and viral-based vectors, with adeno-associated virus (AAV) emerging as the leading choice for *in vivo* gene therapy applications due to its tissue-specific tropism, sustained transgene expression, and favorable safety profile.[28] While *in vitro* CRISPR-Cas9-induced DNA repair has been extensively studied, a systematic characterization of its performance *in vivo* remains lacking. Given that the DNA repair process may be modulated by cellular context and tissue-specific factors[5]—which are still poorly understood—it remains uncertain whether the repair patterns observed *in vitro* fully recapitulate those *in vivo*. Moreover, the presence of large on-target genomic modifications needs further investigation. Although the incidence of such large chromosomal aberrations could be rare *in vivo*, even a small number of edited cells harboring deleterious mutations could clonally expand and lead to serious abnormalities. For instance, while AAV predominantly remains episomal, the AAV vector integrated into DSBs within the host genome can be detected after *in vivo* administration.[9,29,30] Therefore, systematically analyzing these unwanted byproducts and elucidating their formation mechanisms to minimize their occurrence *in vivo* is crucial for improving the safety of CRISPR-Cas9-based therapies.

In our previous work, we established a CRISPR-Cas9/AAV9-sgRNA platform for *in vivo* editing in skeletal muscle stem cells (MuSCs) characterized by paired-box gene 7 (PAX7) expression.[31,32] This platform presented high efficiency in introducing mutagenesis at

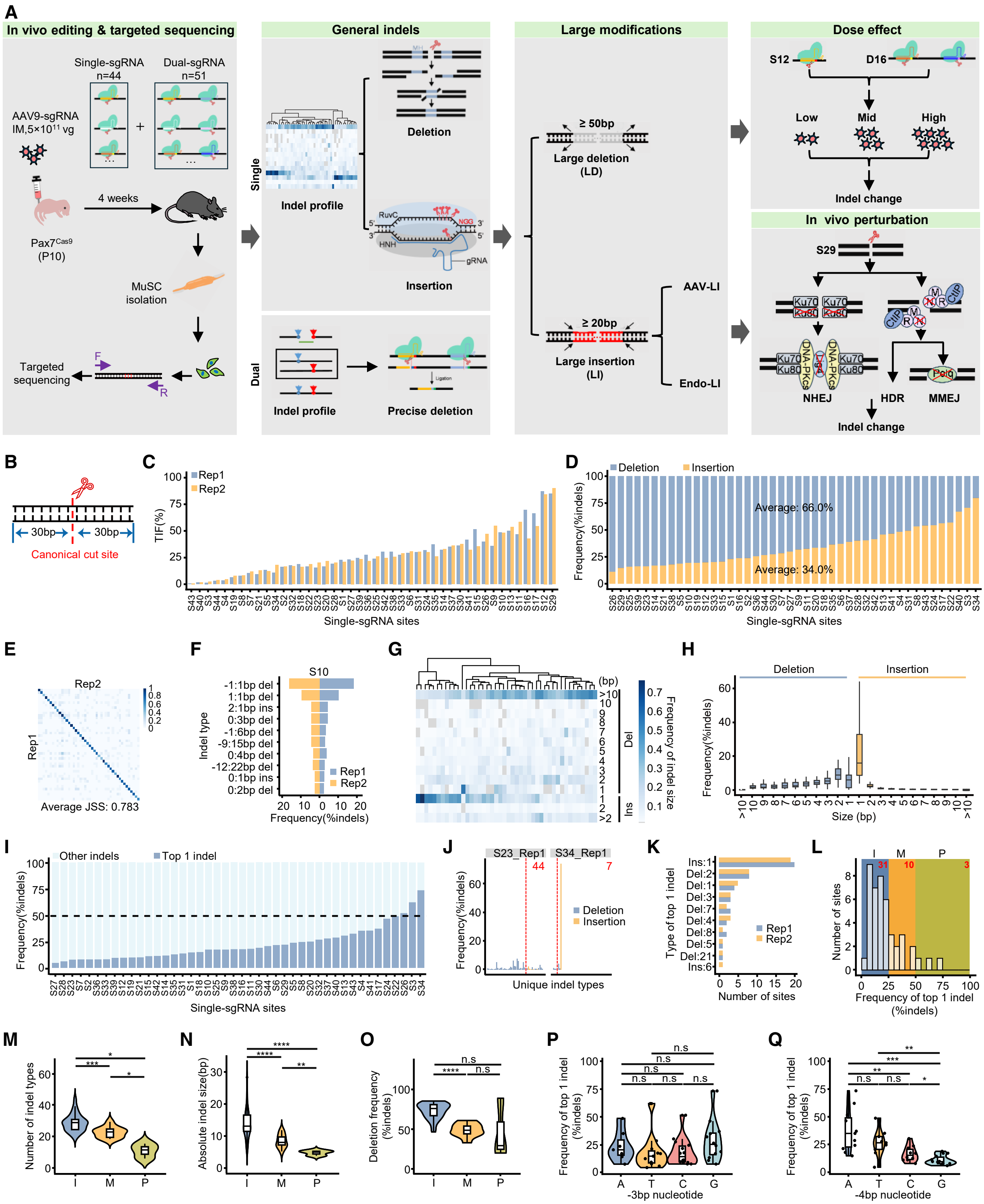

(legend on next page)

multiple genomic loci in MuSCs, establishing it as a robust tool for studying CRISPR-Cas9-mediated editing outcomes *in vivo*.[31,33–35] Here, leveraging this system, we systematically edited 95 loci in MuSCs *in vivo* using single or dual sgRNAs. Using targeted deep sequencing (Deep-seq), we performed in-depth characterization of CRISPR-Cas9-induced repair profiles to decipher the indel patterns and assess large on-target modifications. Our results indicate that CRISPR-Cas9-induced indel patterns *in vivo* largely align with those observed *in vitro*. Moreover, we uncovered prevalent large on-target modifications *in vivo*, including LDs and LIs. Notably, the LIs comprise not only exogenous AAV vector integrations but also endogenous genomic DNA fragments. These endogenous fragments are preferentially derived from active genomic regions, and the insertion is influenced by the 3D chromatin architecture. Finally, by knocking out key factors involved in NHEJ and MMEJ *in vivo*, we revealed their differential regulatory effects on these large on-target modifications. Collectively, our work systematically uncovers CRISPR-Cas9 repair outcomes *in vivo* and offers valuable guidance for enhancing the safety of CRISPR-Cas9-based *in vivo* gene therapies.

RESULTS

In vivo mapping of CRISPR-Cas9-induced indel profiles in MuSCs using single sgRNAs

To uncover the patterns of indel profiles induced by CRISPR-Cas9 *in vivo*, we took advantage of our previously developed CRISPR-Cas9/AAV9-gRNA editing system to conduct a multi-loci profiling of the repair outcomes in MuSCs *in vivo*[31,32] (Figure 1A). In brief, a muscle-specific Cas9 knockin mouse line was generated by crossing Pax7^{Cre} with Cre-dependent Cas9 knockin mice (referred to as Pax7^{Cas9} mice).[31–35] Skeletal muscle-tropic AAV9 virus containing locus-specific single or dual sgRNA was intramuscularly (i.m.) injected into the Pax7^{Cas9} mouse at postnatal day 10 (P10), when postnatal myogenesis was active and cells were in a proliferative myoblast state. Quiescent MuSCs emerged approximately 2 weeks after birth, carrying the editing, and were isolated 4 weeks after the AAV injection for editing outcome detection. This strategy has been successfully applied in our prior studies to introduce mutagenesis at multiple target loci in MuSCs *in vivo*.[31–35] 44 single sgRNAs were designed to target distinct genomic sites, designated numerically from S1 to S44 (Table S1). These sites covered coding sequences (CDSs) and promoter regions of seven transcription factors, including *Myc*, *Bcl6*, *Myod1*, *Rora*, *Runx1*, *Pknox2*, and *Ctcf*. Additionally, 13 were designed to target potential enhancer regions. To further increase the diversity and complexity of the targeted sites, we also designed three sgRNAs targeting non-regulatory intergenic regions (Figures S1A and S1B; Table S1). Two biological replicates were included for each target site. Sequences encompassing each sgRNA targeting site were amplified and subjected to unbiased next-generation sequencing (NGS) for precise characterization of editing efficiency and indel profiles (Figure 1A; Tables S2 and S3). To eliminate potential sequencing and amplification artifacts, corresponding control Deep-seq libraries were generated for each target site using genomic DNA from unedited MuSCs of Pax7^{Cas9} mice. Indels detected in these control samples were excluded from subsequent analysis (Tables S2 and S3). To ensure a consistent basis for monitoring the distribution of the indel profiles across amplicons of diverse sizes, only indels overlapping within a 60 bp window centered on the canonical cut site (3 bp upstream of the NGG PAM sequence) were analyzed (Figure 1B). We first examined the editing efficiency by calculating the total indel frequency (TIF) and found it varied across different loci (ranging from 0.66% to 90.1%, with an average of 28.4%) (Figure 1C). A strong similarity between the two replicates of the same locus was detected (Pearson's R: 0.940) (Figure S1C). As expected, the TIF generally correlated positively with active chromatin regions featured by assay for transposase-accessible chromatin using sequencing (ATAC-seq) and H3K4me3/ H3K27ac chromatin immunoprecipitation sequencing (ChIP-seq) signals but not repressive H3K27me3 ChIP-seq signals[6,18] (Figure S1D).

We next examined the indel profiles to uncover the general patterns of repair outcomes driven by single-sgRNA-directed Cas9 activity. The indels were quantified based on their mutation type (deletions or insertions). Of note, complex repair outcomes with simultaneous insertions and deletions were classified as insertions.[36] Interestingly, we found that deletions predominated in the overall indel landscape, accounting for an average of 66.0% of the total indels (Figure 1D). We then assessed if the editing is reproducible as observed

Figure 1. *In vivo* mapping of CRISPR-Cas9-induced indel profiles in MuSCs using single sgRNAs
(A) Overview of the experimental design/analysis for the study. (B) Schematic illustration of the 60 bp editing window centered on the canonical cut site indicated as a red dashed line. Only indels overlapping with this region were included in the analysis. (C) TIF for the 44 targets (each with 2 biological replicates). (D) Frequencies of deletions and insertions in total indels, averaged from two biological replicates per target. The average frequencies of deletions and insertions across all targets are shown. (E) Heatmap of Jaccard similarity scores (JSSs) of Rep1 and Rep2 for targets S1–S44. Each square indicates the JSS between the top ten most frequent indels of the corresponding target, while the diagonal highlights scores for identical targets. The average JSS of paired replicates across all targets is shown. (F) Frequency of the top ten indel types in each replicate of S10. The *y* axis displays the indel identity (start coordinate relative to the breakpoint): (indel size and type). (G) Heatmap illustrating the frequencies of indels categorized by size across 44 targets. The targets are clustered using the "complete" hierarchical clustering. (H) Boxplot illustrating the distribution of indels by size across targets. (I) Frequency of the top 1 and other indels in total indels for each target. (J) Indel profiles of an imprecise (S23, left) and a precise (S34, right) target. The canonical cut site is marked by a red dashed line. (K) The number of sites with the indicated type of top 1 indels in Rep1 (blue) and Rep2 (orange) across all target sites. (L) Distribution of the frequency of the top 1 indel across all target sites. Imprecise (I), middle (M), and precise (P) groups are indicated in blue, orange, and green, respectively. The inset numbers marked with red indicate the count of target sites in each group. (M–O) The number of indel types (M), absolute indel size (N), and frequency of deletions (O) in I, M, and P groups, respectively. (P and Q) Frequency of top 1 indel in total indels at sites with A, T, C, and G −3 (P) or −4 (Q) bp upstream of the PAM. Only indels with a frequency > 0.5% of the total indels were considered for (J) and (M). Statistical significance for (M) and (Q) was calculated using a *t* test. *$p < 0.05$, **$p < 0.01$, ***$p < 0.001$, ****$p < 0.0001$, and ns, no significance.

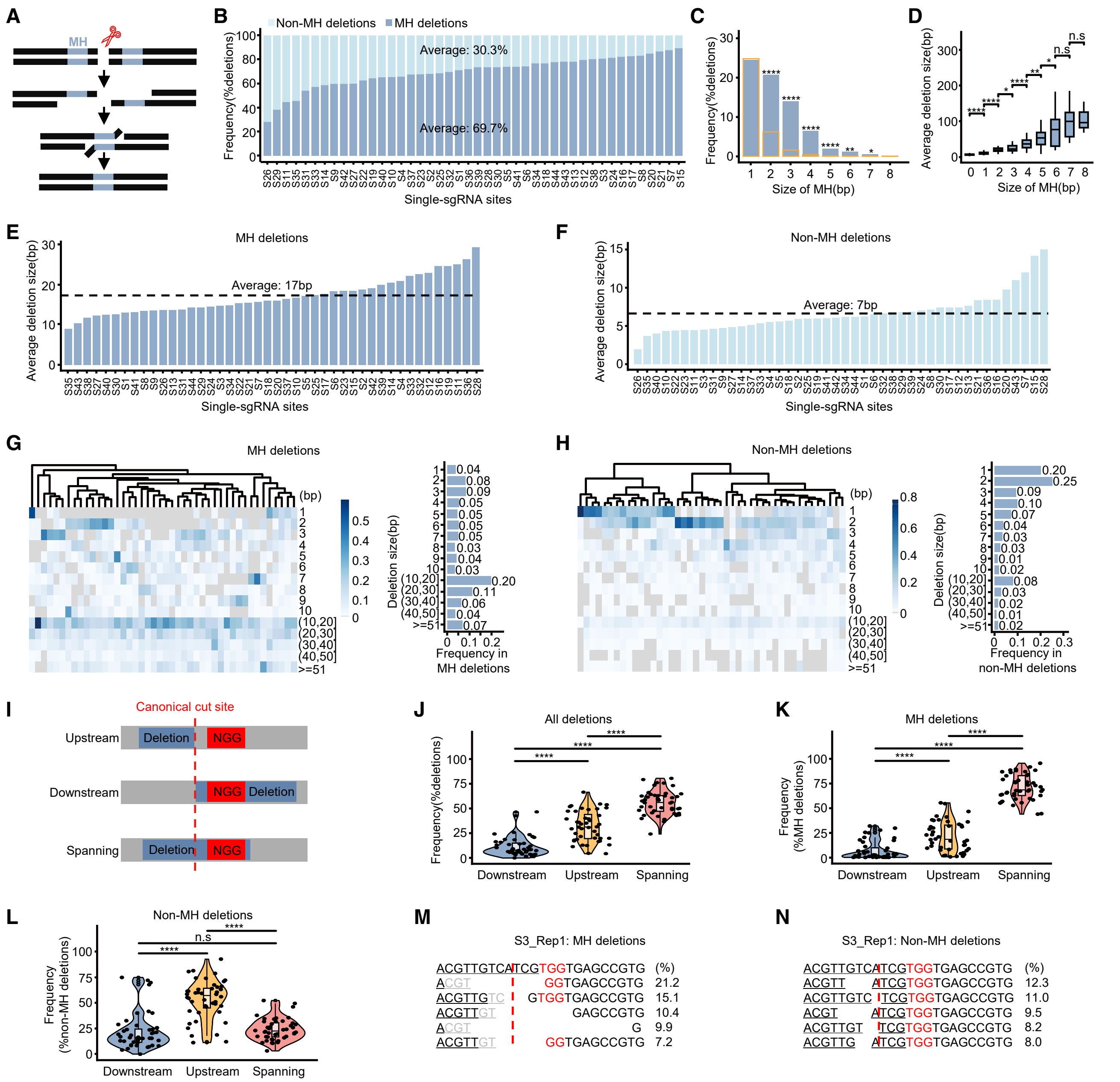

Figure 2. Extensive use of microhomology in CRISPR-Cas9-induced deletions in MuSCs *in vivo*

(A) Schematic illustrating MH-dependent deletions in CRISPR-Cas9-induced DSB repair. MH tracts (blue) flanking DSB facilitate the alignment of the broken ends. The unpaired overhang is cleaved by endonucleases, followed by DNA-polymerase-mediated gap filling and DNA-ligase-mediated breakpoint ligation. (B) Frequency of MH- and non-MH-mediated deletions in all deletions for each site. The average frequencies of MH- and non-MH-mediated deletions across all sites are shown. (C) Frequency of deletions with MH of varying sizes in all deletions. The orange bar represents the expected frequency for each MH size. (D) Average deletion size for deletions categorized by MH sizes. (E and F) Average deletion size for MH- (E) and non-MH- (F) mediated deletions for each site. MH- and non-MH-mediated average deletion sizes across all sites are shown by the dashed line. (G) Left: heatmaps illustrating the frequency of MH-mediated deletions (a proportion of total MH-mediated deletions) categorized by size across 44 targets. The targets are clustered using the "complete" hierarchical clustering. Right: the average frequency (indicated by inset numbers) across all targets for each category with varying deletion size in MH-mediated deletions. (H) The above analysis but in non-MH-mediated deletions. (I) Schematic diagram illustrating deletion directions. The NGG PAM is highlighted in a red background. (J–L) Violin plots illustrating the frequency of all deletions (J) and MH- (K), and non-MH- (L) mediated deletions categorized by

(legend continued on next page)

in vitro.[3,8,37,38] A Jaccard similarity score (JSS) was calculated for the top 10 most frequent indels (Table S4), which represented an average of 53.8% of all indels at each site (Figures S1E and S1F), and high similarity (0.783) was found between the two replicates, while samples from different sites showed minimal similarity (Figure 1E; Table S4). These results indicate that indel formation is not random and is target specific, consistent with findings from *in vitro* studies.[3,8,37,38] As exemplified at the S10 (Figure 1F) and S20 (Figure S1G) loci, the top 10 indel types—indicated by the type of the indel, the coordinate of the start site relative to the break point, the indel length, and the frequency— exhibited remarkable similarity; for example, the top 1 most frequent indel type in both replicates of S10 is a 1 bp deletion occurring 1 bp upstream of the breakpoint (−1:1bp del). Further unsupervised hierarchical clustering based on the frequencies of the identified indel classes revealed two major groups of sites with distinct indel signatures, including sites that preferentially exhibited 1 bp insertions and deletions >10 bp (Figure 1G). Overall, 1 bp insertion was the predominant mutational type, and frequencies declined with increasing size (Figure 1H); nevertheless, insertions and deletions larger than 10 bp were also noticed.

It has been reported that the level of editing precision can be assessed by the recurrence of specific indels at each target[37]; thus, we ranked all sites based on the prevalence of the top 1 most frequent indel. Our result showed that only three sites (3/44) exhibited a specific mutation type with a probability >50% (Figure 1I). Some sites displayed a remarkably diverse range of indel types with moderate frequency. For example, at the S23 site (Figure 1J, left), we detected up to 44 different types without there being a prominent one. In contrast, others, such as the S34 site (Figure 1J, right), showed a strong bias toward a single predominant indel, accompanied by only a few additional types, suggesting that the editing precision varied from site to site. Consistent with the highest frequency observed (Figure 1H), 1 bp insertions presented as the top 1 indel for 20 sites (Figure 1K). Nevertheless, a target favoring longer deletions up to 21 bp was also observed.

To delve into the factors that may be associated with editing precision, 44 target loci were divided into three groups[37]: imprecise (I; the frequency of top 1 indel $\leq$ 0.25), medium precision (M; 0.25 < the frequency of top 1 indel $\leq$ 0.5), and precise (P; 0.5 < the frequency of top 1 indel $\leq$ 1) (Figure 1L). Based on this classification, over half of the sites (31/44) were categorized into the I group. The TIF showed no significant difference among the three groups (Figure S1H). As expected, editing precision exhibited a negative correlation with the number of indel types, with the average number of indel types at imprecise sites (28.8) two times greater than that at precise sites (11.0) (Figure 1M). A similar pattern was observed with absolute indel size, where precise sites were more prone to producing short indels (Figure 1N). Moreover, group I exhibited a significantly higher portion of deletions than group M but not group P (Figure 1O). Previous studies have demonstrated that the nucleotide composition flanking the cut site—particularly at the position immediately downstream (−3 bp relative to PAM) and upstream (−4 bp)—can significantly influence DNA repair outcome[3,39]; thus, we examined whether nucleotide identity at these specific positions correlates with editing precision. At the −3 bp position, the presence of G was associated with an elevated but not significant mean frequency of the top 1 indels compared to other nucleotides (Figure 1P). Conversely, the −4 bp G was associated with a lower mean frequency of predominant indels (Figure 1Q). These nucleotide-dependent effects indicate that local DNA context surrounding the cleavage site may modulate editing precision *in vivo*. We also investigated whether the nucleotide composition flanking the cut site influences TIF and observed no such effect (Figures S1I and S1J).

Finally, we investigated the impact of chromatin states on editing precision, as recent studies have underscored the significance of chromatin context in determining the choice of DSB repair pathways *in vitro*.[6,18] The 44 sites were categorized into two groups based on their location in either peak or non-peak regions of various chromatin features, including H3K4me3, H3K27ac, H3K27me3, and chromatin openness determined by ATAC-seq (Figure S2A). By comparing the types of the top 1 indel, we found no obvious differences between the two categories, as a 1 bp insertion consistently predominated across all conditions (Figure S2B). This suggests that the chromatin environment has no apparent effect on *in vivo* editing precision. Altogether, these observations suggest that CRISPR-Cas9-induced indels in MuSCs *in vivo* display high reproducibility and are affected by local DNA context.

Extensive use of MH in CRISPR-Cas9-induced deletions in MuSCs *in vivo*

Considering that deletions constituted the majority of indels (Figure 1D), we subsequently investigated the underlying patterns of deletions. It is known that MH-mediated alignment of broken ends is commonly employed in CRISPR-Cas9-induced deletions[8,37] (Figure 2A). Therefore, we systematically evaluated the presence of MH at the breakpoint junctions. Our analysis indicated that 69.7% of the deletions were indeed characterized by the presence of MH (Figure 2B). The length of the MH varied from 1 to 8 bp, with the frequency of associated deletions gradually decreasing as MH size increased (Figures 2C and S3A). Overall, short MH tracks (1–4 bp) constituted most MH-mediated deletions (Figure S3B). Deletions utilizing 1 bp MH occurred most frequently, accounting for 24.66% of all deletions, and were detectable at all sites (Figures S3B and S3C). In contrast, longer MHs (MH > 4 bp) were observed at

direction. (M and N) An example of target site S3 illustrating the top five most frequent MH- (M) and non-MH- (N) mediated deletions. The frequency is presented as the percentage in all MH or non-MH deletions. The canonical cut site is indicated by a red dashed line, and the NGG PAM is highlighted in red. The guide sequence is underlined. The corresponding MH used for deletions in (M) is marked in gray. Statistical significance was calculated using a *t* test for (D) and (J)–(L) and a one-tailed *t* test (alternative = "greater") for (C). *$p < 0.05$, **$p < 0.01$, ****$p \leq 0.0001$, and ns, no significance.

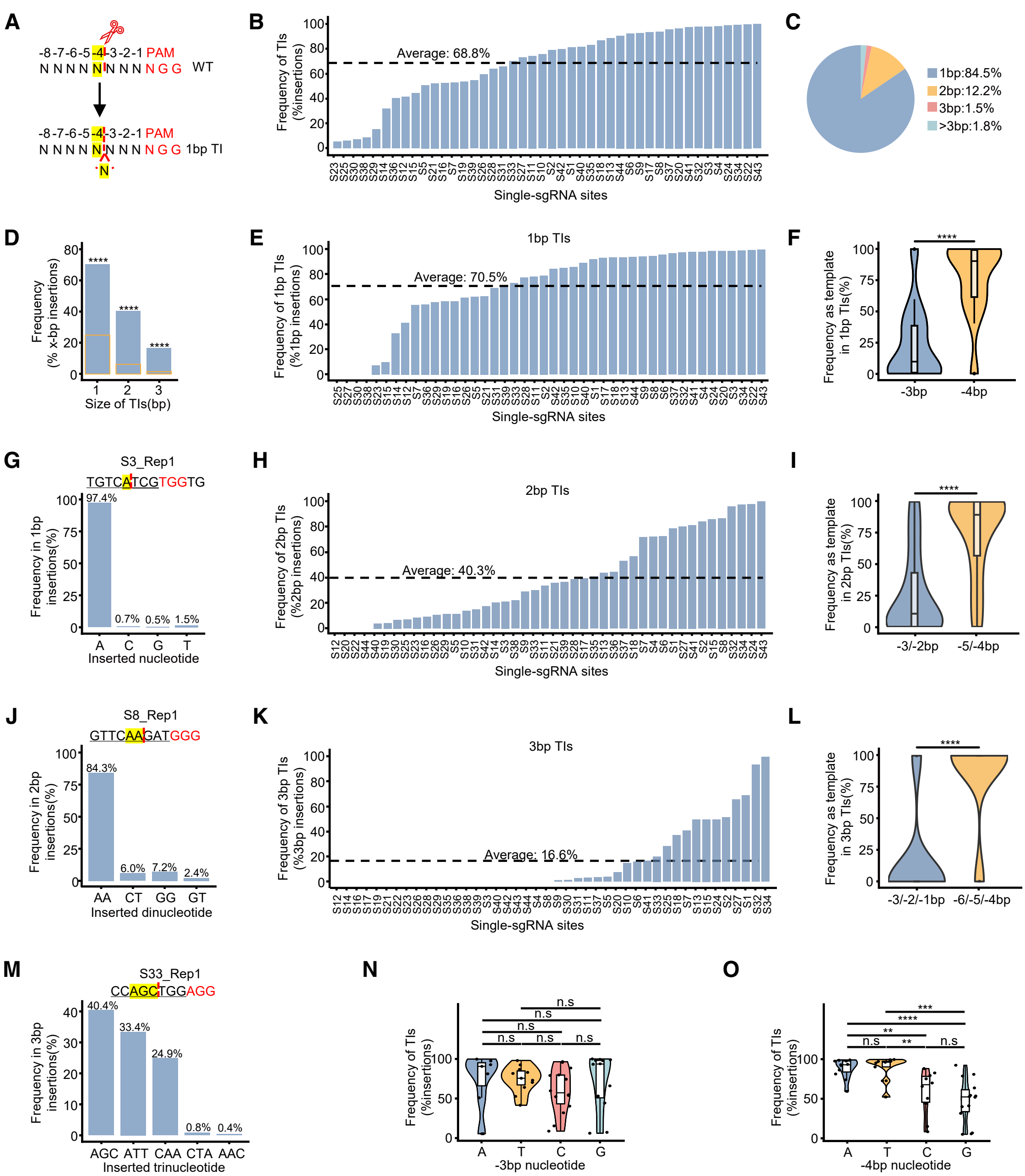

Figure 3. CRISPR-Cas9-mediated short insertions in MuSCs *in vivo* are templated and predictable

(A) Schematic illustration of 1 bp TI. The −4 bp position nucleotide functioning as the template is marked in yellow. (B) Frequency of TIs in all insertions at the canonical cut site across 44 single-sgRNA target sites. The average frequency of TIs across all sites is shown by the dashed line. (C) Pie chart showing the average frequency of TIs of varying sizes. (D) Frequency of 1, 2, and 3 bp TIs, with the orange bar representing the expected frequency for each TI category. (E) Frequency of 1 bp TIs in all 1 bp insertions for each target site. The average frequency of 1 bp TIs across all sites is shown by the dashed line. (F) Relative frequency of the upstream −3 or −4 bp nt as template in 1 bp TIs. (G) An example of 1 bp TIs on target S3. The bar plot illustrates the frequencies of inserted nucleotide being A, T, C, and G in 1 bp insertions. The nucleotide −4 bp upstream of the PAM is highlighted in yellow. The canonical cut site is indicated by a red dashed line, and the PAM is marked in red. (H) Frequency of 2 bp TIs in all 2 bp insertions for each target site. The average frequency of 2 bp TIs across all sites is shown by the dashed line. (I) Relative frequency of the −3 to −2 or −5 to −4 bp dinucleotides as templates in 2 bp TIs. (J) An example of 2 bp TIs on S8. The bar plot illustrates the frequencies of all detected dinucleotides in 2 bp insertions. The dinucleotides −5 to −4 bp upstream of the PAM are highlighted in yellow. (K) Frequency of 3 bp TIs in all 3 bp insertions for each target site. The average frequency of 3 bp TIs across all sites is shown by the dashed

(legend continued on next page)

a significantly lower frequency (Figures S3A–S3C). The frequency of 2–7 bp MH deletions was substantially higher than the background expectation (Figure 2C). Notably, we found that longer MH sizes were correlated with larger average deletion sizes (Figures 2D and S3C). Moreover, MH-mediated deletions exhibited distinct characteristics compared to non-MH deletions. First, the average size of MH deletions was larger (17 vs. 7 bp) (Figures 2E and 2F). Further unsupervised hierarchical clustering revealed that the length of MH deletions peaked at 10–20 bp, with 48% exceeding 10 bp (Figure 2G). Conversely, non-MH-mediated deletions produced smaller deletions, with approximately half (45%) being 1–2 bp deletions (Figure 2H).

It is reported that CRISPR-Cas9-induced deletions are asymmetric and occur unidirectionally downstream of the breakpoint[8]; thus, we analyzed the positioning patterns of the deletions relative to the canonical cut site by classifying deletions into three groups: downstream deletions occurring within the PAM-containing flank, upstream deletions occurring in the opposite flank, and spanning deletions across the canonical cut site (Figure 2I). Our results showed that among all the deletion events, on average, 56.0% spanned the cleavage site, followed by 32.3% upstream and 11.7% downstream deletions (Figure 2J). MH-mediated deletions exhibited a similar pattern (71.3%, 21.6%, and 7.1% spanning, upstream, and downstream deletions, respectively) (Figures 2K and S3D), indicating that most CRISPR-Cas9-induced *in vivo* deletions were not asymmetric, which contrasts with the *in vitro* finding.[8] Non-MH deletions exhibited a unidirectional distribution but interestingly had a preference for upstream rather than downstream deletions (Figure 2L), which is also different from the *in vitro* finding.[8] For instance, at the S3 locus, the top five MH deletions (accounting for 63.8% of total MH deletions) all spanned the cut site (Figure 2M). In contrast, the top five non-MH deletions (representing 49.0% of total non-MH deletions) primarily occurred upstream of the cut site (Figure 2N).

Finally, we examined the influence of chromatin context on deletion events and found no obvious association (Figures S3E and S3F). Altogether, the above results indicate that MH is prevalent in CRISPR-Cas9-induced deletions in MuSCs *in vivo* and that MH-mediated deletions display distinct features from non-MH-mediated deletions.

CRISPR-Cas9-mediated short insertions in MuSCs *in vivo* are templated and predictable

Next, we took a close examination of the insertions detected in *vivo*. *In vitro* studies have demonstrated that Cas9-mediated short insertions are precise and predictable even without donor templates, reflecting the error-free nature of NHEJ in the repair of Cas9-generated DSBs.[3,4,8,40] Specifically, the 1 bp insertion is predominantly templated by the nucleotide located upstream of the cleavage site (−4 bp upstream of the NGG PAM) (so-called TIs); thus, the inserted sequence tends to be identical to the −4 bp nucleotide (Figure 3A). We found the frequency of TIs in MuSCs *in vivo* varied significantly across the 44 sites, ranging from 5.5% to 100%, with an average of 68.8% (Figure 3B). Moreover, the frequency declined sharply as the insertion size increased, with a 1 bp TI being the predominant type (84.5%) and those larger than 3 bp rarely detected (1.8%) (Figures 3C and S4A). Furthermore, the frequency of TIs with short length (1, 2, or 3 bp) was significantly higher than the background expectation (Figure 3D).

As the predominant type, 1 bp TIs were detected at all 44 sites, accounting for 70.5% of the total 1 bp insertions (Figure 3E). As expected, −4 bp nucleotides were more frequently used as the template than −3 bp nucleotides (77.1% vs. 22.9%) (Figure 3F), indicating asymmetric templating as previously reported.[3,4,8,40] As a result, the 1 bp insertions tended to be identical to the −4 bp nucleotides; for example, when the base at the −4 bp is A at the S3 site, the inserted nucleotide is most likely to be A; 1 bp TIs using the −4 bp A as the template accounted for over 97.4% of all 1 bp insertions (Figure 3G). As for 2 bp TIs, although they represented only 40.3% of all 2 bp insertions (Figure 3H), a similar asymmetric templating was observed; the dinucleotides located upstream (−5/−4 bp relative to PAM) of the cut site were more frequently used as the repair template than the downstream (−3/−2 bp relative to PAM) sequences (74.5% vs. 25.5%) (Figure 3I). For example, as shown at the S8 site, 84.3% of all 2 bp insertions used the −5 to −4 bp AA as the template (Figure 3J). When the insertion size increased to 3 bp, the percentage of TIs decreased to 16.6% (Figure 3K), but asymmetric TIs still existed (86.4% for −6/−5/−4 bp vs. 13.6% for −3/−2/−1 bp) (Figure 3L). For instance, as shown at target S33, 40.4% of all 3 bp insertions used −6 to −4 bp AGC as the template (Figure 3M). As for TIs larger than 3 bp, the frequency was so rare that they were only detected at sporadic sites, as exemplified by a 5 bp TI at the S7 site (Figure S4B). Altogether, the above findings underscore the prevalence of TIs in CRISPR-Cas9-mediated insertions in MuSCs *in vivo*.

Lastly, we investigated potential factors associated with the formation of TIs. Overall, there was no clear correlation between the frequency of TIs and the TIF (Figure S4C), indicating that the proportion of TIs is an inherent feature of each target site, independent of editing efficiency. While the frequency of total insertions did not show a significant association with local chromatin features (Figure S4D), we found a striking negative correlation between TI frequency and active chromatin marked by ATAC-seq and H3K4me3/H3K27ac ChIP-seq signals (Figure S4E). When exploring the relationship between TI formation and local DNA context, we

line. (L) Relative frequency of the −3 to −1 or −6 to −4 bp trinucleotides as templates in 3 bp TIs. (M) An example of 3 bp TIs on S33. The trinucleotides −6 to −4 bp upstream of the PAM are highlighted in yellow. The bar plot illustrates the frequencies of all detected trinucleotides in 3 bp insertions at the canonical cut site for S33. (N and O) Frequency of TIs in all insertions at target sites with A, T, C, and G −3 (N) or −4 (O) bp upstream of the PAM. Statistical significance was calculated using a *t* test for (F), (I), (L), (N), and (O) and a one-tailed *t* test (alternative = "greater") for (D). $^{**}p < 0.01$, $^{***}p < 0.001$, $^{****}p < 0.0001$, and ns, no significance.

found that the base at the −3 bp position showed no significant association with TI frequency (Figure 3N). However, sites with a G at the −4 bp position exhibited significantly lower TI frequency than those with A or T but not C (Figure 3O). In sum, the above findings suggest that CRISPR-Cas9-mediated short insertions in MuSCs *in vivo* may be shaped by local DNA context at the DSB site.

Limited predicting power of the machine learning models *in vivo*

Our above findings demonstrate that the main principles governing CRISPR-Cas9-mediated editing *in vitro* are largely applicable *in vivo*. This prompted us to test whether algorithms trained by profiling a large set of Cas9 target sites *in vitro* could be used for *in silico* prediction of the editing outcomes in MuSCs *in vivo*. To this end, we evaluated the performance of three widely used machine learning models—inDelphi,[16] FORECasT,[17] and Lindel.[8] We categorized all the detected indels into five groups based on types and sizes (>2 bp deletions, 2 bp deletions, 1 bp deletions, 1 bp insertions, and >1 bp insertions) (Figure S5). For each target site, the three algorithms were applied to predict the frequency of each indel group and compared with our experimental results. Accordingly, only the 2 bp deletions predicted by inDelphi and the 1 bp insertions predicted by Lindel closely matched our experimental outcomes. For all other groups, the three models yielded frequencies that differed significantly from the experimentally measured values, indicating that *in vitro*-trained algorithms have limited predictive power in forecasting indel patterns in MuSCs *in vivo*.

In vivo mapping of CRISPR-Cas9-induced indel profiles in MuSCs using dual sgRNAs

Next, we examined the Cas9 editing outcomes using dual sgRNAs. It is known that a single-sgRNA-induced DSB can be repaired by NHEJ without editing, but Cas9 keeps cutting until an indel disrupts the target site.[4,5,7] Re-cutting can be prevented by employing two sgRNAs, as simultaneous editing can delete the intervening sequence so that the re-ligated sequence no longer aligns with the target sequences recognized by either sgRNA (Figure 4A). In our previous studies, the dual-sgRNA strategy has been frequently utilized to target a range of genomic regions to achieve successful deletion of various elements in MuSCs *in vivo*.[31,33,34] 51 pairs of sgRNAs were designed to target distinct genomic regions, including those edited in our previous studies,[31,33,34] numerically labeled D1–D51 (Figure 1A; Table S1). Among them, 30 dual sgRNAs targeted CDSs and promoter regions of 13 protein-coding genes, including *Atf4*, *Bcl6*, *Ctcf*, *Ent*, *Fos*, *Fosb*, *Junb*, *Myc*, *Myod1*, *Pknox2*, *Pax7*, *Runx1*, and *Sugt1*; 18 were designed to target potential enhancer regions; and 3 targeted non-regulatory intergenic regions to further increase the diversity and complexity of the targeted sites (Figures S6A and S6B; Table S1). Considering the distance between the two concurrent DSBs may influence Cas9 editing outcomes,[7] the designed dual sgRNAs were separated by varying intervals mostly within 1 kb (Figure 4B). Sequences encompassing the two DSBs in edited MuSCs or unedited controls were amplified and subjected to unbiased NGS to detect the indel profiles (Tables S2 and S5).

We found that dual sgRNAs yielded a wide range of TIFs, from 0.007% to 97.2%, on the 51 target sites (Figure 4C), with an average of 38.8%. Similar to the findings from the single-sgRNA group (Figure S1D), the TIFs positively correlated with active chromatin markers (Figure S6C), further confirming that active chromatin may facilitate Cas9-mediated editing. Based on the occurrence of DSBs at the two target sites, we then categorized the repair outcomes into three types: (1) both DSBs were formed simultaneously, resulting in the deletion of the intervening sequence; (2) only one of the two sites was edited; and (3) editing occurred at both sites but DSBs were formed sequentially; therefore, there was no removal of the interval sequence (Figure 4D). We found that type 2 indels accounted for 59.3% of the total indels on average, followed by 25.9% of type 3, while type 1 comprised only 14.8% (Figures 4E and S6D). Similar to the single-sgRNA group (Figure S1C), the frequency of each type was comparable across the two replicates (Figure S6E). Type 1 indels resulted from ligation of the two DSBs to reflect a straightforward indication of Cas9 cleavage activity, thus becoming the focus of our subsequent analyses. Jaccard similarity scoring of the top five most frequent indels for each site—which represented an average of 62.8% of all type 1 indels (Figures S6F and S6G)—revealed an average similarity score as high as 0.66 between the two replicates (Figure 4F; Table S4); this indicates that type 1 indel patterns were reproducible across replicates, reinforcing that Cas9-induced DNA repair in MuSCs is not random. Furthermore, the frequency of type 1 indels did not show a significant correlation with chromatin features (Figure S6H). Nevertheless, we found that both TIFs and type 1 frequency positively correlated with the distance between the two DSBs (Figure 4G), supporting that the distance influences Cas9-induced repair outcomes.[7]

Next, to investigate the precision of NHEJ, we categorized the type 1 indels into four subtypes, including insertion, deletion, accurate (accurate ligation of the two broken ends), and Ins+Del (indels with simultaneous deletions and insertions) (Figures 4H and S6I). Accurate indels reflect the precise nature of classical NHEJ,[7] and the frequency varied significantly across target sites, ranging from 0% to 78.3% (Figure S6I), with an average of 35.9%; high similarity was detected across the two replicates (Figure S6J). It has been reported that the formation of TIs also reflects the precise nature of NHEJ and that it suppresses the frequency of accurate ligation events[7]; accordingly, we observed an inverse correlation between the frequency of the 1–2 bp TIs and the accurate events (Figure 4I). Therefore, the frequency of precise NHEJ-mediated repair reached 44.8% when considering accurate and TIs together (Figure 4J). As exemplified at the D12 site, both accurate and TIs were present, representing 7.0% and 56.9% of type 1 indels, respectively (Figure 4K).

Next, we explored potential factors influencing NHEJ precision. Correlation analysis revealed that while the frequency of accurate or accurate+TI indels exhibited no obvious association with TIF (Figure S6K), a significant positive correlation with the frequency of type 1 indels (Figure 4L) was observed. Additionally, there were no correlations with chromatin features (Figure S6L) and the

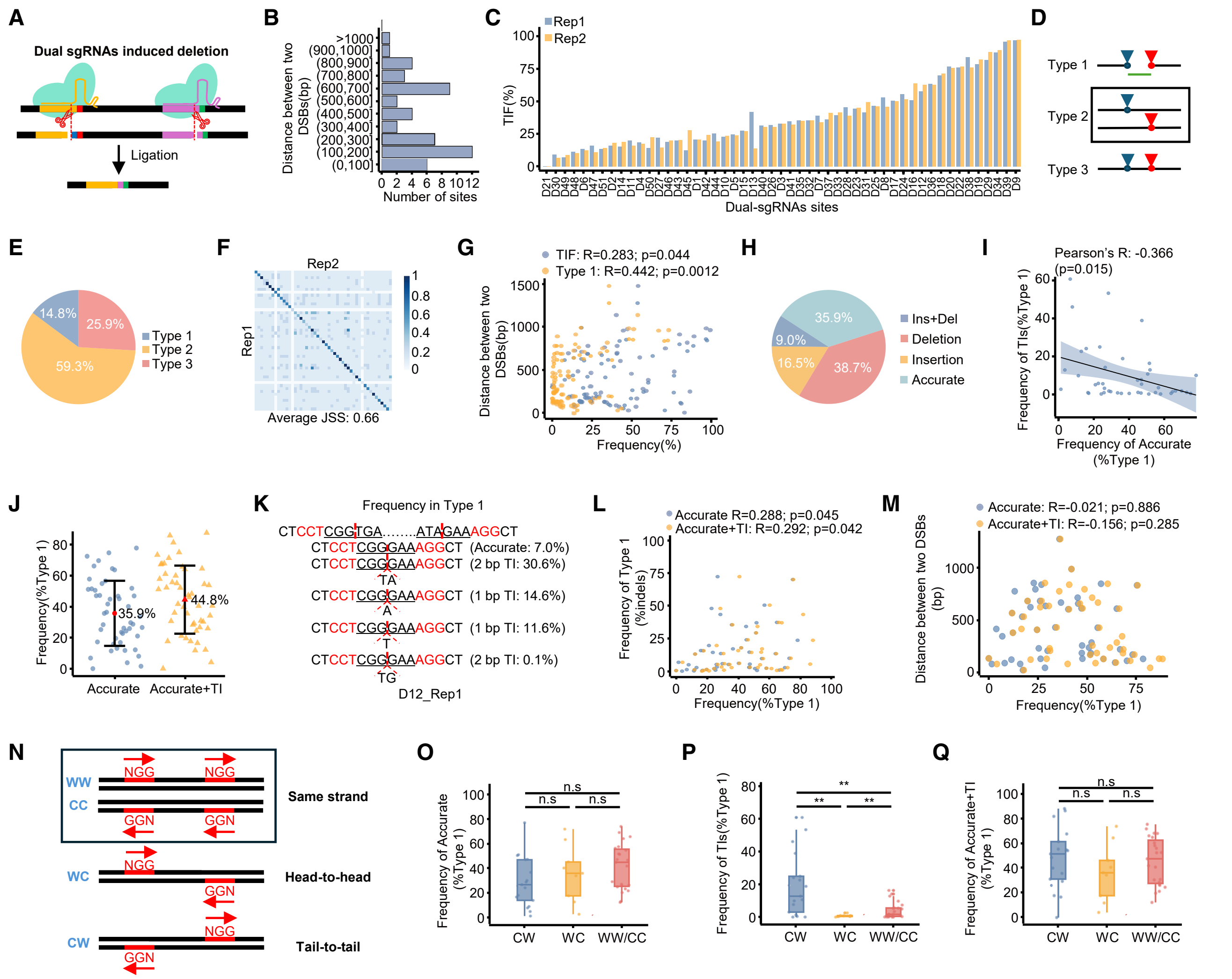

Figure 4. *In vivo* mapping of CRISPR-Cas9-induced indel profiles in MuSCs using dual sgRNAs

(A) Schematic illustration of dual-sgRNA-induced DNA deletion. (B) Distribution of distance between two DSBs across 51 sites. (C) TIFs for 51 dual-sgRNA targets with two biological replicates. (D) Schematic showing three types of dual-sgRNA-induced indels. (E) Pie chart showing the average proportion of type 1, type 2, and type 3 indels across all targets. (F) Heatmap of JSSs for type 1 indels. Each square indicates the JSS between the top five most frequent type 1 indels of the corresponding targets. The average JSS of paired replicates across all targets is shown. (G) Correlation of the distance between two DSBs and TIF (blue) or type 1 (orange) frequency. (H) Pie chart showing the average frequency of different subtypes of indels in the type 1 group. 49 targets with over 100 type 1 events were included for subtype analysis. (I) Pearson's correlation between the frequency of TIs and accurate indels in type 1 indels. Only 1 and 2 bp TIs were considered. (J) Frequency of accurate alone and accurate+TI in type 1 indels. The average frequency across all targets is shown. (K) An example on the D12 site showing the frequency of accurate, 1 bp TI, and 2 bp TI in type 1 indels. (L) Correlation of type 1 frequency with the frequency of accurate or accurate+TI in type 1 indels. (M) Correlation of distance between two DSBs and frequency of accurate (blue) or accurate+TI (orange). (N) Schematic showing four Cas9 PAM orientations for dual sgRNAs. WW, WC (head to head), CW (tail to tail), and CC orientations are determined by the recognition of Cas9 to the PAM on either the Watson strand (W) or the Crick strand (C). WW and CC are further classified as PAMs on the same strand. (O–Q) Boxplot illustrating the frequency of accurate (O), TIs (P), accurate+TI (Q) in total type 1 indels on targets with CW, WC, and WW/CC PAM orientation groups. Statistical significance was calculated using a *t* test for (O)–(Q). $^{**}p < 0.01$ and ns, no significance.

distance between the dual DSBs (Figure 4M). Lastly, we investigated whether the orientation of dual PAMs might have any influence. The orientation determined by the recognition of the PAM by Cas9 on either the Watson strand (W) or the Crick strand (C) can be arranged in three distinct combinations: WW/CC (on the same strand), WC (head to head), and CW (tail to tail) (Figures 4N and S6M). The WC PAM configuration is shown to be more effective in generating accurate ligation than the other two,[7] which was not observed in our study (Figure 4O). Interestingly, we found that the CW orientation yielded the highest TI frequency and the WC orientation produced the lowest (Figure 4P), but no significant differences were detected in accurate+TIs (Figure 4Q). We further categorized

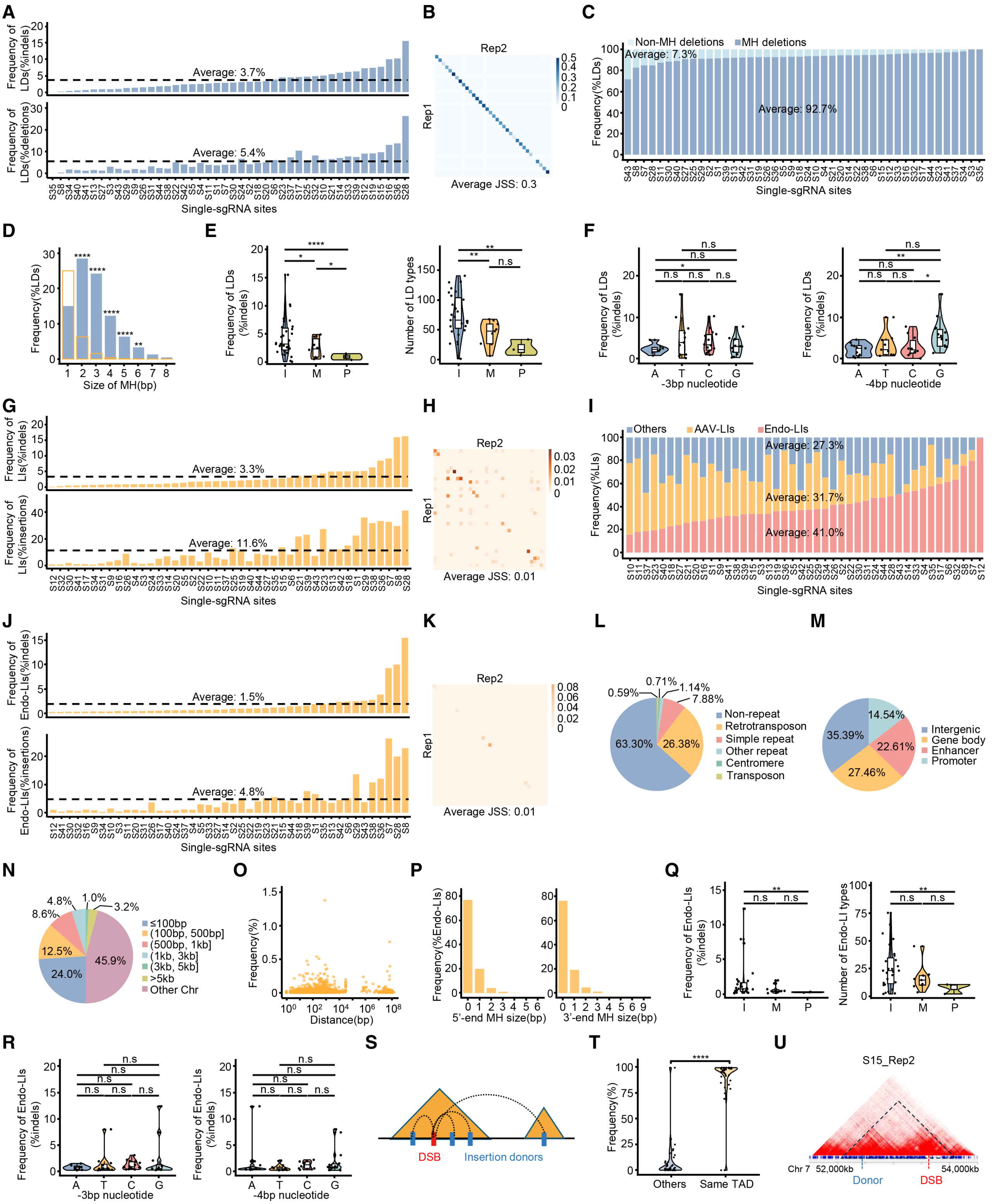

(legend on next page)

dual-sgRNA sites into four groups based on whether the PAM was located on the transcribed (T) or non-transcribed (NT) strand—TT, TN, NT, and NN (Figure S6N) —and found no significant difference in NHEJ precision (Figures S6O–S6R). These results suggest that PAM orientation configuration may not impact NHEJ precision *in vivo*.

Additionally, we examined type 1 deletions and found that, on average, 77.4% of type 1 deletions exhibited MH (Figure S7A), reinforcing that MH is widely used in deletion events. The length of the MH ranged from 1 to 10 bp, and the frequency of deletions was negatively correlated with MH size but higher than the background expectation for 2–5 bp MH (Figure S7B). Similar to the single-sgRNA group, the average size of type 1 MH deletions was larger than that of type 1 non-MH deletions (18 vs. 12 bp) (Figures S7C and S7D). Furthermore, unsupervised hierarchical clustering revealed that type 1 MH deletions were larger and peaked at 10–20 bp, with up to 61% exceeding 10 bp (Figure S7E). In contrast, type 1 non-MH-mediated deletions were generally smaller, with approximately 56% less than 10 bp (Figure S7F).

Altogether, the above results from analyzing dual-sgRNA-mediated Cas9 editing outcomes demonstrate that the repair of Cas9-induced DSBs by NHEJ is inherently precise in MuSCs *in vivo* and also substantiate the extensive role of MH in Cas9-induced deletions.

Analysis of CRISPR-Cas9-induced large on-target genomic modifications in MuSCs *in vivo*

Lastly, we focused our investigation on large-size genome modifications observed in our study (Figure 1H). Emerging evidence demonstrates the presence of CRISPR-Cas9-induced large-size genomic modifications, including LDs and LIs,[13,19,22] raising potential safety concerns in therapeutic genome editing. We first examined the LDs and LIs in the single-sgRNA group. Our results showed that LDs $\geq$ 50 bp accounted for an average of 3.7% of total indels and 5.4% of all deletion events (Figure 5A), and the frequency was highly reproducible across the two biological replicates (Figure S8A). The number of distinct LD types varied substantially across target sites (Figure S8B). Jaccard similarity scoring of the LD types for each site revealed only modest similarity between the two replicates (average score: 0.3) (Figure 5B; Table S4), indicating that LD formation may be largely random. Consistent with a previous study,[10] we found that 92.7% of LDs exhibited MH (Figure 5C), with a preference for longer MH tracts ($\geq$2 bp) (Figure 5D); for example, the top three most frequent LD types (50, 54, and 69 bp) at the S1 site were characterized by 3–4 bp MH (Figure S8C). Furthermore, LD frequency showed no significant association with TIF (Figure S8D) but was positively correlated with ATAC signals (Figure S8E). Given that longer indel size correlated with lower editing precision and precise sites tended to produce short indels (Figure 1N), we speculated that LD formation is associated with editing precision. As expected, the precise sites exhibited both a lower LD frequency and a reduced number of LD types (Figure 5E) than the other two groups. Furthermore, the frequency of LD formation was inversely correlated with the frequency of the top 1 most frequent indel (Figure S8F), which was utilized to assess editing precision (Figure 1I). Surprisingly, we found that the nucleotide composition flanking the cut site also influenced LD formation: while a G at the -3 bp position had no apparent effect on LD frequency (Figure 5F, left), a -4 bp G correlated with elevated LD frequency compared to A and C (Figure 5F, right).

For LIs $\geq$ 20 bp, they represented 3.3% of total indels and 11.6% of all insertion events (Figure 5G) and showed high reproducibility across replicates (Figure S8G); Also, the number of LIs varied substantially across all sites (Figure S8H). Unexpectedly, a minimal similarity in LI types was observed between the two replicates (average JSS: 0.01) (Figure 5H; Table S4), suggesting that the formation of LIs may be stochastic and highly complex. Further analysis of the origins of the inserted sequences revealed that the majority (72.7%) of LIs were templated, with 41.0% originating from the mouse genome

Figure 5. Analysis of CRISPR-Cas9-induced large on-target genomic modifications in MuSCs *in vivo*

(A) Frequency of $\geq$50 bp LDs for each single-sgRNA site, calculated as percentage in total indels (top) and total deletions (bottom). The average frequency of LDs across all sites is shown by the dashed line. (B) Heatmap of JSSs for LDs. Each square shows the JSS between all LDs of the corresponding targets. The average JSS of paired replicates across all targets is shown. (C) Frequency of MH- and non-MH-mediated LDs for each site. The average frequency across all sites is shown. (D) Frequency of LDs with MH of varying sizes. The orange bar represents the expected frequency for LDs with each MH size. (E) Violin plot illustrating the frequency of LDs (left) and number of LD types (right) in imprecise (I), middle (M), and precise (P) groups. (F) Frequency of LDs in total indels at sites with A, T, C, and G -3 (left) or -4 (right) bp upstream of the PAM. (G) Frequency of $\geq$20 bp LIs for each single-sgRNA site, calculated as percentage in total indels (top) and total insertions (bottom). The average frequency of LIs across all sites is shown by the dashed line. (H) Heatmap of JSSs for LIs. (I) Frequency of different types of LIs. The average frequency of each type across all sites is shown. (J) Frequency of $\geq$20 bp Endo-LIs for each single-sgRNA site, calculated as percentage in total indels (top) and total insertions (bottom). The average frequency is shown by the dashed line. (K) Heatmap of JSSs for Endo-LIs. (L) Pie chart showing the frequency of donor sequence categorized by types of repetitive elements for Endo-LIs. (M) Pie chart showing the frequency of donor sequence categorized by genomic annotations for Endo-LIs. (N) Pie chart showing distribution of distances between insertion donors for Endo-LIs and associated DSBs. (O) Scatterplot illustrating the distance distribution between donor sequences for Endo-LIs and their associated DSBs on the same chromosome. (P) Frequency of MH with varying sizes in 5' (left) and 3' (right) ends of Endo-LIs. (Q) Violin plot illustrating the frequency of Endo-LIs (left) and number of Endo-LI types (right) in the imprecise (I), middle (M), and precise (P) groups. (R) Frequency of Endo-LIs at sites with A, T, C, and G -3 (left) or -4 (right) bp upstream of the PAM. (S) Schematic showing co-localization of donor sequences and associated DSBs of Endo-LIs within the same TAD. (T) Violin plot showing significantly higher Endo-LI frequency of donor sequences originating from DSB-containing TADs compared to other loci on the same chromosome. (U) Hi-C contact map of S15 illustrating the influence of TAD on Endo-LI formation. The dashed triangle denotes the identified TAD. The red color in the Hi-C contact map indicates interaction intensity between genomic loci. The dark blue bar below the Hi-C contact map represents the genome browser view of gene tracks involved in the selected region. The positions of the donor and DSB are indicated by light blue and red dashed lines, respectively. Statistical significance was calculated using a *t* test for (E), (F), (Q), and (R), a paired *t* test for (T), and a one-tailed *t* test for (D). *$p < 0.05$, **$p < 0.01$, ****$p < 0.0001$, and ns, no significance.

(Endo-LIs) and 31.7% from the AAV vector (AAV-LIs) (Figure 5I). Integration from viral vectors has been extensively studied,[13,19] while Endo-LIs derived from the host genome remain understudied. Overall, the Endo-LIs accounted for 1.5% of the total indels and 4.8% of all insertions (Figure 5J); they exhibited characteristics similar to the total LIs, including a reproducible frequency between the two replicates (Figure S8I), variation in the number of types across sites (Figure S8J), and a lack of similarity in Endo-LI patterns (Figure 5K; Table S4). Notably, a fraction of Endo-LIs originated from repetitive sequences and fragile genomic regions, such as retrotransposons (26.38%) and centromeres (0.71%) (Figure 5L). Recent reports have suggested that DSB-induced insertions are associated with active transcription.[26] Consistently, 14.54% and 27.46% of the detected donor sequences came from promoters and gene body regions, respectively (Figure 5M). Moreover, we found that a subset of Endo-LIs (22.61%) originated from enhancer regions (Figure 5M), suggesting a possibility for them to modulate nearby gene expression. In general, over 98% of Endo-LIs originated from a single donor locus, while a small portion were derived from up to four genomic loci (Figure S8K). To examine whether Endo-LI formation results in deletion of the donor sequence, we selected two donor loci (one for S8 Rep#2 and one for S15 Rep#2) for targeted Deep-seq. After filtering out background indel noise identified in controls, no corresponding deletions were detected at these donor sites (Tables S1, S2, and S6). Nevertheless, we cannot definitively exclude the possibility that some Endo-LIs might result from the cutting and pasting of donor sequences. Regarding the distance from DSBs, 45.9% of Endo-LIs originated from different chromosomes (Figures 5N and S8L), and the other half were located on the same chromosome, particularly within 5 kb of the DSB (Figures 5N, 5O, and S8L).

Furthermore, no significant correlation was observed between the frequency of total LIs and Endo-LIs with TIF (Figures S8M and S8N) or chromatin features (Figures S8O). Further investigation of MH involvement revealed that about 76% of Endo-LIs showed no detectable MH at either the 5′ or 3′ end (Figure 5P), indicating that MH was not prevalently utilized in Endo-LI formation and that MMEJ may not be the predominant repair pathway for Endo-LIs. Similar to LD formation, an association between LI/Endo-LI formation and editing precision was observed (Figures 5Q, S8P, and S8Q): imprecise sites exhibited both a higher LI/Endo-LI frequency and an elevated number of LI/Endo-LI types (Figures 5Q and S8P) compared to the precise sites, suggesting that LI and Endo-LI abundance correlate with the I group. Additionally, the flanking sequence composition at the cut site had no effect on total and Endo-LI frequency (Figures 5R and S8R). It has been reported that insertions require physical contact between donor and acceptor loci,[26] and a recent study also showed that topologically associated domains (TADs) can restrict DSB-induced DNA replication inhibition[41]; thus, we examined the possible impact of TAD organization on Endo-LI formation (Figure 5S). By analyzing the Hi-C data from primary myoblasts,[42] we found that a significantly higher frequency (90.7% vs. 9.3%) of donor sequences and their associated DSBs originated from the same TAD (Figures 5T and S8S), as exemplified with S15 in Figure 5U, suggesting TAD organization may play a role in regulating Endo-LI formation.

Next, we examined the formation of large genome modifications in the dual-sgRNA group (Figure S9) and found that LDs accounted for an average of 2.3% of type 1 indels and 3.2% of type 1 deletion events (Figure S9A). Also, MH was prevalent during LD formation, with 87.3% of LDs exhibiting MH (Figure S9B), and these LDs preferentially utilized longer MH tracts ($\geq$2 bp) (Figure S9C), further underscoring the role of MMEJ in their generation. LIs constituted 1.1% of total indels and 6.4% of all insertion events (Figure S9D). 28.7% of LIs originated from the mouse genome, while 42.1% were derived from the AAV vector (Figure S9E). 38.07% of donor sequences of Endo-LIs were from fragile genomic regions, including retrotransposons (22.77%) and centromeres (0.99%) (Figure S9F), and 17.85% from enhancer regions (Figure S9G). Of note, the role of the 3D genome in Endo-LI formation was further supported by the observation that donor sequences and associated DSBs largely co-localize in the same TAD (Figures S9H and S9I).

Finally, to further characterize these large on-target modifications, we performed targeted individual DNA molecule sequencing (IDM-seq) based on Nanopore long-read sequencing with unique molecular identifiers (UMIs)[43,44] at four sites (S1, S7, S18, and S28) exhibiting moderate-to-high LD and LI frequencies (Figure S10A; Tables S1 and S7). The amplification range was extended to about 2,000 bp flanking the Cas9 cleavage site to maximize the detection, particularly of LDs (Figure S10B). As expected, IDM-seq detected elevated LD frequencies compared to short-read NGS (Figures S10C and S10D). In contrast, IDM-seq did not yield a higher proportion of LIs (Figures S10E and S10F). The LI patterns observed with short-read NGS were recapitulated by IDM-seq: specifically, LI types showed no similarity between replicates (Figure S10G), and the majority (91.4%) of LIs were templated, originating from the endogenous mouse genome (17.1%) or the AAV vector (74.3%) (Figure S10H). Notably, both methods detected comparable average proportions of Endo-LIs among total indels (Figures 5J and S10I). The patterns of Endo-LIs were also recapitulated: approximately 50% originated from repetitive sequences and fragile genomic regions (Figure S10J), correlated with active transcription (Figure S10K), and 23.35% derived from enhancer regions. Furthermore, 61% of the Endo-LIs originated from the same chromosome as the associated DSBs, predominantly within 3 kb (Figure S10L). The involvement of the 3D genome in Endo-LI formation was further supported by the observation that donor sequences and their corresponding DSBs largely co-localized within the same TAD (Figure S10M).

Altogether, the above results demonstrate that CRISPR-Cas9-induced large on-target genomic modifications can occur in MuSCs *in vivo*. LD formation requires the presence of MH and is influenced by the local DNA context, while the formation of Endo-LIs is affected by 3D chromatin architecture.

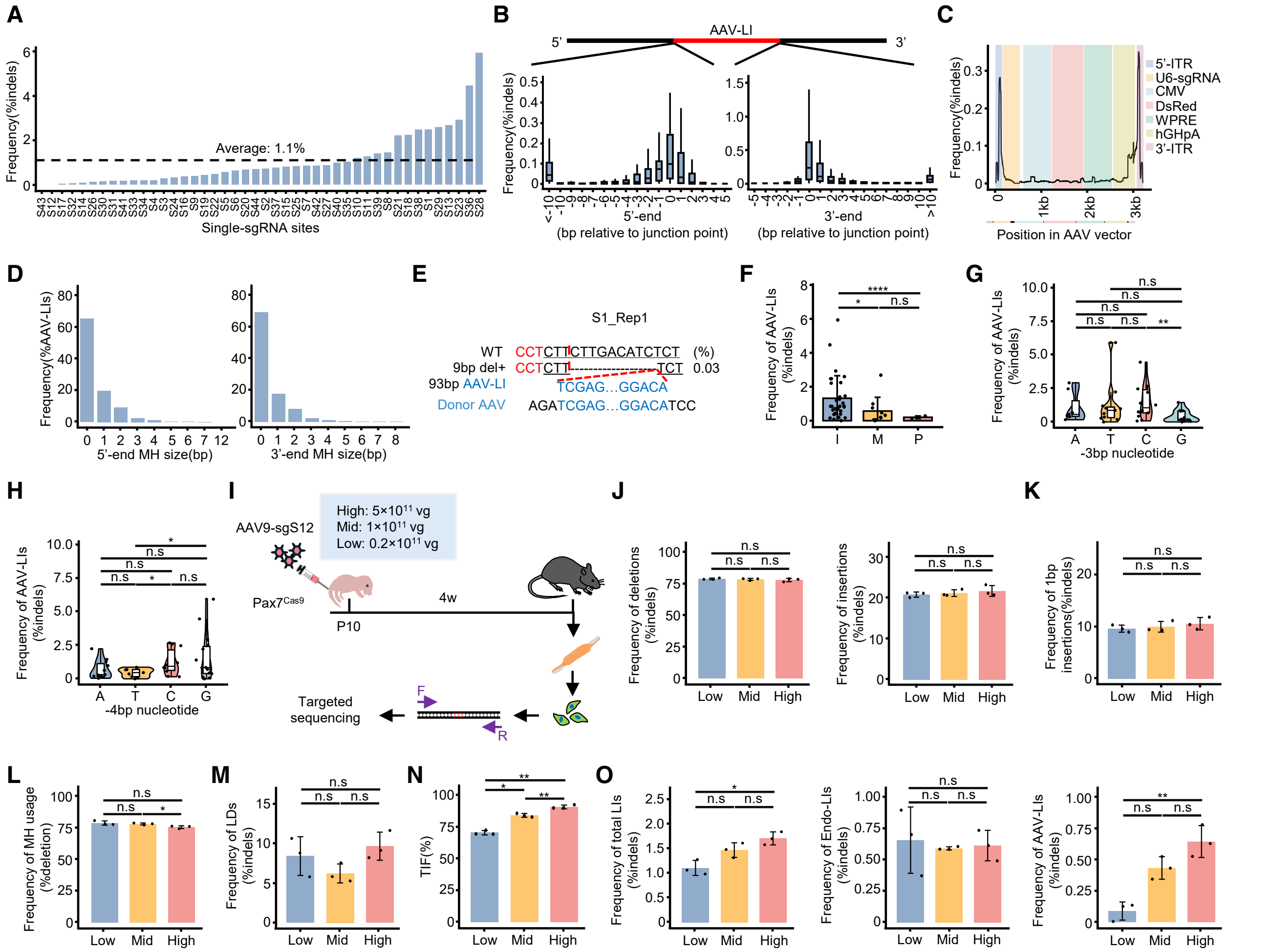

Figure 6. AAV integration is a general outcome of CRISPR-Cas9-induced editing in MuSCs *in vivo*

(A) Frequency of AAV-LI for each single-sgRNA site, calculated as a percentage of total indels. The average frequency of AAV-LIs across all sites is shown by the dashed line. (B) Distribution of 5′ and 3′ ends of AAV integration sites relative to the junction point. (C) The average inserted frequency of each AAV vector region. Each element of the AAV vector is presented with a designated color, and the position in the AAV vector is shown. (D) Frequency of MH with varying sizes in 5′ (left) and 3′ (right) ends of AAV-LI. (E) An example of AAV-LI on S1. A 93 bp AAV integration occurred together with a 9 bp deletion at the cut site. Frequency is presented as a percentage of total indels. The AAV-LI sequence and donor AAV sequence are marked in blue. (F) Violin plot illustrating the frequency of AAV-LI in I, M, and P groups. (G and H) Frequency of AAV-LI at sites with A, T, C, and G −3 (G) or −4 (H) bp upstream of the PAM. (I) Schematic showing experimental design for assessing the effect of AAV dosage on AAV-LI at the S12 site. High (5×10^{11} vg), mid (1×10^{11} vg), or low (0.2×10^{11} vg) dose of AAV9-sgRNA virus containing sgRNA targeting the S12 site was i.m. injected into $Pax7^{Cas9}$ mouse at P10 (n = 3), followed by MuSC isolation 4 weeks after injection. Sequences encompassing the S12 site were amplified and subjected to unbiased deep sequencing. (J–O) Barplot illustrating frequency of deletions (J, left), insertions (J, right), 1 bp insertions (K), MH usage in deletions (L), LDs (M), TIFs (N), total LIs (O, left), Endo-LIs (O, middle), and AAV-LIs (O, right) for the three dosage groups (n = 3 mice in each group). Statistical significance was calculated using a t test for (F)–(H) and a paired t test for (J)–(O). $^{*}p < 0.05$, $^{**}p < 0.01$, $^{****}p < 0.0001$, and ns, no significance.

AAV integration is a general outcome of CRISPR-Cas9-induced editing in MuSCs *in vivo*

We next focused our attention on exogenous AAV-LIs, which are emerging as a common phenomenon in AAV-delivered CRISPR-Cas9 editing.[9,29,30,45] AAV-LIs account for an average of 1.1% of total indels in the single-sgRNA group (Figure 6A). We suspect this may be underestimated because only integrations ≥20 bp were used in our analysis. Similarly, the frequency of AAV-LIs was highly consistent across the two replicates (Figure S11A). Moreover, insertion positions at both the 5′ and 3′ ends were predominantly located adjacent to the CRISPR-Cas9 cut site (Figure 6B), indicating these integrations were indeed generated by CRISPR-Cas9-induced DNA breaks.

Previous studies have reported that the inverted terminal repeat (ITR) region of AAV is the hotspot for vector integration.[29] Accordingly, we found that the ITR elements at both ends of the AAV vector were more frequently enriched in AAV-LI reads compared to other vector elements such as the U6-sgRNA cassette, CMV-dsRed cassette, WPRE,

and hGHpA (Figure 6C). Similar to total LIs, the frequency of AAV-LIs showed no significant correlation with TIF (Figure S11B) or chromatin features (Figure S11C). Regarding the role of MH in AAV-LIs, we observed that the majority (>65%) of AAV-LIs contained no MH at the 5′ or 3′ end (Figure 6D), indicating that MH may not be essential for AAV integration. This was exemplified at the S1 site: a 93 bp fragment of the AAV vector was integrated at the cut site, accompanied by a 9 bp deletion, but no MH was detected at either end of the AAV-LI (Figure 6E). Additionally, the frequency of AAV-LIs was negatively correlated with editing precision, with precise sites exhibiting a significantly lower average frequency than the I group (Figure 6F). The sequence context flanking the cut site also influenced AAV-LIs: a G at the −3 bp position was associated with reduced integration compared to C (Figure 6G), whereas a G at the −4 bp position showed enhanced AAV-LI frequency compared to T (Figure 6H).

As AAV dosage is a crucial consideration in AAV-mediated gene therapy,[28] we examined the AAV dosage effect on AAV integration leveraging our previously collected sequencing data performed on the single-sgRNA S12 site (located in the CDS region of the *Myod* locus) using low (0.2×10^{11} vg), medium (1×10^{11} vg), or high (5×10^{11} vg) AAV doses[31] (Figure 6I). Reanalysis of the data (Tables S2 and S8) revealed that increasing the AAV dose did not alter the overall indel profile. Specifically, the frequency of deletions (Figure 6J, left), insertions (Figure 6J, right), 1 bp insertions (Figure 6K), MH usage in deletions (Figure 6L), and LDs (Figure 6M) remained largely unaffected across different doses. However, increasing the AAV dose indeed enhanced the TIF (Figure 6N). As expected, the overall frequency of total LIs was significantly higher in the high-dose compared to the low-dose group (Figure 6O, left), primarily driven by elevated AAV integration (Figure 6O, right), whereas the frequency of Endo-LIs remained largely unchanged (Figure 6O, middle). The above findings suggest that while increasing AAV dosage can enhance editing efficiency in AAV-mediated CRISPR-Cas9 *in vivo* editing, it may introduce unwanted risks by elevating the frequency of AAV integration.

The dual-sgRNA group was also examined by aligning AAV sequences with sequencing reads. We found that AAV integration occurred at all 51 sites, with an average frequency of 0.6% of total indels (Figure S11D), and was comparable across replicates (Figure S11E). Consistent with the single-sgRNA group (Figure 6C), most of the mapping reads included the ITR element (Figure S11F). Furthermore, increasing AAV dosage was associated with higher TIF frequency (Figures S11G and S11H; Tables S2 and S8) and led to a significant increase in the frequency of type 1 and type 3 but not type 2 events (Figure S11I). We reason that the formation of type 1 and type 3 requires the combined action of two sgRNAs, which may be facilitated by the increased AAV-sgRNA dose. However, the dosage did not affect the frequency of accurate NHEJ events within the type 1 indels, as the levels of accurate and accurate+TI were comparable among the three dosage groups (Figures S11J and S11K). As expected, increasing the dosage from low to high elevated AAV-LI formation (Figure S11L).

In summary, our findings demonstrate that AAV integration is a commonly detected outcome of CRISPR-Cas9-induced editing in MuSCs *in vivo*, highlighting the need to take caution when using AAV-mediated CRISPR-Cas9 therapy *in vivo*.

In vivo perturbation of NHEJ/MMEJ pathways can modulate CRISPR-Cas9 editing in MuSCs

Given that NHEJ and MMEJ pathways dominate repair outcomes in the absence of exogenous donor templates during *in vivo* genome editing, we next investigated their contributions to large on-target modifications by depleting key components of each pathway (Figure 7A). To this end, as shown in Figure 7B, AAV9-dual sgRNA (8×10^{11} vg) targeting key factors of NHEJ (*Ku80* and *Lig4*) or MMEJ (*Nbn* and *Polq*) was i.m. injected into $Pax7^{Cas9}$ mice at P5; AAV9-single sgRNA (5×10^{11} vg) was administered at P10 to target the above studied S29 site, which exhibited moderate levels of LDs (Figure 5A) and LIs (Figures 5G, 5J, and 6A). MuSCs were isolated 4 weeks later, and a 2- to 7-fold decrease in target protein abundance was confirmed (Figure 7C).

Targeted sequencing was performed to examine how each perturbation altered editing outcomes (Tables S2 and S9). Notably, only the loss of KU80, but not others, significantly increased the TIF (Figure 7D). Both KU80 and NBN depletion, but not LIG4 or POLQ, led to a marked reduction in the frequency of deletions among total indels (Figure S12A). As expected, 1 bp indels—typically a hallmark of NHEJ after Cas9-induced DSBs[46]—were significantly decreased following KU80 or LIG4 depletion but increased upon NBN or POLQ loss (Figure 7E). Conversely, loss of LIG4 enhanced the average usage of MH in deletions, hinting at elevated MMEJ activity (Figure 7F), but POLQ depletion had the opposite effect. KU80 or NBN loss also led to expected decreases and increases, respectively, although without statistical significance (Figure 7F). These findings suggest that disrupting these factors *in vivo* can modulate the activity of their respective repair pathway.

We next assessed how the perturbations influenced the formation of large on-target DNA modifications. Disruption of the NHEJ components KU80 or LIG4 significantly increased the frequency of LDs; NBN depletion decreased LD occurrence, but POLQ loss had minimal impact despite its established role in promoting LDs *in vitro*[10] (Figure 7G). Additionally, loss of LIG4 increased the average deletion size, but NBN depletion caused the opposite effect (Figure 7H). The effects on LIs were complex (Figures 7I–7K): KU80 and NBN loss elevated both the frequency of total LIs (Figure 7I) and AAV-LIs (Figure 7K) without affecting the proportion of Endo-LIs (Figure 7J). POLQ depletion had a minimal impact on total LIs, Endo-LIs, or AAV-LIs (Figures 7I–7K). Interestingly, removal of LIG4 did not alter total LIs (Figure 7I) but significantly increased Endo-LIs (Figure 7J) and decreased AAV-LIs (Figure 7K). Moreover, none of the perturbations affected the overall insertion size or the size of endogenous insertions (Figures S12B and S12C). Collectively, our results demonstrate

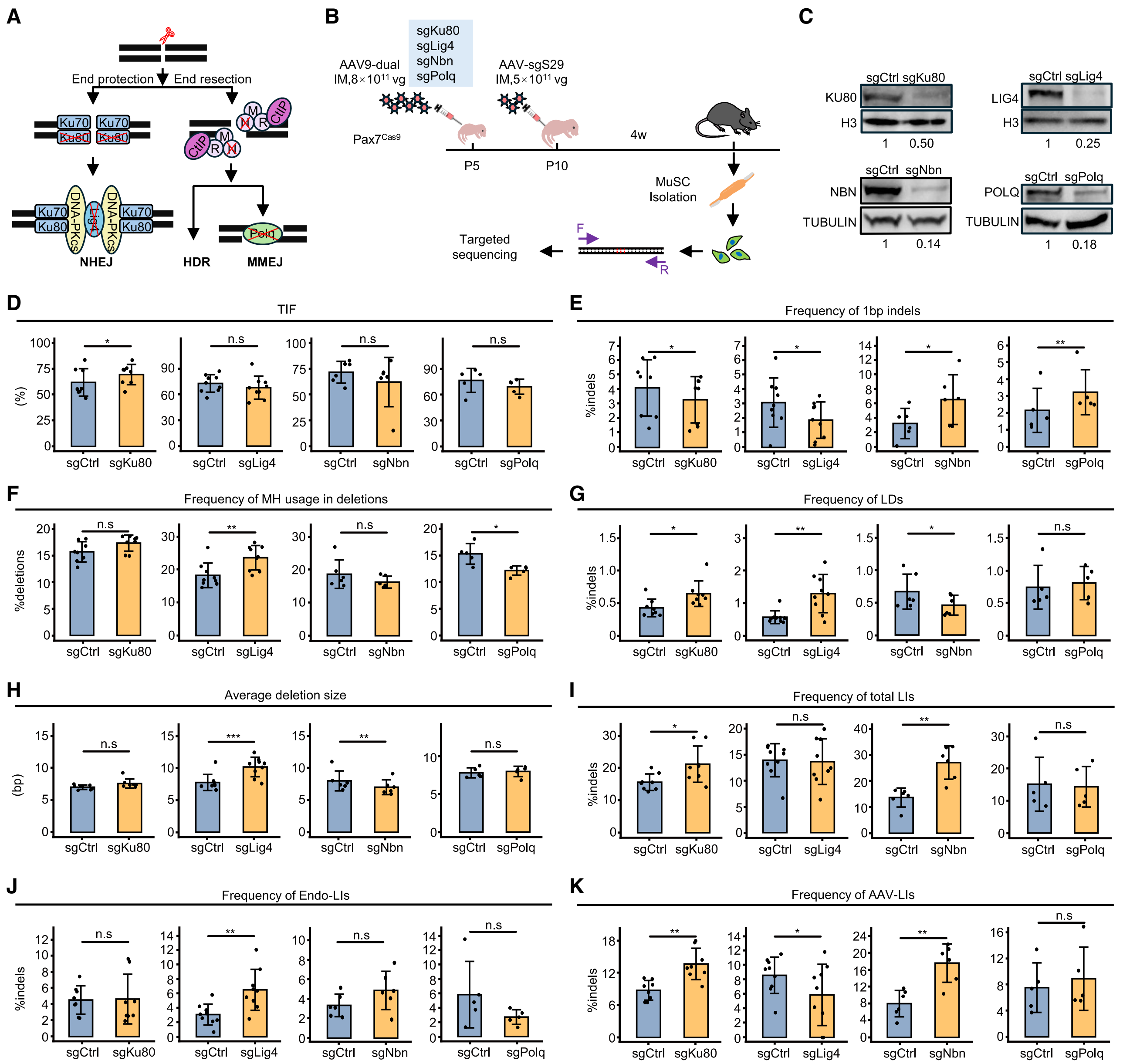

Figure 7. *In vivo* perturbation of NHEJ/MMEJ pathways can modulate CRISPR-Cas9 editing in MuSCs

(A) Schematic illustration of CRISPR-Cas9-induced DSB repair pathways. Key proteins in each pathway are shown, and proteins selected for *in vivo* deletion are marked in red. (B) Schematic showing experimental design for deleting the above proteins in MuSCs *in vivo*. 8×10^{11} vg of AAV9-dual-sgRNA virus targeting the CDS regions for *Ku80*, *Lig4*, *Nbn*, or *Polq* were i.m. injected into Pax7^{Cas9} mouse at P5, respectively. 5×10^{11} vg of AAV9-single sgRNA was administrated at P10 to target the S29 site. MuSCs were isolated 4 weeks after injection, and sequences encompassing the S29 site were amplified and subjected to unbiased deep sequencing. (C) Protein depletion of KU80, LIG4, NBN, or POLQ in the above MuSCs was confirmed by western blotting. (D–K) TIFs (D), frequency of 1 bp indels (E), MH usage (MH size $\geq$ 2) in deletions (F), LDs (G), total LIs (I), Endo-LIs (J), AAV-LIs (K), and average deletion size (H) after each protein depletion are shown. Statistical significance was calculated using a paired *t* test for (D)–(K) (n = 5–9 in each group). *$p < 0.05$, **$p < 0.01$, ***$p < 0.001$, and ns, no significance.

that dampening NHEJ activity promotes LD formation, whereas suppressing MMEJ causes the opposite effect. Furthermore, distinct NHEJ and MMEJ factors differentially influence LI generation, suggesting a more complex mechanistic basis for LI formation *in vivo*.

DISCUSSION

In this study, by systematically investigating the editing patterns at 95 sites in MuSCs *in vivo*, we revealed the general principles of CRISPR-Cas9-mediated *in vivo* editing. Our results indicate that the repair outcomes of CRISPR-Cas9-generated DSBs in MuSCs *in vivo* largely

align with those observed *in vitro*. Large on-target genome modifications are widely present, representing an important class of editing byproducts that may pose potential risks for CRISPR-Cas9-mediated therapies. Furthermore, by depleting key factors in NHEJ and MMEJ pathways, we uncovered their distinct roles in the generation of these undesirable byproducts.

In vivo CRISPR-Cas9 editing in MuSCs follows *in vitro* principles

Our results indicate that the main rules of CRISPR-Cas9-induced indel signatures derived from *in vitro* studies generally persist *in vivo*—such as the non-random nature of indel profiles (Figure 1E), the prevalence of MH in deletions (Figures 2B and S7A), and the preference of NHEJ for precise editing (Figures 3 and 4). However, we also observed notable differences between *in vitro* and *in vivo* editing. For example, the directionality of deletions *in vivo* is distinct. A previous *in vitro* study using massively parallel synthetic reporters containing a sgRNA expression cassette together with its PAM-endowed target sequence demonstrates that both MH- and non-MH-mediated deletions exhibit asymmetric characteristics, predominantly occurring unidirectionally downstream of the breakpoint,[8] but we observed that the overall deletions (Figure 2J) and MH-mediated deletions (Figure 2K) preferred to span the cut sites. In contrast, non-MH-mediated deletions show a higher tendency to occur upstream (Figure 2L). Nevertheless, it is known that the repair profile depends strongly on cell type/cell state[47]; the observed discrepancies between *in vivo* and *in vitro* comparisons in our study may arise from distinct cellular contexts rather than purely reflecting inherent differences between *in vivo* and *in vitro* systems.

NHEJ-mediated CRISPR-Cas9-induced DSB repair *in vivo* is precise and sequence-context dependent

Our findings uncover that deletions predominate the landscape of CRISPR-Cas9-induced indels in MuSCs (Figure 1D). Both MMEJ and NHEJ can lead to deletions, and NHEJ is thought to be primarily responsible for generating small deletions,[5] typically within 2 bp, whereas larger deletions are mainly formed by MH-mediated MMEJ. Most deletions in our analysis are accompanied by MH (Figures 2B and S7A), implying that MMEJ could be the predominant pathway for these deletions. Despite high reproducibility across replicates (Figure 1E), increased deletion rates potentially compromise editing precision to some extent (Figure 1O). In contrast, NHEJ-mediated small insertions in our study tended to be accurate, often manifested as TIs (Figures 3 and 4). In CRISPR-Cas9-related *ex vivo* gene therapies, enhancing HDR levels by simultaneously inhibiting MMEJ and NHEJ is a commonly used strategy to increase the editing precision.[5] *In vivo* CRISPR-Cas9-based therapies, on the other hand, primarily rely on the NHEJ and MMEJ pathways, since HDR is largely inactive in terminally differentiated cells or requires an exogenous repair template.[9] Compared to MMEJ, NHEJ tends to produce smaller and more predictable outcomes, making it a safer option for *in vivo* editing; thus, strategies to enhance accurate NHEJ may be beneficial for *in vivo* therapies. For example, the Plk3 inhibitor GW843682X can enhance accurate NHEJ by repressing CtIP-dependent end resection.[7]

On the other hand, due to the cleavage flexibility of the RuvC domain, Cas9 can produce both blunt and staggered ends.[3,39] Previous studies have shown that blunt ends, upon repair, may result in random deletions, template-independent insertions, or restoration of the wild-type (WT) sequence.[3] In contrast, the generation of an overhang typically results in TIs through NHEJ repair. Emerging studies have further demonstrated that the type of end generated is closely related to the DNA sequence context near the breakpoint.[3,39] Increasing evidence suggests that when the nucleotide −4 bp upstream of the PAM is G, the RuvC domain is more inclined to cleave the non-target DNA strand to produce blunt DSBs, whereas a G at −3 bp is more frequently linked to staggered DSBs.[15,16,39] Consistent with these observations, our results show that target sites harboring a −3 bp G were generally associated with enhanced editing precision (Figure 1P) and reduced frequency of AAV integration (Figure 6G). In contrast, a −4 bp G displayed the opposite pattern, characterized by reduced editing precision (Figure 1Q), decreased proportion of TIs (Figure 3O), and elevated frequency of both LDs (Figure 5F, right) and AAV integration (Figure 6H). Therefore, during *in vivo* editing, alongside enhancing NHEJ activity, selecting target sites with specific sequence features could increase the generation of staggered DSBs, further improving editing precision.

Widespread occurrence of large on-target genome modifications in CRISPR-Cas9-mediated *in vivo* editing in MuSCs

Our study reveals widespread occurrence of large on-target modifications during *in vivo* editing.

Although Cas9-mediated genome editing holds great promise for the treatment of genetic diseases, unintended on-target modifications (Figure S13), including LDs and LIs, have been progressively reported[10,13,23] but mostly on sporadic sites *in vitro*. Our work demonstrates that these large on-target modifications are also prevalent during *in vivo* editing. Among these modifications, LDs are the most extensively studied. Their occurrence can result in the loss of large DNA fragments or even, in some cases, the removal of entire chromosomes.[21,22] In 92.7% of the LDs analyzed (Figure 5C), we observed the presence of MH, implicating the involvement of the MMEJ pathway in their formation, which aligns with recent studies.[10,13] Of note, the indels were detected 4 weeks after sgRNA transduction in MuSCs, suggesting that these undesirable mutations can persist stably.

Current studies on LIs were primarily focused on the integration of exogenous DNA vectors, such as AAV.[13,29,30] As one of the most widely utilized delivery platforms in clinical gene therapy, AAV integration at target sites has been reported during *in vivo* editing at sporadic sites, with integration rates at certain loci reaching as high as 47% of all indels.[29] Our results indicated that AAV integration is also a common phenomenon across the wide range of sites during *in vivo* editing in MuSCs (Figures 6A and S11D), with the ITR region identified as a hotspot for integration (Figures 6C and S11F). Notably, the AAV integration accounted for only 1.1% of all indels

on average, with the highest being only 5.9% (Figure 6A). Several factors may contribute to this discrepancy from the *in vitro* finding, including differences in cell types, AAV dosage, and, most notably, analytical criteria. In our analysis, we considered only AAV integrations greater than 20 bp as a subset of LIs, which may cause the underestimation. Our findings thus reinforce the notion that the potential safety concerns of AAV integration remain to be thoroughly evaluated; the use of non-viral vectors should be considered for the transfer of gene editing agents *in vivo*, such as lipid nanoparticles (LNPs) for Cas9 mRNA delivery.[28]

Our study presents the systematic characterization of Endo-LIs (Figure S13). Previous studies have shown that a portion of LIs can also originate from the host genome,[13,23] but there remains a significant knowledge gap in understanding these Endo-LIs. Among the LIs detected in the single-sgRNA group, 41.0% of the donor sequences were derived from the host genome (Figure 5I). Notably, a subset of Endo-LIs sourced from enhancer regions (Figures 5M, S9G, and S10K), suggesting their potential role in disrupting gene regulation. For example, if DSBs occur near oncogenes, such insertions could enhance oncogene expression, potentially contributing to tumorigenesis. Further analysis of these Endo-LI types revealed no reproducibility across biological replicates (Figure 5K), indicating a high degree of randomness or complexity in their generation. Nevertheless, we found that the 3D genome architecture influenced the formation of these insertions. Specifically, insertions originating from the same chromosome were more likely to reside within the same TAD as the acceptor site (Figures 5T, S9I, and S10M). This observation aligns with the expectation that endogenous insertion events typically require physical proximity between donor and acceptor loci[26,41]; it is also consistent with a recent study showing that TAD can restrict DSB-induced DNA replication inhibition.[41] Future studies are necessary to elucidate the mechanisms underlying the formation of these Endo-LIs and assess their potential safety risks.

Differential roles of NHEJ/MMEJ in generating large on-target genome modifications in MuSCs *in vivo*

Despite the increasing attention on Cas9-generated large gene modifications,[13,20,23] the mechanisms underlying their formation remain elusive. Here, we depleted four key factors essential for NHEJ and MMEJ to investigate their involvement. Our findings indicate that depleting KU80 or LIG4 to reduce NHEJ activity resulted in increased LD formation (Figure 7G). Similarly, depleting the key protein NBN involved in MMEJ resulted in decreased LD frequency (Figure 7G). Interestingly, while *in vitro* studies have shown that the knockout or inhibition of POLQ significantly reduces LD formation,[10] we did not observe such a phenomenon *in vivo* (Figure 7G), although we noted a reduction in MH-mediated deletions following POLQ loss (Figure 7F). Moreover, while inactivation of NHEJ/MMEJ-related genes led to qualitatively expected changes in the indel patterns (Figures 7E and 7F), the impact was only moderate. This may be attributed to partial, not complete, protein depletion (Figure 7C), which retained residual DNA repair pathway activity, or, alternatively, to differences in DNA repair pathways between *in vivo* and *in vitro* contexts. In terms of LIs, their formation appears to be more complex. The loss of KU80 and NBN both resulted in an increased frequency of total LIs (Figure 7I), likely due to elevated AAV integration (Figure 7K), but the formation of Endo-LIs remained unchanged (Figure 7J). Of note, because an AAV-dual-sgRNA strategy was used to knock out these factors, we cannot rule out the possibility that variation in AAV transduction efficiency among mice caused changes in AAV integration. While studies have indicated that both MMEJ and NHEJ are involved in the random integration of exogenous vectors, with POLQ playing a crucial role,[24,25] we only observed a reduction in AAV integration following the loss of LIG4 (Figure 7K). However, LIG4 loss also led to increased frequency of Endo-LIs, suggesting that LIG4 has distinct roles in exogenous DNA integration and Endo-LI formation, and these processes might occur through different mechanisms. Based on these findings, our work suggests that enhancing NHEJ activity—particularly in the absence of AAV as a vector—represents a viable strategy to minimize the formation of both LDs and Endo-LIs.

Potential strategies to improve genome editing precision *in vivo*

Given that on-target byproducts arise from the repair of DSBs, CRISPR-based technologies that do not induce DSBs, such as base editing and prime editing,[1,19] are likely to avoid such unintended genomic alterations. However, these tools are typically limited to correcting small-scale or single-point mutations and often require larger editing reagents, posing significant delivery challenges.[22] Consequently, CRISPR-Cas9, despite its DSB dependency, is expected to continue expanding in clinical applications. Minimizing DSB-induced genomic aberrations is therefore crucial for reducing potential genotoxicity in patients. Our findings provide actionable insights for improving the safety of CRISPR-Cas9-mediated *in vivo* therapies. First, whenever feasible, non-viral delivery methods, such as LNP-mediated Cas9 mRNA delivery, should be prioritized to avoid integration of exogenous DNA vectors and reduce off-target effects.[28] Second, an elaborate design of sgRNAs by selecting target sites with specific sequence context could increase the likelihood of generating staggered DSBs, which are associated with more predictable repair outcomes.[3] Third, co-delivering small-molecule drugs that enhance NHEJ activity could help to reduce the occurrence of LDs and Endo-LIs, confining edits to more precise DNA regions. Combining these strategies with the use of high-fidelity Cas9 variants to reduce off-target effects could significantly improve the safety of CRISPR-Cas9-based therapies *in vivo*.

We should point out the limitations of our study. First, although such large on-target modifications were detected, their functional consequence remains unexplored; it is unknown whether they could compromise cellular functionality or promote tumorigenesis. Investigating the temporal dynamics of these large indels will be essential to determine their long-term effects. As CRISPR-Cas9-based therapies are already advancing into clinical trials, there is an urgent need for comprehensive safety assessments to balance the risks of gene editing with the pressing need to treat serious diseases. Second, as pointed out, our CRISPR-Cas9/AAV9-sgRNA system edits

proliferating myoblasts during postnatal myogenesis, and a proportion of the edited myoblasts might differentiate and fuse to form myofibers; thus, a portion of the genomic "scars" generated by Cas9 likely propagates into functional muscle tissue, which could potentially introduce biases in the analysis of indel patterns. Lastly, our study was conducted exclusively in MuSCs. It remains unclear whether the findings are applicable to other non-dividing or terminally differentiated cells *in vivo*. For example, since Cas9 is also expressed in post-mitotic myonuclei, these nuclei can undergo editing as well. Indeed, Cas9 has been shown to mediate efficient editing in non-dividing myonuclei.[48] Given that cell state influences Cas9 repair profiles,[47] it is plausible that the activity of DNA repair pathways differs between cycling myoblasts and post-mitotic myonuclei, causing different indel patterns. Further investigation across diverse cellular states will be necessary to gain a comprehensive understanding of CRISPR-Cas9-mediated *in vivo* editing outcomes.

MATERIALS AND METHODS

Mice

All animal experiments were conducted in accordance with the guidelines for the use of laboratory animals established by the Chinese University of Hong Kong (CUHK) and were approved by the Animal Experimentation Ethics Committee (AEEC) of CUHK. Mice were housed in the CUHK Animal Facility under standard conditions with a 12 h light/12 h dark cycle. The Cre-dependent *Rosa26*$^{Cas9\text{-}EGFP}$ knockin mice (B6;129-Gt(ROSA)26Sor$^{tm1(CAG\text{-}cas9\star\text{-}EGFP)}$ Fezh/J; stock number 024857) were obtained from the Jackson Laboratory. To generate Cas9 knockin mice, homozygous Pax7^{Cre} mice were crossed with Rosa26$^{Cas9\text{-}EGFP}$ mice, as previously described.[31,32,34]

AAV9 virus production, purification, and injection

The AAV9 virus was produced as previously described.[31,32] Briefly, human embryonic kidney (HEK)293FT cells were seeded in T75 flasks and transfected at 80%–90% confluency with a 1:1:2 ratio of AAV9-sgRNA vector (5 μg), AAV9 serotype plasmid (5 μg), and pDF6 helper plasmid (10 μg) using polyethyleneimine. After 24 h, the cells were switched to growth medium (Dulbecco's modified Eagle's medium [DMEM] supplemented with fetal bovine serum [FBS], 100 U/mL penicillin, 100 μg/mL streptomycin, and 2 mM L-glutamine) for an additional 48 h of culture. Cells were then harvested and washed twice with sterile phosphate-buffered saline (PBS). To release the AAV9 virus, the cell pellet was resuspended in AAV lysis buffer (50 mM Tris-HCl [pH 8.0] and 150 mM NaCl) and subjected to three sequential freeze-thaw cycles (liquid nitrogen/37°C). The lysates were treated with Benzonase (Sigma-Aldrich) and $MgCl_2$ (final concentration: 1.6 mM) at 37°C for 30 min, followed by centrifugation at 3,000 rpm for 10 min. The supernatant was mixed with 1/4 volume of a 5× PEG/NaCl solution (40% PEG8000 [w/v]; 2.5 M NaCl) to precipitate the virus at 4°C overnight. After centrifugation at 4,000*g* for 30 min at 4°C, the pellet was resuspended in sterile PBS and centrifuged again at 3,000*g* for 10 min. The supernatant was filtered through a 0.22 μm sterile filter and passed through a 100 kDa molecular weight cutoff filter (Millipore). The concentrated solution was washed three times with sterile PBS. The titer of the AAV9 virus was determined by quantitative reverse transcription polymerase chain reaction (RT-qPCR) using primers targeting the CMV promoter. The sequences of the primers used are listed in Table S10. To profile CRISPR-Cas9-induced editing patterns, 5×10^{11} vg of AAV9-sgRNA or AAV9-dual sgRNA was i.m. injected into heterozygous Pax7^{Cas9} mice at P10. To assess the effect of AAV dosage on AAV integration, 1×10^{12}, 5×10^{11}, or 1×10^{11} vg of AAV9-dual-sgRNA virus targeting the D16 site were injected i.m. into Pax7^{Cas9} mice at P10. To evaluate the effects of NHEJ/MMEJ on the formation of CRISPR-Cas9-induced large on-target modifications, 8×10^{11} vg of AAV9-dual-sgRNA virus targeting the CDS regions of *Ku80*, *Lig4*, *Nbn*, and *Polq* was injected i.m. into Pax7^{Cas9} mice at P5, respectively. For the control group, the same dose of AAV9 virus containing the pAAV9-sgRNA backbone without any sgRNA insertion was injected. Then, 5×10^{11} vg of AAV9-single sgRNA was administered at P10 to target the S29 site. MuSCs were isolated 4 weeks after the AAV injection.

MuSC isolation by fluorescence-activated cell sorting

MuSCs were isolated by fluorescence-activated cell sorting (FACS) based on established protocols.[49] Briefly, hindlimb muscles from Pax7^{Cas9} mice were collected and digested with collagenase II (1,000 U/mL; Worthington) for 90 min at 37°C. The tissue suspension was then washed in washing medium (Ham's F-10 medium [Sigma-Aldrich], 10% heat-inactivated horse serum [HIHS] [Gibco], and penicillin/streptomycin [1×, Gibco]). MuSCs were further liberated by collagenase II (1,000 U/mL) and dispase (11 U/mL) treatment for 40 min at 37°C. Mononuclear cells were filtered through a 40 μm cell strainer, and GFP+ MuSCs were sorted using a BD FACSAria Fusion cell sorter (BD Biosciences).

Cell culture

HEK293FT cells (ATCC, CRL-3216) were cultured in DMEM supplemented with 10% FBS, 100 U/mL penicillin, and 100 μg/mL streptomycin in a 5% CO_2 humidified incubator at 37°C.

Plasmids

Site-specific sgRNAs were designed using the CRISPOR web tool (http://crispor.tefor.net/).[50] To minimize off-target effects, we exclusively selected sgRNAs with a specificity score higher than 50, a threshold considered by CRISPOR to indicate high specificity. Oligonucleotides encoding the guide sequences were synthesized and cloned into the AAV9-sgRNA transfer vector (AAV: ITR-U6-sgRNA(backbone)-CMV-dsRed-WPRE-hGHpA-ITR) via the Sap I site. For dual-sgRNA plasmid generation, the second sgRNA cassette containing the U6 promoter and guide sequence was inserted into the AAV9-sgRNA vector using Xba I and Kpn I restriction sites. All sgRNA sequences are provided in Table S10.

Western blotting

Western blotting was performed as previously described.[31,34,51,52] The following dilutions of antibodies were used for each antibody: Histone H3 (Santa Cruz Biotechnology, sc-517576, 1:3,000),

α-TUBULIN (Santa Cruz Biotechnology, sc-23948, 1:2,000), KU80 (Proteintech, 16389-1-AP, 1:1,000), LIG4 (Cell Signaling Technology, 14649S, 1:1,000), NBN (Cell Signaling Technology, 3002S, 1:1,000), and POLQ (Cell Signaling Technology, 48160S, 1:1,000). The source data for Western blotting are provided in Figure S14.

Targeted Deep-seq

Targeted Deep-seq was performed as previously described.[31] Briefly, genomic DNA from unedited MuSCs and MuSCs infected with AAV9-sgRNA or AAV9-dual sgRNA was amplified using the Q5 High-Fidelity 2× Master Mix (NEB). PCR products were purified using the QIAquick PCR Purification Kit (Qiagen) and subjected to library preparation with the NEBNext Ultra II DNA Library Preparation Kit (NEB). Barcoded libraries were pooled and sequenced on the Illumina HiSeq 1500 or NovaSeq X platform. The primers used for Deep-seq are listed in Table S1.

IDM-seq

IDM-seq was performed as previously described.[43,44] Briefly, target DNA molecules were labeled using a UMI-containing oligonucleotide pool comprising three functional regions: a 3′ gene-specific sequence for targeting the edited locus, a central random UMI sequence for unique molecule tagging, and a 5′ universal primer binding site, facilitating uniform amplification of all UMI-labeled DNA molecules for Nanopore long-read sequencing. Genomic DNA from unedited MuSCs or MuSCs infected with AAV9-sgRNA was subjected to UMI labeling via one round of UMI oligonucleotide priming, using the following thermal cycling program: initial denaturation at 98°C for 70 s, gradient annealing from 70°C to 65°C at a ramp rate of 1°C/5 s, extension at 72°C for 7 min, and a final hold at 4°C. The UMI-labeled DNA was purified using 0.8× DNA clean beads (Vazyme, N411-01) to remove unlabeled UMI oligonucleotides, then mixed with a universal primer and a target site-specific reverse primer for amplification with Q5 High-Fidelity 2× Master Mix (NEB). The resulting amplicon was further purified and used for Nanopore library preparation with the Ligation Sequencing Kit (Oxford Nanopore Technologies) according to the manufacturer's standard protocol, followed by sequencing on the Nanopore platform. The sequences of the primers used are listed in Table S10.

ChIP-seq

ChIP-seq was performed as previously described.[33,34] Briefly, about 5×10^5 cells were cross-linked with 1% formaldehyde for 10 min at room temperature and quenched with 125 mM glycine. Cells were washed, pelleted by centrifugation (700*g*, 5 min, 4°C), flash-frozen in liquid nitrogen, and stored at −80°C. The cell pellet was resuspended in 130 μL of shearing buffer (10 mM Tris-HCl [pH 8.0], 0.1% SDS, 1 mM EDTA, and 1× protease inhibitor cocktail [PIC]) and sonicated using a Covaris S220 (140 W intensity, 5% duty cycle, 200 cycles per burst, 7 min). The lysate was adjusted to a final concentration of 150 mM NaCl and 1% Triton X-100, then clarified by centrifugation (20,000*g*, 20 min, 4°C). To reduce non-specific binding, the supernatant was pre-cleared by incubation with pre-washed Dynabeads Protein G (Invitrogen, 10004D) for 2 h at 4°C. A 1/50 volume of the cleared lysate was reserved as the input control. The remaining lysate was incubated overnight at 4°C with 0.75 μg of the appropriate antibody. The following day, 10 μL of pre-washed Dynabeads Protein G was added to each reaction in immunoprecipitation (IP) buffer (10 mM Tris-HCl [pH 8.0], 150 mM NaCl, 0.1% SDS, 1 mM EDTA, 1% Triton X-100, and 1× PIC) and incubated for an additional 2 h at 4°C. Beads were washed sequentially: twice with IP buffer, twice with high-salt wash buffer (10 mM Tris-HCl [pH 8.0], 500 mM NaCl, 0.1% SDS, 1 mM EDTA, and 1% Triton X-100), twice with LiCl wash buffer (10 mM Tris-HCl [pH 8.0], 250 mM LiCl, 1 mM EDTA, 0.5% NP-40, and 0.5% sodium deoxycholate), and once with cold TE buffer (10 mM Tris-HCl [pH 8.0], 1 mM EDTA, and 50 mM NaCl). Immunocomplexes were eluted in ChIP elution buffer (50 mM Tris-HCl [pH 8.0], 10 mM EDTA, and 1% SDS) at 65°C for 30 min, and cross-links were reversed by incubation at 65°C for 16 h. For the input sample, three volumes of ChIP elution buffer were added, and samples were processed in parallel with the IP samples. DNA was purified following treatment with RNase A (0.2 mg/mL, 37°C, 2 h) and Proteinase K (0.2 mg/mL, 55°C, 3 h) via phenol:chloroform extraction and ethanol precipitation, then resuspended in 1× TE buffer. ChIP-seq libraries were prepared using the NEBNext Ultra II DNA Library Preparation Kit (NEB, E7645S) and sequenced on an Illumina NovaSeq X platform (150 bp paired-end reads). The following antibodies were used: H3K4me3 (Cell Signaling Technology, #9751), H3K27ac (Cell Signaling Technology, #8173), and H3K27me3 (Cell Signaling Technology, #9733).

ATAC-seq

ATAC-seq was performed using the Hyperactive ATAC-Seq Library Prep Kit (Vazyme, TD711-01) according to the manufacturer's standard protocol and sequenced on an Illumina NovaSeq X platform (150 bp paired-end reads).

Variant detection for single-sgRNA target sites

All sequencing data for edited single-sgRNA groups were generated in-house, except for sample S12, which was sourced from a published paper.[31] In-house scripts were developed to extract NGS reads containing target-specific primer sequences. Quality filtering was performed using Trimmomatic (v.0.39)[53] with the parameter AVGQUAL:30 to eliminate low-quality reads. In instances where paired reads exhibited significant quality disparities, only the higher-quality end was retained. The processed reads were then analyzed using Sequence Interrogation and Quantification (SIQ) (v.1.3)[54] through a two-stage workflow: (1) merging overlapping paired-end reads and (2) aligning the merged consensus sequences to target references for variant detection, utilizing default parameters (Min support: 2; Max base error: 0.08; TINS search distance: 100). While most analyses relied on paired-end data, single-end processing was employed in three specific scenarios: (1) when targets exceeded 300 bp, (2) in cases of insufficient merge efficiency, or (3) due to substantial quality discordance between read pairs (Table S2). The outcomes were classified into the following categories: WT, single-nucleotide variant

(SNV), insertion, deletion, delins (an insertion coinciding with a deletion), TINS (a deletion accompanied by an insertion sourced from the adjacent sequence within 100 bp of the cut site), tandem duplication (TD), and TD compound (a TD that incorporates additional insertions). For each event, the fraction, position, and size of indels, as well as the MH for deletions, were provided. In terms of relative mutation positioning, SIQ defines the canonical cut site as position 0, with positions to the left of the cut site assigned negative values and those to the right assigned positive values. Given that targets vary in length, we ensured a fair evaluation of on-target editing by defining a 60 bp window centered on the canonical cut site for all target sites. Events overlapping with this window (delRelativeStart $\leq$ 30 and delRelativeEnd ≥ -30) were included in the subsequent analysis, with their corresponding fractions adjusted accordingly. The mutant amplicon we analyzed ranged from 40 to 290 bp. Indel events—including insertion, deletion, delins, TINS, TD, and TD compound—were classified as edited events, while SNVs were categorized as WT events. The mutations from control samples were excluded from downstream analyses. Target genomic locations were displayed using the R package "RIdeogram."[55]

Definition and quantitative analysis of editing precision

Precision at a target site was defined by the average frequency of the most common indel among total edited events across two biological replicates. All targets were subsequently categorized into three groups based on precision: imprecise ($\leq$0.25), medium (0.25 < middle $\leq$ 0.5), and precise (>0.5).[37] To assess the number of indel types within each precision group, only events with frequencies in total edited events exceeding 0.005 were included. This approach was taken to reduce bias resulting from differences in sequencing depths. The number of indel types for each target was averaged across two biological replicates. To evaluate indel size within each precision group, deletions were defined as negative values and insertions as positive. The absolute indel size was calculated as |delSize + insSize|. These sizes were weighted by their frequencies in total edited events and summed to obtain the final indel size for each replicate. Subsequently, the final indel sizes were averaged across two biological replicates for each target. Statistical significance was assessed using a *t* test.

Processing and correlation analysis of epigenomic features

ATAC-seq and ChIP-seq reads were trimmed using Trim Galore (v.0.6.10) (https://github.com/FelixKrueger/TrimGalore) and aligned to the mm10 genome using BWA (v.0.7.17-r1188).[56] Reads with a mapping quality < 30 and duplicate reads were removed using SAMtools (v.1.5)[57] and Sambamba (v.1.0.0).[58] To improve peak-calling accuracy, blacklisted regions (ENCODE mm10 v.2)[59] were removed using BEDTools.[60] Tn5 offsets were corrected for ATAC-seq data using alignmentSieve (deepTools v.3.5.5).[61] Peak calling was performed using MACS2 (v.2.2.6).[62] To calculate the average signal intensity over target regions, only replicate 1 of the epigenomic data was used. The BAM files were converted to BigWig format using BamCoverage (v.3.5.5)[61] with RPGC normalization. Subsequently, input-subtracted BigWig files were generated using bigwigCompare (v.3.5.5)[61] to account for background signal. Next, bigWigAverageOverBed (v.2)[63] was employed to calculate the average intensity over bases, treating non-covered bases as zeroes in each target. Spearman's correlation was calculated between the average intensity of each target and its corresponding average TIF across replicates for all targets. Correlations between average intensity and other variables were computed similarly.

Quantitative and directional analysis of MH-mediated deletions

The expected ratio for each k-mer size in MH was calculated as 0.25^k. The average deletion size for k-bp MH deletions was determined by weighting the sizes of the k-bp MH deletions based on their frequencies among the total k-bp MH deletions and summing these values to obtain a final size. The final k-bp MH deletion sizes were then averaged across two biological replicates for each target. Statistical significance was evaluated using a *t* test.

SIQ designated position 0 as the canonical cut site, with negative values assigned to left-side positions and positive values to right-side positions. In directional analysis, deletions were classified as downstream (within the PAM-containing flank), upstream (opposite flank), or spanning (crossing the cut site).

Resolution of TIs at single-sgRNA target sites

The frequency of TIs in k-bp insertions without deletions at the cut site (SIQ-reported relative start position 0) was determined by aligning the inserted sequences to reference segments of equal length. These segments extended either (1) rightward from position −3 or (2) leftward from position −4 relative to the NGG PAM (Figure 3A). When inserted nucleotides generate or occur within repetitive sequences at the cut site (e.g., a "C" adjacent to another "C" forming "CC" or a "G" extending "GG" to "GGG"), the exact insertion position becomes ambiguous. Consequently, SIQ may assign relative start positions as 0, 1, or 2. To address this uncertainty, these events were manually curated. For the allele frequency heatmap visualization, both reference and inserted alleles were standardized to the NGG PAM orientation. The expected ratio for each k-mer size in TI was calculated as 0.25^k.

Variant detection for dual-sgRNA target sites and characterization of editing outcomes

All sequencing data for edited dual-sgRNA groups were generated in-house, except for samples D5 and D32, which were sourced from a published paper.[31] For data analysis, primer-specific reads were extracted, and quality control was performed on the NGS data for each dual-sgRNA site (Table S1), following the same workflow applied to single-sgRNA sites. Next, a two-stage alignment approach, adapted from Chakrabarti et al.,[37] was implemented. In the initial stage, reads were aligned to the reference genome using BBMap (v.39.01).[64] During this phase, reads that aligned as proper pairs were discarded, and only the remaining reads were retained for further analysis using SAMtools (v.1.6).[57] In the second stage, these remaining reads were aligned to the reference genome using BBMap (v.39.01)[64] with a threshold that permitted indels of up to

3,000 bp. Finally, unmapped reads and secondary alignments were filtered out utilizing SAMtools (v.1.6)[57] and Sambamba (v.1.0.0).[58] Indels were detected and characterized using R scripts adapted from Chakrabarti et al.[37] with positions and lengths determined through CIGAR string parsing. For each target's two cut sites (3 bp upstream of NGG PAMs), indels overlapping the ±10 bp window surrounding either cut site were analyzed, with background indels from control samples removed. Reads containing these indels were classified as edited reads and categorized into three types: type 1, which includes indels spanning the windows of both cut sites; type 2, consisting of indels that overlap the window of only one cut site; and type 3, involving concurrent indels that overlap the windows of both cut sites without spanning. Type 1 is prioritized over the other types. Unedited reads were defined as (1) quality-trimmed reads before alignment, excluding the remaining reads from the initial alignment, and (2) reads whose indels fell completely outside both cut site windows. For type 1 analysis, type 1 reads were extracted to SIQ (v.1.3),[54] and precisely ligated sequences were utilized as references to explore the accurate ligation (Table S2). For subsequent analysis, only targets with a total event count exceeding 100 were considered. SNVs and WT were classified as accurate events, and PAM orientations were assigned as "W" for NGG and "C" for CCN. For TI analysis, 1 bp TIs were defined as insertions matching either the −3 or −4 nt upstream of either PAM. 2 bp TIs were defined as insertions that matched either the −3:−2 or −5:-4 dinucleotides of either PAM or any of the four combinations consisting of one nucleotide from the −3 or −4 position of PAM1 and one nucleotide from the −3 or −4 position of PAM2.

Comprehensive analysis of the origins and characteristics of LIs

Parallel alignments were conducted using BWA (v.0.7.17-r1188)[56] to the AAV vector sequence and the mm10 reference genome. LIs were processed through distinct alignment pipelines based on size: (1) for LIs $\leq$ 70 bp, bwa-aln was used followed by bwa-samse, and (2) for LIs > 70 bp, bwa-mem was employed. All alignments were filtered using SAMtools (v.1.6)[57] to eliminate unmapped reads and secondary alignments. When LIs were simultaneously mapped to both the mm10 and AAV sequences, the origin from mm10 took priority over that from AAV. LI sequences that did not map to either reference were classified as "others."

For functional annotations of Endo-LIs, genomic locations of Endo-LI donors were annotated using the R package "ChIPseeker,"[65,66] with those within ±3 kb of transcription start sites categorized as promoters. En-LI donor locations overlapping with H3K27ac peaks were designated as enhancers, with enhancers prioritized over other functional categories. The downstream category was classified as intergenic. For repetitive element annotation, the RepeatMasker output for the mm10 assembly was combined with the mm10 gap file, both obtained from the UCSC Genome Browser. Centromeres and satellites were classified together as centromeres. Long interspersed element (LINE), long terminal repeat (LTR), and short interspersed nuclear element (SINE) elements were grouped as retrotransposons. To resolve overlaps, the following priority hierarchy was employed: retrotransposons > transposons > centromeres > simple repeats > other repeats.

To analyze MH usage in Endo-LIs/AAV-LIs, custom R scripts were developed for detection. For each LI, the acceptor and donor site locations were identified within their respective reference genomes. Extended sequences flanking both sides of these sites were then extracted. Subsequently, the sequence consistency between the donor and acceptor was assessed by comparing extensions ranging from 1 to 15 nt.

For analyzing LIs within TAD, the processed HIC file of WT proliferating myoblasts from GEO: GSE157339[42] was used. TADs were identified at a 40k resolution using Juicer Tools (v.2.20.00.ac)[67] with the arrowhead method and the -k KR parameter. Since the HIC file was based on mm9, LiftOver[68] was utilized to convert the TAD locations to mm10. In our analysis, only LIs with a donor and acceptor located on the same chromosome were considered. The intersect command from BEDTools (v.2.27.1)[60] was used to determine whether the donor and acceptor were situated within consistent TADs, followed by a paired *t* test to evaluate significance. The visualization of the TAD heatmap was generated using the mm9 assembly.

Comparison of LD-, LI-, and endo-LI-type counts across precision groups and JSS analysis

To reduce sequencing depth bias, only LD, LI, and Endo-LI types constituting at least 0.01% of total indels and originating from targets with more than 10,000 total indels in both replicates were included. The counts of these LD, LI, and Endo-LI types were then compared across the I, M, and P groups in Figures 5E, 5Q, and S8P. For JSS analyses in Figures 5B, 5H, and 5K, all LD, LI, and Endo-LI types that met these criteria were compared between samples.

Variant analysis with IDM-seq

Nanopore R10 sequencing data were processed by filtering for reads with a quality score $\geq$ 10. High-quality reads were further screened using custom scripts to extract sequences containing both flanking primers. The VAULT tool[43] was utilized with default parameters for variant analysis, applying an additional length filter of 50–20,000 bp. Among filtered variants, all SNVs and any indels falling outside a ±30 bp window of the cut site were re-classified as WT. For indels overlapping this window, variants were categorized as insertions if the insertion length exceeded the deletion length and as deletions if the deletion length was greater. Specifically, insertions $\geq$ 20 bp were classified as LIs, while deletions $\geq$ 50 bp were classified as LDs. For the four edited samples, background indels identified in control samples were excluded. The reference amplicon size is from 1,928 to 2,039 bp. The mutant amplicon we analyzed ranged from 115 to 19,996 bp.

Evaluation of prediction tools on *in vivo* repair outcomes

Three commonly used prediction tools were employed: inDelPhi,[16] ForeCAST,[17] and Lindel.[8] For inDelPhi, parameters were set to the

mesenchymal embryonic stem cell (mESC) cell type, 0.05% minimal frequency, and a default indel length range. The complete target sequences used in SIQ served as input data for the analyses of inDelPhi and ForeCAST. For Lindel, the input consisted of 65 bp, including 30 bp upstream and 35 bp downstream of the cleavage site. Indels were classified into five categories: 1 bp insertions, >1 bp insertions, 1 bp deletions, 2 bp deletions, and $\geq$3 bp deletions. Predictions were statistically compared to SIQ measurements (averaged across two replicates per target) using *t* tests.

DATA AND CODE AVAILABILITY

All sequencing data generated in this study have been deposited in the GEO database under the accession codes GEO: GSE303540, GSE327391, and GSE327392.

ACKNOWLEDGMENTS

This work was supported by the Non-Communicable Chronic Disease-National Science and Technology Major Project of China to H.W. (project code: 2024ZD0530400); the National Key R&D Program of China to H.W. (project code: 2022YFA0806003); research funds from the Health@InnoHK program launched by the Innovation Technology Commission, the Government of HK, to H.W.; the Health and Medical Research Fund (HMRF) from the Health Bureau of HK to H.W. (project codes: 10210906 and 08190626); the Theme-based Research Scheme (TRS) from Research Grants Council (RGC) (project code: T13-602/21-N); the General Research Fund (GRF) from the RGC of the HK Special Administrative Region, China, to H.W. (project codes: 14108225, 14105123, 14105823, and 14103522); the National Natural Science Foundation of China (NSFC) to H.W. (project codes: 82172436 and 31871304); and the Area of Excellence Scheme (AoE) from RGC to H.W. (project code: AoE/M-402/20).

AUTHOR CONTRIBUTIONS

Conceived and designed the experiments, H.W., H.S., L.H., and Y.F.; performed the experiments, L.H., Z.W., and Q.Z.; analyzed the data, Y.F.; wrote the paper, H.W., L.H., and Y.F.; reviewed and edited the manuscript, H.W., H.S., L.H., and Y.F.

DECLARATION OF INTERESTS

The authors declare that they have no conflicts of interest.

SUPPLEMENTAL INFORMATION

Supplemental information can be found online at https://doi.org/10.1016/j.ymthe.2026.04.032.

REFERENCES

1. Pacesa, M., Pelea, O., and Jinek, M. (2024). Past, present, and future of CRISPR genome editing technologies. Cell *187*, 1076–1100. https://doi.org/10.1016/j.cell.2024.01.042.

2. Villiger, L., Joung, J., Koblan, L., Weissman, J., Abudayyeh, O.O., and Gootenberg, J.S. (2024). CRISPR technologies for genome, epigenome and transcriptome editing. Nat. Rev. Mol. Cell Biol. *25*, 464–487. https://doi.org/10.1038/s41580-023-00697-6.

3. Molla, K.A., and Yang, Y. (2020). Predicting CRISPR/Cas9-Induced Mutations for Precise Genome Editing. Trends Biotechnol. *38*, 136–141. https://doi.org/10.1016/j.tibtech.2019.08.002.

4. Shi, X., Shou, J., Mehryar, M.M., Li, J., Wang, L., Zhang, M., Huang, H., Sun, X., and Wu, Q. (2019). Cas9 has no exonuclease activity resulting in staggered cleavage with overhangs and predictable di- and tri-nucleotide CRISPR insertions without template donor. Cell Discov. *5*, 53. https://doi.org/10.1038/s41421-019-0120-z.

5. Nambiar, T.S., Baudrier, L., Billon, P., and Ciccia, A. (2022). CRISPR-based genome editing through the lens of DNA repair. Mol. Cell *82*, 348–388. https://doi.org/10.1016/j.molcel.2021.12.026.

6. Xue, C., and Greene, E.C. (2021). DNA Repair Pathway Choices in CRISPR-Cas9-Mediated Genome Editing. Trends Genet. *37*, 639–656. https://doi.org/10.1016/j.tig.2021.02.008.

7. Guo, T., Feng, Y.-L., Xiao, J.-J., Liu, Q., Sun, X.-N., Xiang, J.-F., Kong, N., Liu, S.-C., Chen, G.-Q., Wang, Y., et al. (2018). Harnessing accurate non-homologous end joining for efficient precise deletion in CRISPR/Cas9-mediated genome editing. Genome Biol. *19*, 170. https://doi.org/10.1186/s13059-018-1518-x.

8. Chen, W., McKenna, A., Schreiber, J., Haeussler, M., Yin, Y., Agarwal, V., Noble, W.S., and Shendure, J. (2019). Massively parallel profiling and predictive modeling of the outcomes of CRISPR/Cas9-mediated double-strand break repair. Nucleic Acids Res. *47*, 7989–8003. https://doi.org/10.1093/nar/gkz487.

9. Wang, D., Zhang, F., and Gao, G. (2020). CRISPR-Based Therapeutic Genome Editing: Strategies and In Vivo Delivery by AAV Vectors. Cell *181*, 136–150. https://doi.org/10.1016/j.cell.2020.03.023.

10. Hwang, G.-H., Lee, S.-H., Oh, M., Kim, S., Habib, O., Jang, H.-K., Kim, H.S., Kim, Y., Kim, C.H., Kim, S., and Bae, S. (2025). Large DNA deletions occur during DNA repair at 20-fold lower frequency for base editors and prime editors than for Cas9 nucleases. Nat. Biomed. Eng. *9*, 79–92. https://doi.org/10.1038/s41551-024-01277-5.

11. Schimmel, J., Muñoz-Subirana, N., Kool, H., van Schendel, R., van der Vlies, S., Kamp, J.A., de Vrij, F.M.S., Kushner, S.A., Smith, G.C.M., Boulton, S.J., and Tijsterman, M. (2023). Modulating mutational outcomes and improving precise gene editing at CRISPR-Cas9-induced breaks by chemical inhibition of end-joining pathways. Cell Rep. *42*, 112019. https://doi.org/10.1016/j.celrep.2023.112019.

12. Tatiossian, K.J., Clark, R.D.E., Huang, C., Thornton, M.E., Grubbs, B.H., and Cannon, P.M. (2021). Rational Selection of CRISPR-Cas9 Guide RNAs for Homology-Directed Genome Editing. Mol. Ther. *29*, 1057–1069. https://doi.org/10.1016/j.ymthe.2020.10.006.

13. Park, S.H., Cao, M., and Bao, G. (2023). Detection and quantification of unintended large on-target gene modifications due to CRISPR/Cas9 editing. Curr. Opin. Biomed. Eng. *28*, 100478.

14. Wen, W., and Zhang, X.-B. (2022). CRISPR-Cas9 gene editing induced complex on-target outcomes in human cells. Exp. Hematol. *110*, 13–19. https://doi.org/10.1016/j.exphem.2022.03.002.

15. Pallaseni, A., Peets, E.M., Girling, G., Crepaldi, L., Kuzmin, I., Moor, M., Muñoz-Subirana, N., Schimmel, J., Serçin, Ö., Mardin, B.R., et al. (2024). The interplay of DNA repair context with target sequence predictably biases Cas9-generated mutations. Nat. Commun. *15*, 10271. https://doi.org/10.1038/s41467-024-54566-7.

16. Shen, M.W., Arbab, M., Hsu, J.Y., Worstell, D., Culbertson, S.J., Krabbe, O., Cassa, C.A., Liu, D.R., Gifford, D.K., and Sherwood, R.I. (2018). Predictable and precise template-free CRISPR editing of pathogenic variants. Nature *563*, 646–651. https://doi.org/10.1038/s41586-018-0686-x.

17. Allen, F., Crepaldi, L., Alsinet, C., Strong, A.J., Kleshchevnikov, V., De Angeli, P., Páleníková, P., Khodak, A., Kiselev, V., Kosicki, M., et al. (2018). Predicting the mutations generated by repair of Cas9-induced double-strand breaks. Nat. Biotechnol. https://doi.org/10.1038/nbt.4317.

18. Schep, R., Brinkman, E.K., Leemans, C., Vergara, X., van der Weide, R.H., Morris, B., van Schaik, T., Manzo, S.G., Peric-Hupkes, D., van den Berg, J., et al. (2021). Impact of chromatin context on Cas9-induced DNA double-strand break repair pathway balance. Mol. Cell *81*, 2216–2230.e10. https://doi.org/10.1016/j.molcel.2021.03.032.

19. Tao, J., Bauer, D.E., and Chiarle, R. (2023). Assessing and advancing the safety of CRISPR-Cas tools: from DNA to RNA editing. Nat. Commun. *14*, 212. https://doi.org/10.1038/s41467-023-35886-6.

20. Boutin, J., Cappellen, D., Rosier, J., Amintas, S., Dabernat, S., Bedel, A., and Moreau-Gaudry, F. (2022). ON-Target Adverse Events of CRISPR-Cas9 Nuclease: More Chaotic than Expected. CRISPR J. *5*, 19–30. https://doi.org/10.1089/crispr.2021.0120.

21. Cullot, G., Aird, E.J., Schlapansky, M.F., Yeh, C.D., van de Venn, L., Vykhlyantseva, I., Kreutzer, S., Mailänder, D., Lewków, B., Klermund, J., et al. (2025). Genome editing with the HDR-enhancing DNA-PKcs inhibitor AZD7648 causes large-scale genomic alterations. Nat. Biotechnol. *43*, 1778–1782. https://doi.org/10.1038/s41587-024-02488-6.

22. Tsuchida, C.A., Brandes, N., Bueno, R., Trinidad, M., Mazumder, T., Yu, B., Hwang, B., Chang, C., Liu, J., Sun, Y., et al. (2023). Mitigation of chromosome loss in clinical CRISPR-Cas9-engineered T cells. Cell *186*, 4567–4582.e20. https://doi.org/10.1016/j.cell.2023.08.041.

23. Park, S.H., Cao, M., Pan, Y., Davis, T.H., Saxena, L., Deshmukh, H., Fu, Y., Treangen, T., Sheehan, V.A., and Bao, G. (2022). Comprehensive analysis and accurate quantification of unintended large gene modifications induced by CRISPR-Cas9 gene editing. Sci. Adv. *8*, eabo7676. https://doi.org/10.1126/sciadv.abo7676.

24. Saito, S., Maeda, R., and Adachi, N. (2017). Dual loss of human POLQ and LIG4 abolishes random integration. Nat. Commun. *8*, 16112. https://doi.org/10.1038/ncomms16112.

25. Schimmel, J., Kool, H., van Schendel, R., and Tijsterman, M. (2017). Mutational signatures of non-homologous and polymerase theta-mediated end-joining in embryonic stem cells. EMBO J. *36*, 3634–3649. https://doi.org/10.15252/embj.201796948.

26. Min, J., Zhao, J., Zagelbaum, J., Lee, J., Takahashi, S., Cummings, P., Schooley, A., Dekker, J., Gottesman, M.E., Rabadan, R., and Gautier, J. (2023). Mechanisms of insertions at a DNA double-strand break. Mol. Cell *83*, 2434–2448.e7. https://doi.org/10.1016/j.molcel.2023.06.016.

27. Chehelgerdi, M., Chehelgerdi, M., Khorramian-Ghahfarokhi, M., Shafieizadeh, M., Mahmoudi, E., Eskandari, F., Rashidi, M., Arshi, A., and Mokhtari-Farsani, A. (2024). Comprehensive review of CRISPR-based gene editing: mechanisms, challenges, and applications in cancer therapy. Mol. Cancer *23*, 9. https://doi.org/10.1186/s12943-023-01925-5.

28. Raguram, A., Banskota, S., and Liu, D.R. (2022). Therapeutic in vivo delivery of gene editing agents. Cell *185*, 2806–2827. https://doi.org/10.1016/j.cell.2022.03.045.

29. Hanlon, K.S., Kleinstiver, B.P., Garcia, S.P., Zaborowski, M.P., Volak, A., Spirig, S.E., Muller, A., Sousa, A.A., Tsai, S.Q., Bengtsson, N.E., et al. (2019). High levels of AAV vector integration into CRISPR-induced DNA breaks. Nat. Commun. *10*, 4439. https://doi.org/10.1038/s41467-019-12449-2.

30. Nelson, C.E., Wu, Y., Gemberling, M.P., Oliver, M.L., Waller, M.A., Bohning, J.D., Robinson-Hamm, J.N., Bulaklak, K., Castellanos Rivera, R.M., Collier, J.H., et al. (2019). Long-term evaluation of AAV-CRISPR genome editing for Duchenne muscular dystrophy. Nat. Med. *25*, 427–432. https://doi.org/10.1038/s41591-019-0344-3.

31. He, L., Ding, Y., Zhao, Y., So, K.K., Peng, X.L., Li, Y., Yuan, J., He, Z., Chen, X., Sun, H., and Wang, H. (2021). CRISPR/Cas9/AAV9-mediated in vivo editing identifies MYC regulation of 3D genome in skeletal muscle stem cell. Stem Cell Rep. *16*, 2442–2458. https://doi.org/10.1016/j.stemcr.2021.08.011.

32. He, L., He, Z., Li, Y., Sun, H., and Wang, H. (2023). In Vivo Investigation of Gene Function in Muscle Stem Cells by CRISPR/Cas9-Mediated Genome Editing. Methods Mol. Biol. *2640*, 287–311. https://doi.org/10.1007/978-1-0716-3036-5_21.

33. Zhao, Y., Zhou, J., He, L., Li, Y., Yuan, J., Sun, K., Chen, X., Bao, X., Esteban, M.A., Sun, H., and Wang, H. (2019). MyoD induced enhancer RNA interacts with hnRNPL to activate target gene transcription during myogenic differentiation. Nat. Commun. *10*, 5787. https://doi.org/10.1038/s41467-019-13598-0.

34. Zhao, Y., Ding, Y., He, L., Zhou, Q., Chen, X., Li, Y., Alfonsi, M.V., Wu, Z., Sun, H., and Wang, H. (2023). Multiscale 3D genome reorganization during skeletal muscle stem cell lineage progression and aging. Sci. Adv. *9*, eabo1360. https://doi.org/10.1126/sciadv.abo1360.

35. Zhang, S., Yang, F., Huang, Y., He, L., Li, Y., Wan, Y.C.E., Ding, Y., Chan, K.M., Xie, T., Sun, H., and Wang, H. (2023). ATF3 induction prevents precocious activation of skeletal muscle stem cell by regulating H2B expression. Nat. Commun. *14*, 4978. https://doi.org/10.1038/s41467-023-40465-w.

36. Liu, M., Zhang, W., Xin, C., Yin, J., Shang, Y., Ai, C., Li, J., Meng, F.-L., and Hu, J. (2021). Global detection of DNA repair outcomes induced by CRISPR-Cas9. Nucleic Acids Res. *49*, 8732–8742. https://doi.org/10.1093/nar/gkab686.

37. Chakrabarti, A.M., Henser-Brownhill, T., Monserrat, J., Poetsch, A.R., Luscombe, N.M., and Scaffidi, P. (2019). Target-Specific Precision of CRISPR-Mediated Genome Editing. Mol. Cell *73*, 699–713.e6. https://doi.org/10.1016/j.molcel.2018.11.031.

38. van Overbeek, M., Capurso, D., Carter, M.M., Thompson, M.S., Frias, E., Russ, C., Reece-Hoyes, J.S., Nye, C., Gradia, S., Vidal, B., et al. (2016). DNA Repair Profiling Reveals Nonrandom Outcomes at Cas9-Mediated Breaks. Mol. Cell *63*, 633–646. https://doi.org/10.1016/j.molcel.2016.06.037.

39. Longo, G.M.C., Sayols, S., Kotini, A.G., Heinen, S., Möckel, M.M., Beli, P., and Roukos, V. (2025). Linking CRISPR-Cas9 double-strand break profiles to gene editing precision with BreakTag. Nat. Biotechnol. *43*, 608–622. https://doi.org/10.1038/s41587-024-02238-8.

40. Shou, J., Li, J., Liu, Y., and Wu, Q. (2018). Precise and Predictable CRISPR Chromosomal Rearrangements Reveal Principles of Cas9-Mediated Nucleotide Insertion. Mol. Cell *71*, 498–509.e4. https://doi.org/10.1016/j.molcel.2018.06.021.

41. Sebastian, R., Sun, E.G., Fedkenheuer, M., Fu, H., Jung, S., Thakur, B.L., Redon, C.E., Pegoraro, G., Tran, A.D., Gross, J.M., et al. (2025). Mechanism for local attenuation of DNA replication at double-strand breaks. Nature *639*, 1084–1092. https://doi.org/10.1038/s41586-024-08557-9.

42. Wang, R., Chen, F., Chen, Q., Wan, X., Shi, M., Chen, A.K., Ma, Z., Li, G., Wang, M., Ying, Y., et al. (2022). MyoD is a 3D genome structure organizer for muscle cell identity. Nat. Commun. *13*, 205. https://doi.org/10.1038/s41467-021-27865-6.

43. Bi, C., Wang, L., Yuan, B., Zhou, X., Li, Y., Wang, S., Pang, Y., Gao, X., Huang, Y., and Li, M. (2020). Long-read individual-molecule sequencing reveals CRISPR-induced genetic heterogeneity in human ESCs. Genome Biol. *21*, 213. https://doi.org/10.1186/s13059-020-02143-8.

44. Bi, C., Yuan, B., Zhang, Y., Wang, M., Tian, Y., and Li, M. (2025). Prevalent integration of genomic repetitive and regulatory elements and donor sequences at CRISPR-Cas9-induced breaks. Commun. Biol. *8*, 94. https://doi.org/10.1038/s42003-025-07539-5.

45. Simpson, B.P., Yrigollen, C.M., Izda, A., and Davidson, B.L. (2023). Targeted long-read sequencing captures CRISPR editing and AAV integration outcomes in brain. Mol. Ther. *31*, 760–773. https://doi.org/10.1016/j.ymthe.2023.01.004.

46. Wimberger, S., Akrap, N., Firth, M., Brengdahl, J., Engberg, S., Schwinn, M.K., Slater, M.R., Lundin, A., Hsieh, P.-P., Li, S., et al. (2023). Simultaneous inhibition of DNA-PK and PolΘ improves integration efficiency and precision of genome editing. Nat. Commun. *14*, 4761. https://doi.org/10.1038/s41467-023-40344-4.

47. Schlapansky, M.F., Schröder, M.S., Santinha, A.J., Rothgangl, T., Ioannidi, E.I., Cullot, G., Lewkow, B., Ortega, G.C., Nouraiz, A., Mailänder, D., et al. (2025). Cell-stereotyped DNA repair outcomes are widespread during genome editing. Preprint at bioRxiv. https://doi.org/10.1101/2025.10.23.684114.

48. Thürkauf, M., Lin, S., Oliveri, F., Grimm, D., Platt, R.J., and Rüegg, M.A. (2023). Fast, multiplexable and efficient somatic gene deletions in adult mouse skeletal muscle fibers using AAV-CRISPR/Cas9. Nat. Commun. *14*, 6116. https://doi.org/10.1038/s41467-023-41769-7.

49. Liu, L., Cheung, T.H., Charville, G.W., and Rando, T.A. (2015). Isolation of skeletal muscle stem cells by fluorescence-activated cell sorting. Nat. Protoc. *10*, 1612–1624. https://doi.org/10.1038/nprot.2015.110.

50. Concordet, J.-P., and Haeussler, M. (2018). CRISPOR: intuitive guide selection for CRISPR/Cas9 genome editing experiments and screens. Nucleic Acids Res. *46*, W242–W245. https://doi.org/10.1093/nar/gky354.

51. Li, Y., Li, C., Sun, Q., Liu, X., Chen, F., Cheung, Y., Zhao, Y., Xie, T., Chazaud, B., Sun, H., and Wang, H. (2025). Skeletal muscle stem cells modulate niche function in Duchenne muscular dystrophy mouse through YY1-CCL5 axis. Nat. Commun. *16*, 1324. https://doi.org/10.1038/s41467-025-56474-w.

52. Qiao, Y., Sun, Q., Chen, X., He, L., Wang, D., Su, R., Xue, Y., Sun, H., and Wang, H. (2023). Nuclear m6A reader YTHDC1 promotes muscle stem cell activation/proliferation by regulating mRNA splicing and nuclear export. Elife *12*, e82703. https://doi.org/10.7554/eLife.82703.

53. Bolger, A.M., Lohse, M., and Usadel, B. (2014). Trimmomatic: a flexible trimmer for Illumina sequence data. Bioinformatics *30*, 2114–2120. https://doi.org/10.1093/bioinformatics/btu170.

54. van Schendel, R., Schimmel, J., and Tijsterman, M. (2022). SIQ: easy quantitative measurement of mutation profiles in sequencing data. NAR Genom. Bioinform. *4*, lqac063. https://doi.org/10.1093/nargab/lqac063.

55. Hao, Z., Lv, D., Ge, Y., Shi, J., Weijers, D., Yu, G., and Chen, J. (2020). RIdeogram: drawing SVG graphics to visualize and map genome-wide data on the idiograms. Peerj. Comput. Sci. *6*, e251. https://doi.org/10.7717/peerj-cs.251.

56. Li, H. (2013). Aligning sequence reads, clone sequences and assembly contigs with BWA-MEM. Preprint at arXiv. https://doi.org/10.48550/arXiv.1303.3997.

57. Danecek, P., Bonfield, J.K., Liddle, J., Marshall, J., Ohan, V., Pollard, M.O., Whitwham, A., Keane, T., McCarthy, S.A., Davies, R.M., and Li, H. (2021).

Twelve years of SAMtools and BCFtools. Gigascience *10*, giab008. https://doi.org/10.1093/gigascience/giab008.

58. Tarasov, A., Vilella, A.J., Cuppen, E., Nijman, I.J., and Prins, P. (2015). Sambamba: fast processing of NGS alignment formats. Bioinformatics *31*, 2032–2034. https://doi.org/10.1093/bioinformatics/btv098.

59. Amemiya, H.M., Kundaje, A., and Boyle, A.P. (2019). The ENCODE Blacklist: Identification of Problematic Regions of the Genome. Sci. Rep. *9*, 9354. https://doi.org/10.1038/s41598-019-45839-z.

60. Quinlan, A.R., and Hall, I.M. (2010). BEDTools: a flexible suite of utilities for comparing genomic features. Bioinformatics *26*, 841–842. https://doi.org/10.1093/bioinformatics/btq033.

61. Ramírez, F., Ryan, D.P., Grüning, B., Bhardwaj, V., Kilpert, F., Richter, A.S., Heyne, S., Dündar, F., and Manke, T. (2016). deepTools2: a next generation web server for deep-sequencing data analysis. Nucleic Acids Res. *44*, W160–W165. https://doi.org/10.1093/nar/gkw257.

62. Zhang, Y., Liu, T., Meyer, C.A., Eeckhoute, J., Johnson, D.S., Bernstein, B.E., Nusbaum, C., Myers, R.M., Brown, M., Li, W., and Liu, X.S. (2008). Model-based analysis of ChIP-Seq (MACS). Genome Biol. *9*, R137. https://doi.org/10.1186/gb-2008-9-9-r137.

63. Kent, W.J., Zweig, A.S., Barber, G., Hinrichs, A.S., and Karolchik, D. (2010). BigWig and BigBed: enabling browsing of large distributed datasets. Bioinformatics *26*, 2204–2207. https://doi.org/10.1093/bioinformatics/btq351.

64. Bushnell, B. (2014). BBMap: A Fast, Accurate, Splice-Aware Aligner. In Conference: 9th Annual Genomics of Energy & Environment Meeting (SourceForge).

65. Wang, Q., Li, M., Wu, T., Zhan, L., Li, L., Chen, M., Xie, W., Xie, Z., Hu, E., Xu, S., and Yu, G. (2022). Exploring Epigenomic Datasets by ChIPseeker. Curr. Protoc. *2*, e585. https://doi.org/10.1002/cpz1.585.

66. Yu, G., Wang, L.-G., and He, Q.-Y. (2015). ChIPseeker: an R/Bioconductor package for ChIP peak annotation, comparison and visualization. Bioinformatics *31*, 2382–2383. https://doi.org/10.1093/bioinformatics/btv145.

67. Durand, N.C., Shamim, M.S., Machol, I., Rao, S.S.P., Huntley, M.H., Lander, E.S., and Aiden, E.L. (2016). Juicer Provides a One-Click System for Analyzing Loop-Resolution Hi-C Experiments. Cell Syst. *3*, 95–98. https://doi.org/10.1016/j.cels.2016.07.002.

68. Hinrichs, A.S., Karolchik, D., Baertsch, R., Barber, G.P., Bejerano, G., Clawson, H., Diekhans, M., Furey, T.S., Harte, R.A., Hsu, F., et al. (2006). The UCSC Genome Browser Database: update 2006. Nucleic Acids Res. *34*, D590–D598. https://doi.org/10.1093/nar/gkj144.

YMTHE, Volume *34*

Supplemental Information

In vivo systematic detection of the outcomes of CRISPR-Cas9-mediated DNA repair in skeletal muscle stem cells

Liangqiang He, Yang Fu, Ziliu Wang, Qin Zhou, Hao Sun, and Huating Wang

Supplemental Material

List of Supplemental Tables

Table S1. List of single, dual sgRNA, IDM-Seq, and Endo-LI donor sites.

Table S2. SIQ run information.

Table S3. SIQ outcomes for single-sgRNA sites.

Table S4. Jaccard similarity scores between target sites.

Table S5. SIQ outcomes for dual-sgRNA sites.

Table S6. SIQ outcomes for endo-LI donor samples.

Table S7. IDM-Seq indel profiles and statistics.

Table S8. SIQ outcomes for AAV dose groups.

Table S9. SIQ outcomes for *in vivo* perturbation.

Table S10. Sequences of oligos used in this study.

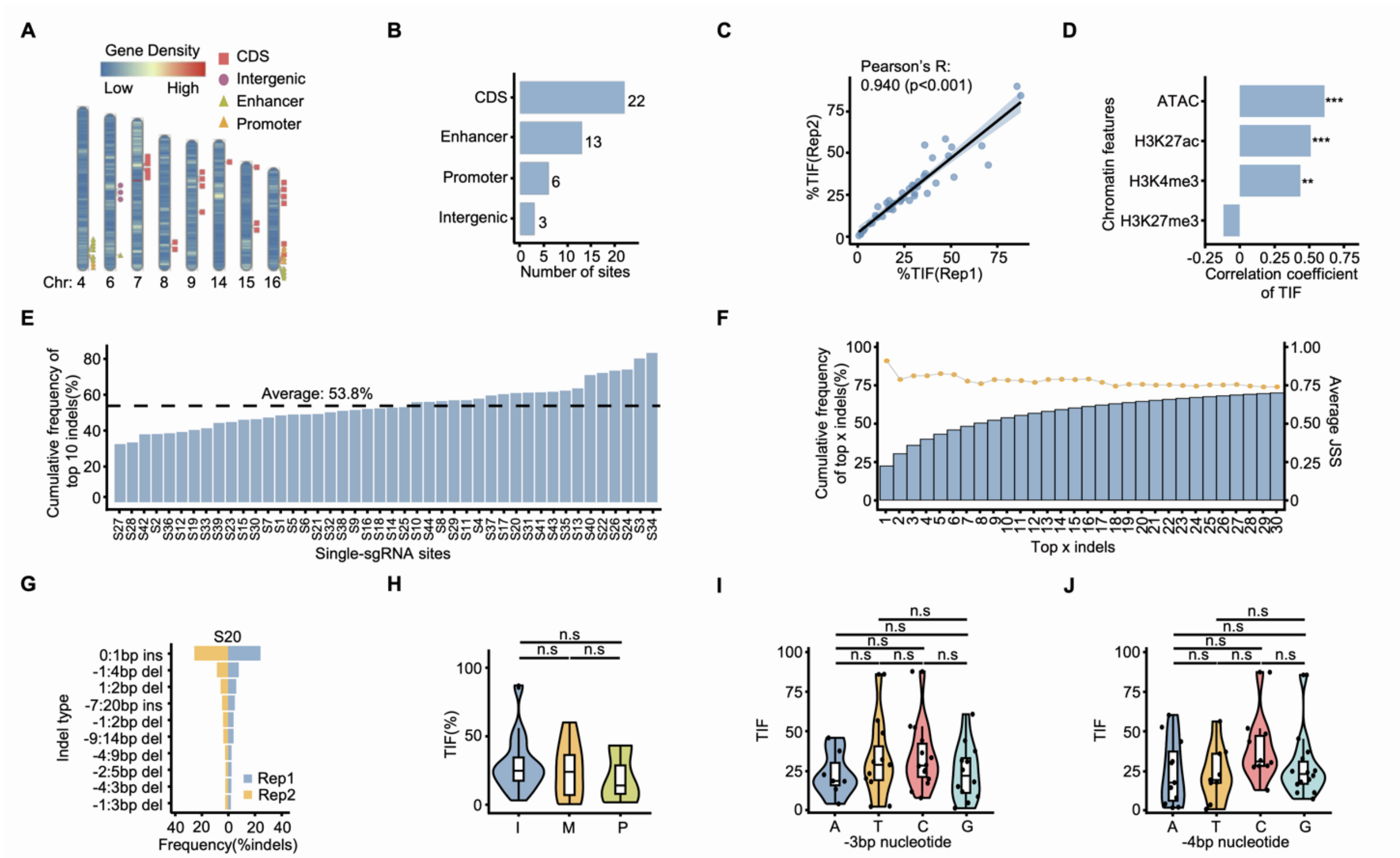

Figure S1. *In vivo* mapping of CRISPR/Cas9 induced indel profiles in MuSCs using single-sgRNAs. (**A**) Distribution of the 44 single-sgRNA targets across chromosomes. Genomic annotation of each target is shown. (**B**) The number of target sites with the indicated genomic annotations. (**C**) Correlation of TIF between two biological replicates across all 44 targets. (**D**) Spearman's correlation between TIF and indicated chromatin features. (**E**) The cumulative indel frequency of the top ten most frequent indels for each target. The average frequency across all sites is shown by the dash line. (**F**) The cumulative indel frequency of the top 1 to 30 most frequent indels across 44 targets, along with the corresponding average JSS of paired replicates across all targets. (**G**) Frequency of the top ten indel types in each replicate of S20. The y-axis displays the indel identity (start coordinate relative to the breakpoint): (indel size and type). (**H**) The TIF in I, M and P groups, respectively. (**I-J**) The TIF at sites with A, T, C, G at -3 bp (**I**) or -4 bp (**J**) upstream of the PAM motif. Statistical significance for (**H**) - (**J**) was calculated using a t-test. ** $p < 0.01$, ***$p < 0.001$ and ns, no significance.

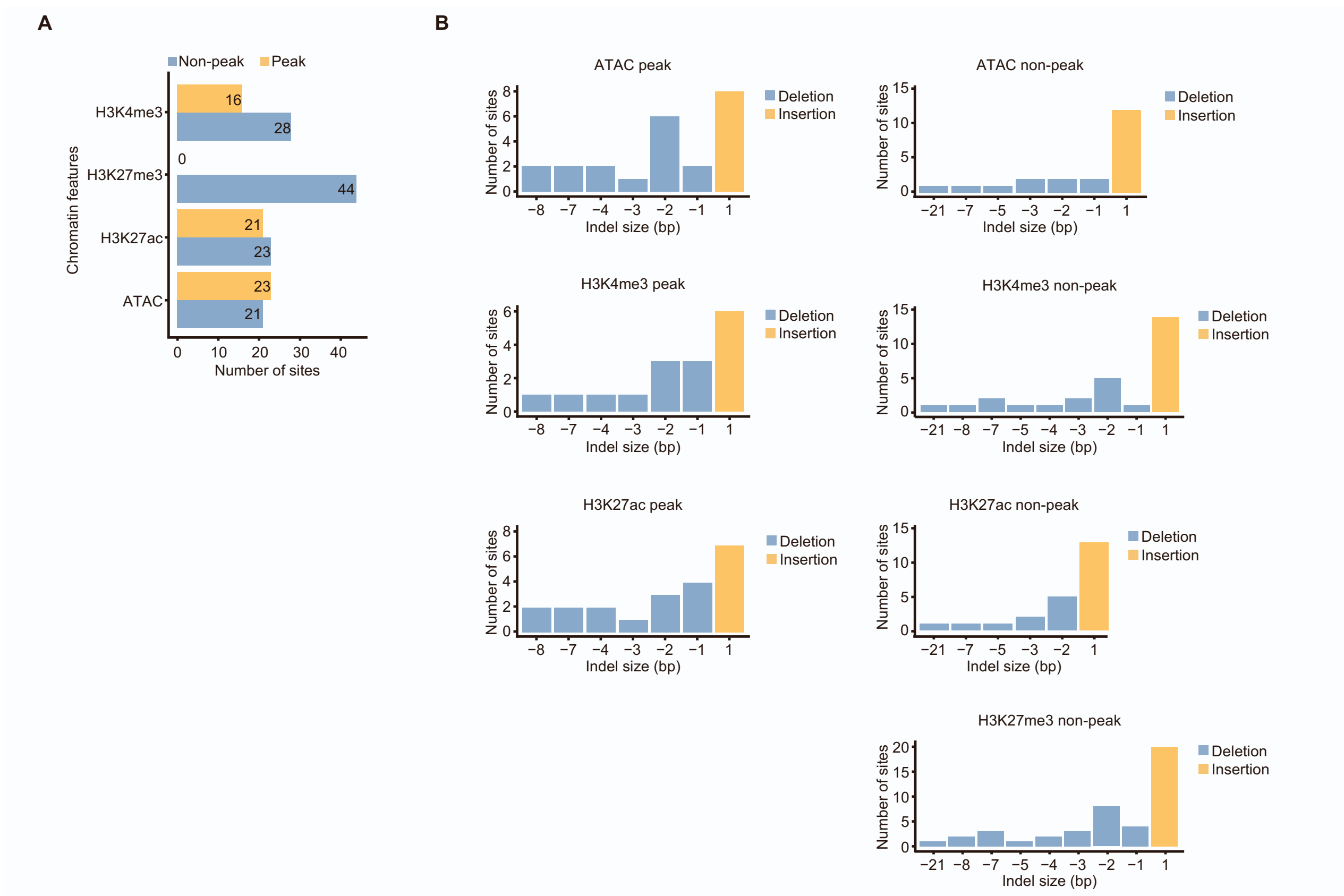

Figure S2. Impact of chromatin states on editing precision. (**A**) Numbers of sites located in peak or non-peak regions of each indicated chromatin feature. (**B**) The number of sites with the indicated type of top 1 indels in peak or non-peak regions of each indicated chromatin feature.

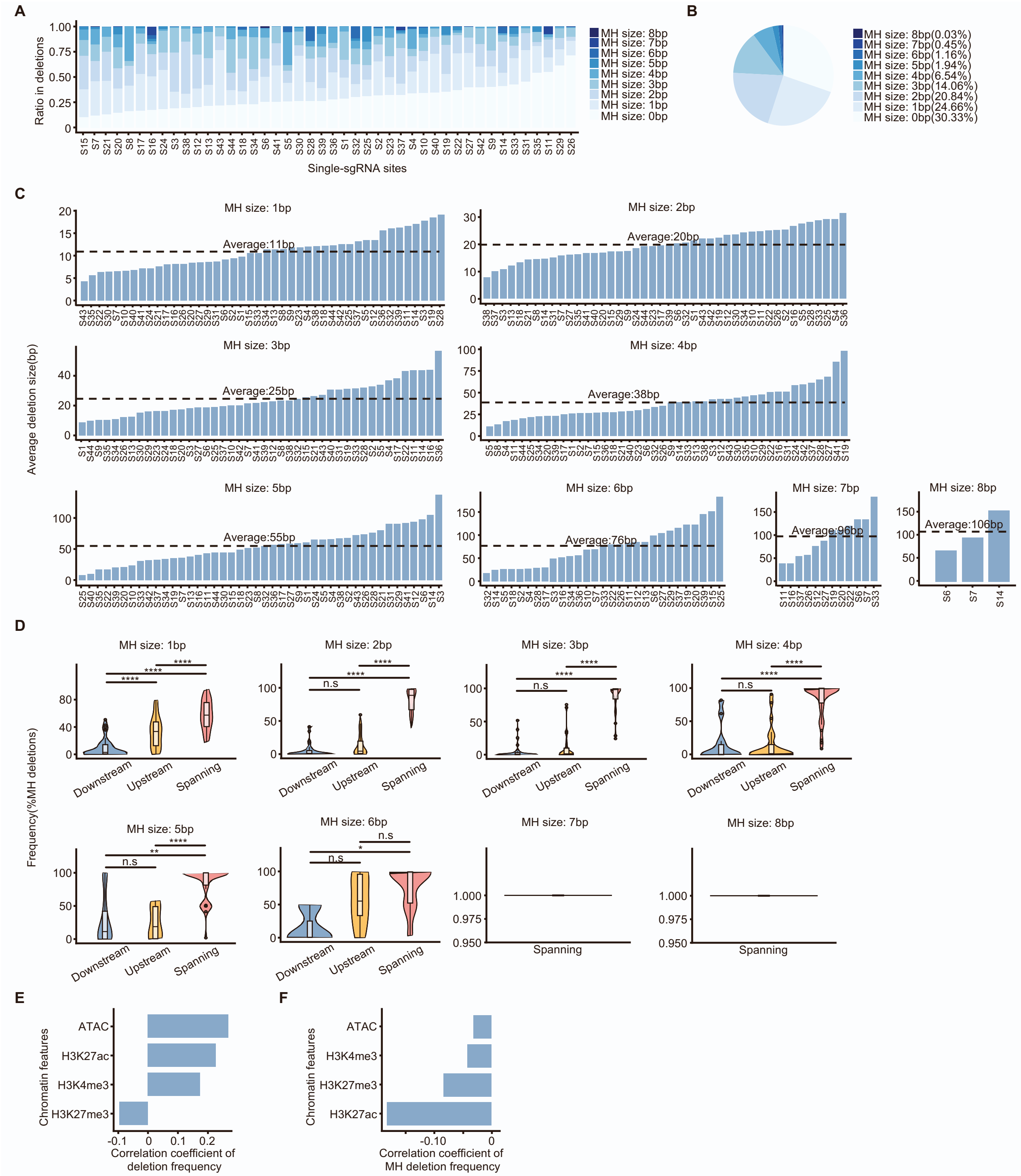

Figure S3. Extensive use of microhomology in CRISPR/Cas9 induced deletions in MuSCs *in vivo*. (**A**) Ratio of deletions with MH of varying sizes at each site. (**B**) Pie chart showing the average frequency of deletions with MH of varying sizes across all sites. (**C**) The average deletion sizes categorized by varying

MH lengths. (**D**) Violin plots illustrating the frequency of deletions with varying MH sizes categorized by direction. Statistical significance was assessed using a t-test. *$p < 0.05$, ** $p < 0.01$, ****$p < 0.0001$ and ns, no significance. (**E**-**F**) Spearman's correlation between deletion frequency (**E**) or MH frequency in deletions (**F**) and indicated chromatin features.

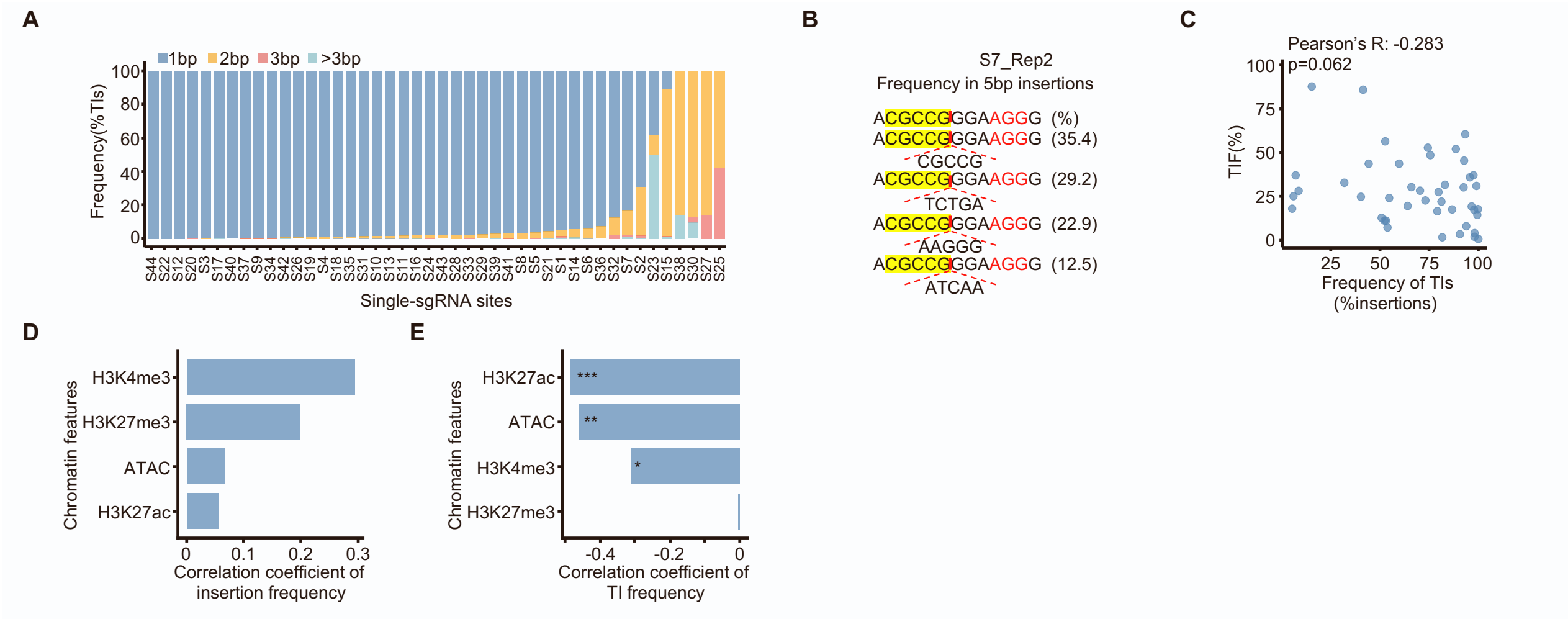

Figure S4. CRISPR/Cas9-mediated short insertions in MuSCs *in vivo* are templated and predictable. (**A**) Frequency of TIs with varying sizes in all TIs for each target site. (**B**) An example of > 3 bp TIs on S7. The nucleotides at -8 bp to -4 bp upstream of the PAM motif are highlighted in yellow. The frequency is presented as percentage in 5 bp insertions. (**C**) Correlation between TIF and TI frequency in all insertions. (**D**-**E**) Spearman's correlation between insertion frequency (**D**) or TI frequency (**E**) and indicated chromatin features. *p < 0.05, ** p < 0.01, ***p < 0.001.

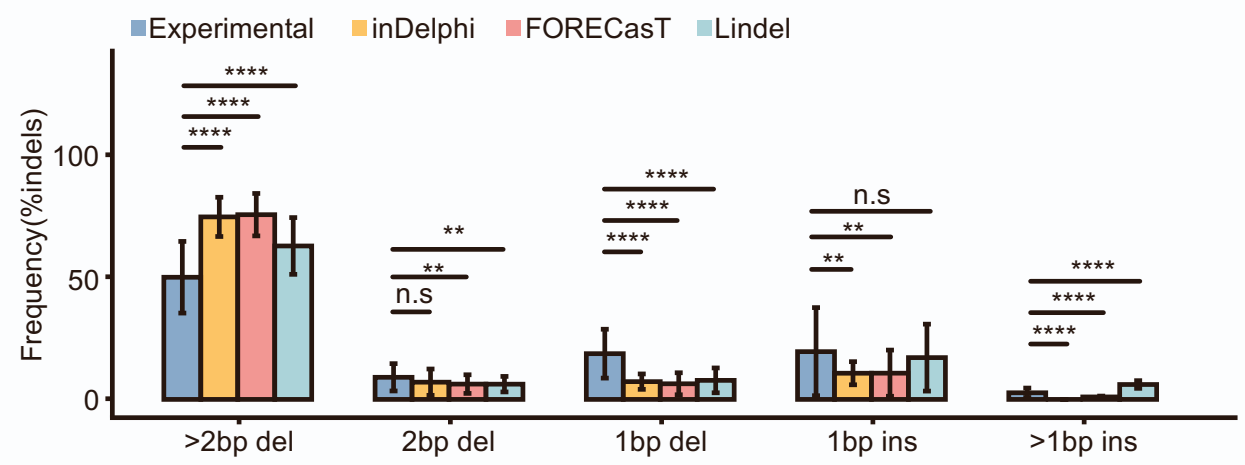

Figure S5. Limited predicting power of the machine learning models *in vivo*. Comparison between the frequency in total indels predicted by inDelphi, FORECasT, and Lindel and the experimentally measured for > 2 bp deletions, 2 bp deletions, 1 bp deletions, 1 bp insertions and > 1 bp insertions across the 44 sites, respectively. Statistical significance was calculated using a t-test. ** $p < 0.01$, ****$p < 0.0001$ and ns, no significance.

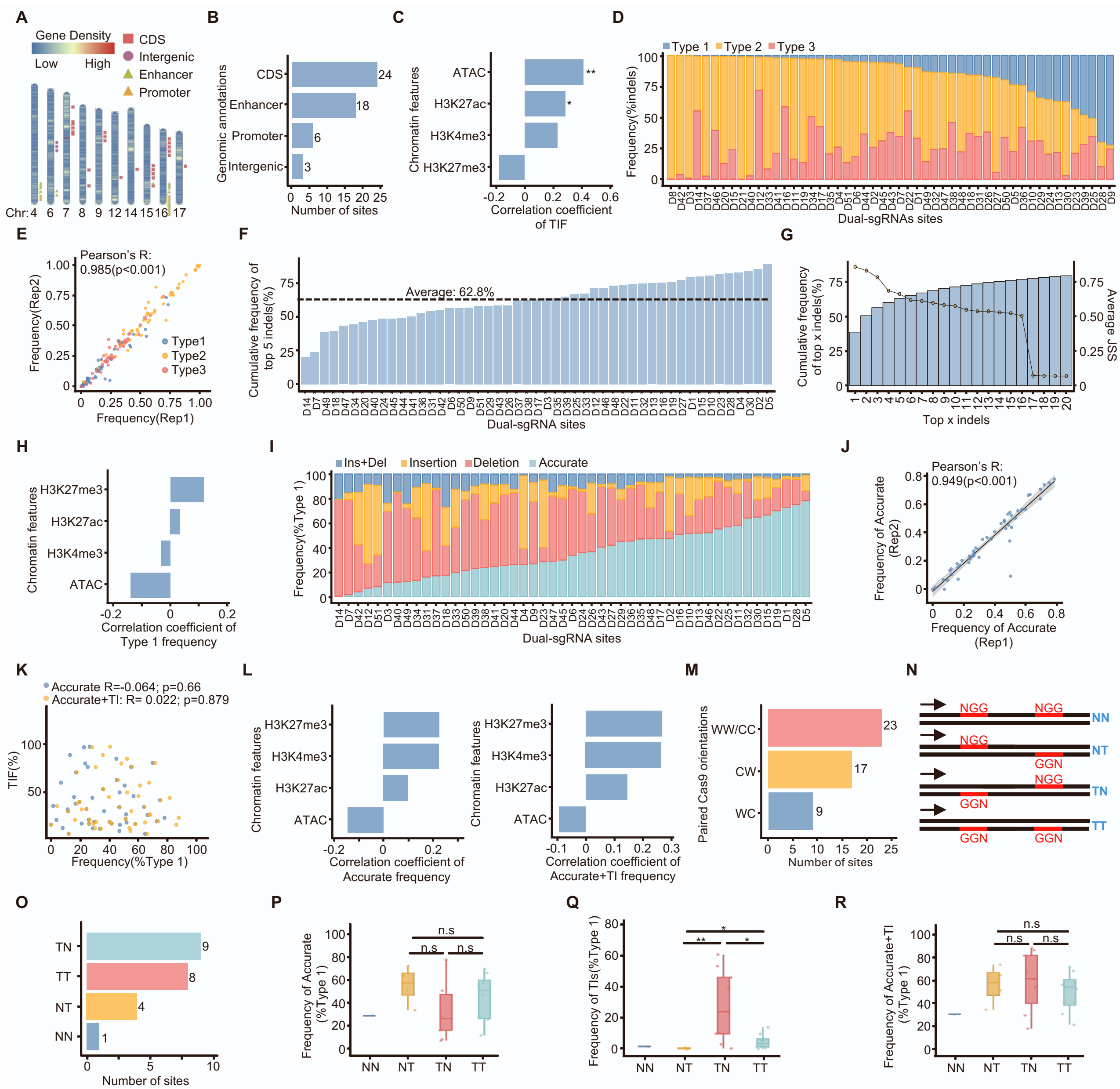

Figure S6. *In vivo* mapping of CRISPR/Cas9 induced indel profiles in MuSCs using dual-sgRNAs. (**A**) Distribution of the 51 dual-sgRNA targets across chromosomes. Genomic annotation of each target is shown. (**B**) The number of target sites with the indicated genomic annotations. (**C**) Spearman's correlation between TIF and indicated chromatin features. (**D**) Frequency of Type 1, Type2 and Type 3 indels for each target. (**E**) Correlation of Type 1, Type 2 and Type 3 indel frequency between two biological replicates across all 51 targets. (**F**) The cumulative indel frequency of the top five most frequent indels for each site. The average cumulative indel frequency across all sites is shown. (**G**) The cumulative indel frequency of

the top 1 to 20 most frequent indels across 51 targets, along with the corresponding average JSS of paired replicates across all targets. (**H**) Spearman's correlation between Type 1 frequency and indicated chromatin features. (**I**) Frequency of different subtypes of indel within Type 1 group for each target. 49 targets with over 100 Type 1 events were included for subtype analysis. (**J**) Pearson's correlation of the frequency of accurate ligation (Accurate) in Type 1 events between two biological replicates across targets. (**K**) Correlation of TIF with the frequency of Accurate (blue) or Accurate + TI (orange) in Type 1 indels. (**L**) Spearman's correlation between the frequency of Accurate (Left) or Accurate + TI (Right) in Type 1 indels and indicated chromatin features. (**M**) Numbers of target sites categorized by WW/CC, CW and WC PAM orientation. (**N**) Schematic showing four Cas9 PAM orientations for dual sgRNAs related to the transcription direction. TT, TN, NT, and NN orientations are determined by the recognition of Cas9 to the PAM motif on either the transcribed (T) or non-transcribed strand (N). Black arrows indicate the direction of transcription. (**O**) Numbers of target sites categorized by TT, TN, NT, and NN orientation. (**P**-**R**) Boxplot illustrating the frequency of Accurate (**P**), TIs (**Q**), Accurate + TI (**R**) in total Type 1 on targets with TT, TN, NT, and NN orientation. Statistical significance was calculated using a t-test for categories with $n \geq 3$ in (**P**) - (**R**). *$p < 0.05$, ** $p < 0.01$ and ns, no significance.

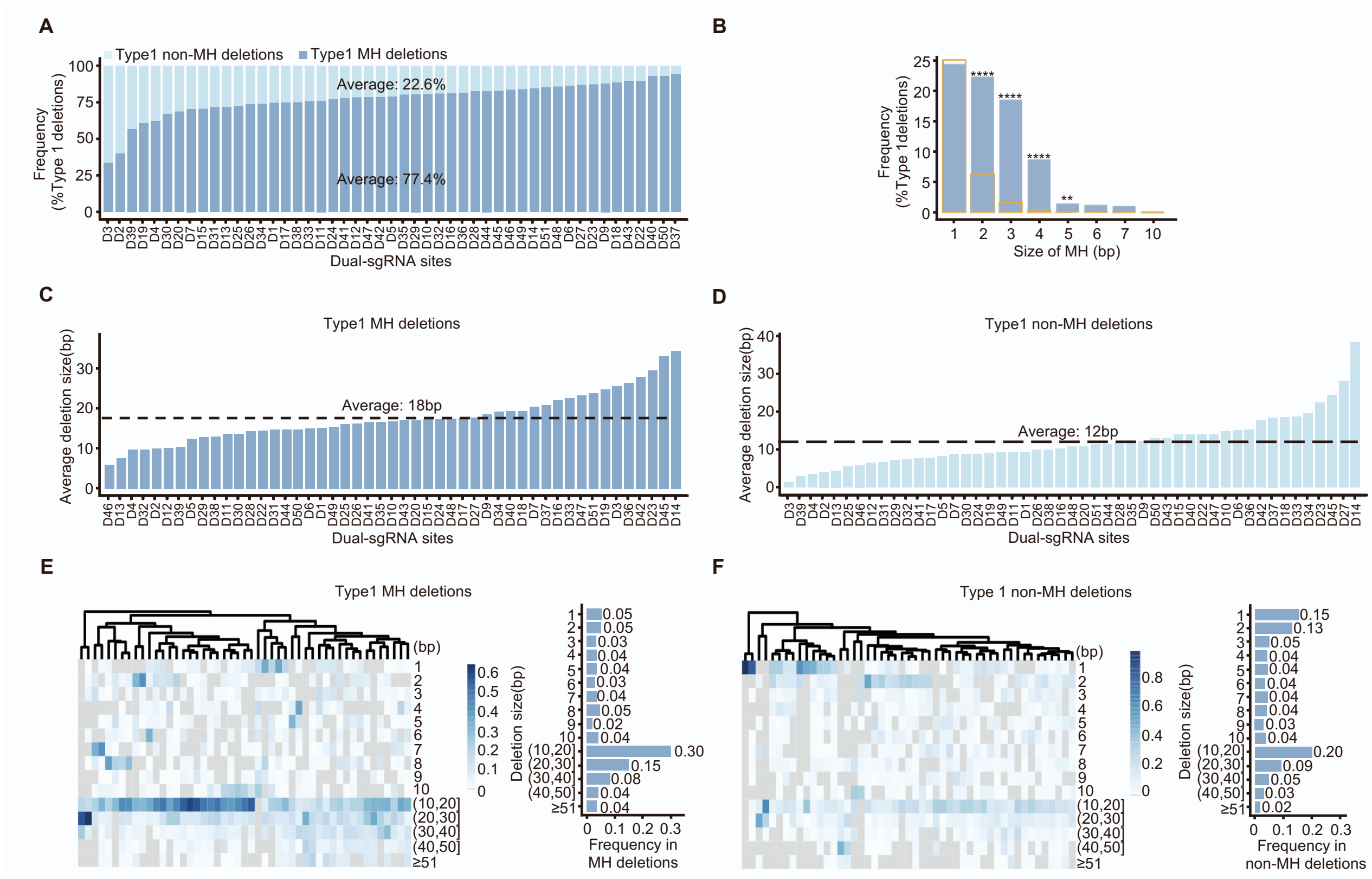

Figure S7. Extensive use of microhomology in dual sgRNAs induced deletion in MuSCs *in vivo*. (**A**) Frequency of MH and non-MH mediated deletions in Type 1 deletions for each dual-sgRNA target. The average frequency of MH and non-MH mediated deletions across all sites is shown. (**B**) Frequency of deletions with MH of varying sizes in Type 1 deletions. The orange bar represents the expected frequency. (**C**-**D**) Average deletion size for MH (**C**) and non-MH (**D**) mediated deletions for each site. The average deletion size for MH and non-MH mediated deletions across all dual-sgRNA sites is shown by dash line. (**E**) Left: Heatmaps illustrating the frequency of MH mediated deletions categorized by size across dual-sgRNA targets. The targets are clustered using the "complete" hierarchical clustering. Right: The average frequency across all targets for each category with varying deletion size in MH mediated deletions. (**F**) The above analysis was conducted in non-MH mediated deletions. Statistical significance was calculated using a one-tailed t-test for (**B**). ** $p < 0.01$, ****$p < 0.0001$.

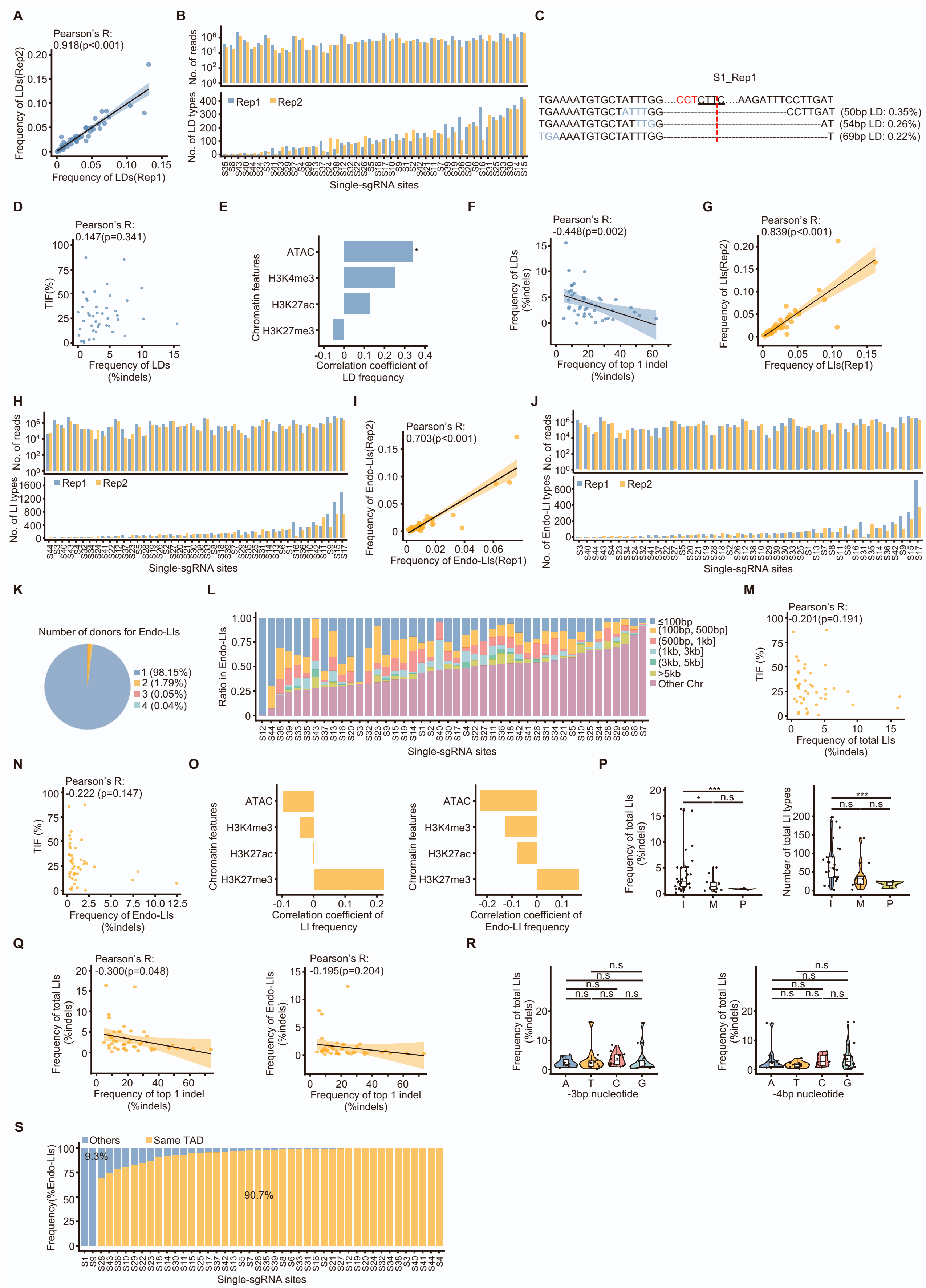

A
Pearson's R:
0.918(p<0.001)
Frequency of LDs(Rep2)
Frequency of LDs(Rep1)
B
No. of reads
No. of LD types
Rep1
Rep2
Single-sgRNA sites
C
S1_Rep1
TGAAAATGTGCTATTTGG....CCTCTTC....AAGATTTCCTTGAT
TGAAAATGTGCTATTTGG-----CCTTGAT (50bp LD: 0.35%)
TGAAAATGTGCTATTTG-----AT (54bp LD: 0.26%)
TGAAAATGTGCTATTTGG-----T (69bp LD: 0.22%)
D
Pearson's R:
0.147(p=0.341)
TIF(%)
Frequency of LDs
(%indels)
E
ATAC
H3K4me3
H3K27ac
H3K27me3
Chromatin features
Correlation coefficient of
LD frequency
F
Pearson's R:
-0.448(p=0.002)
Frequency of LDs
(%indels)
Frequency of top 1 indel
(%indels)
G
Pearson's R:
0.839(p<0.001)
Frequency of LIs(Rep2)
Frequency of LIs(Rep1)
H
No. of reads
No. of LI types
Rep1
Rep2
Single-sgRNA sites
I
Pearson's R:
0.703(p<0.001)
Frequency of Endo-LIs(Rep2)
Frequency of Endo-LIs(Rep1)
J
No. of reads
No. of Endo-LI types
Rep1
Rep2
Single-sgRNA sites
K
Number of donors for Endo-LIs
1 (98.15%)
2 (1.79%)
3 (0.05%)
4 (0.04%)
L
Ratio in Endo-LIs
≤100bp
(100bp, 500bp]
(500bp, 1kb]
(1kb, 3kb]
(3kb, 5kb]
>5kb
Other Chr
Single-sgRNA sites
M
Pearson's R:
-0.201(p=0.191)
TIF (%)
Frequency of total LIs
(%indels)
N
Pearson's R:
-0.222 (p=0.147)
TIF (%)
Frequency of Endo-LIs
(%indels)
O
Chromatin features
Correlation coefficient of
LI frequency
Correlation coefficient of
Endo-LI frequency
P
Frequency of total LIs
(%indels)
Number of total LI types
Q
Pearson's R:
-0.300(p=0.048)
Frequency of total LIs
(%indels)
Frequency of top 1 indel
(%indels)
Pearson's R:
-0.195(p=0.204)
Frequency of Endo-LIs
(%indels)
R
Frequency of total LIs
(%indels)
-3bp nucleotide
-4bp nucleotide
S
Others
Same TAD
9.3%
90.7%
Frequency(%Endo-LIs)
Single-sgRNA sites

Figure S8. Analysis of CRISPR/Cas9 induced large on-target genomic modifications in MuSCs *in vivo*. (**A**) Pearson's correlation of LD frequency in total indels between two biological replicates across single-sgRNA targets. (**B**) Number of LD types defined by their start sites and the deleted sequences in each biological replicate across all targets (Lower) and number of sequencing reads for each sample (Upper). (**C**) An example showing the frequency of LDs with MH on S1. The MH tracks are marked as blue. (**D**) Pearson's correlation between TIF and LD frequency. (**E**) Spearman's correlation between LD frequency in total indels and indicated chromatin features. (**F**) Pearson's correlation between LD frequency in all indels and editing precision determined by the frequency of top 1 indel. (**G**) Pearson's correlation of LI frequency in total indels between two biological replicates across single-sgRNA targets. (**H**) Number of LI types defined by their start sites and the inserted sequences in each biological replicate across all targets (Lower) and number of sequencing reads for each sample (Upper). (**I**) Pearson's correlation of Endo-LI frequency between two biological replicates across single-sgRNA targets. (**J**) Number of Endo-LI types defined by their start sites and the inserted sequences in each biological replicate across all targets (Lower) and number of sequencing reads for each sample (Upper). (**K**) Pie charting showing the number distribution of donors for Endo-LIs. (**L**) Distance distribution between insertion donors for Endo-LIs and associated DSBs for each site. (**M**-**N**) Pearson's correlation of TIF with total LI (**M**) and Endo-LI (**N**) frequency. (**O**) Spearman's correlation between the frequency of total LIs (Left) or Endo-LIs (Right) and indicated chromatin features. (**P**) Violin plot illustrating the frequency of total LIs (Left) and number of total LI types (Right) in I, M, P groups. (**Q**) Pearson's correlation between the frequency of total LI (Left) or Endo-LI (Right) and editing precision determined by the frequency of top 1 indel. (**R**) Frequency of total LIs at sites with A, T, C, G at -3 bp (Left) of -4 bp (Right) upstream of the PAM motif. (**S**) Frequency of donor sequences originating from the DSB-containing TADs or other loci on the same chromosome for each site. Statistical significance was calculated using a t-test for (**P**) and (**R**). $^{*}p < 0.05$, $^{***}p < 0.001$ and ns, no significance.

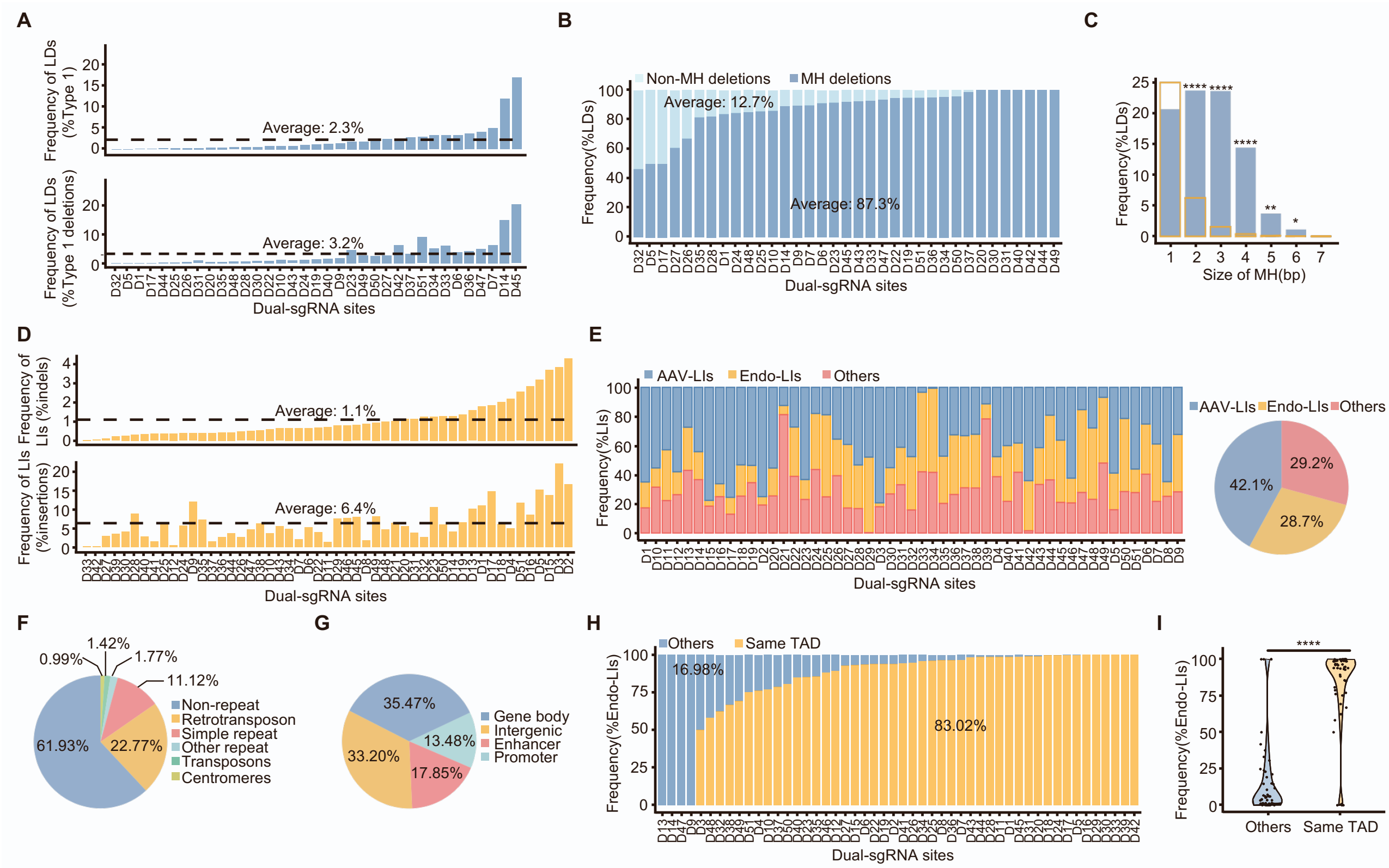

Figure S9. Analysis of CRISPR/Cas9 induced large on-target genomic modifications induced by dual-sgRNAs in MuSCs *in vivo*. (**A**) Frequency of ≥ 50 bp LDs for each dual-sgRNA site, calculated as percentage in total Type 1 events (Upper) and total Type 1 deletions (Lower). The average frequency of LDs across all sites is shown by dash line. (**B**) Frequency of MH and non-MH mediated LDs for each dual-sgRNA target. The average frequency of MH and non-MH mediated LDs across all sites is shown. (**C**) Frequency of LDs with MH of varying sizes. The orange bar represents the expected frequency for each MH size. (**D**) Frequency of ≥ 20 bp LIs for each dual-sgRNA site, calculated as percentage in total indels (Upper) and total insertions (Lower). The average frequency of LIs is shown by dash line. (**E**) Left: Frequency of different types of LIs for each dual-sgRNA target. Right: Pie chart showing the average frequency of each LI type across all sites. (**F**) Pie chart showing the frequency of donor sequence categorized by types of repetitive elements for Endo-LIs. (**G**) Pie chart showing the frequency of donor sequence categorized by genomic annotations for Endo-LIs. (**H**) Frequency of donor sequences originating from the DSB-containing TADs or other loci on the same chromosome for each dual-sgRNA site. (**I**) Violin

plot showing significantly higher frequency of donor sequences originating from DSB-containing TADs compared to other loci on the same chromosome. A one-tail t-test was used for (**C**) and a paired t-test was used for (**I**). $*p < 0.05$, $** p < 0.01$, $****p < 0.0001$.

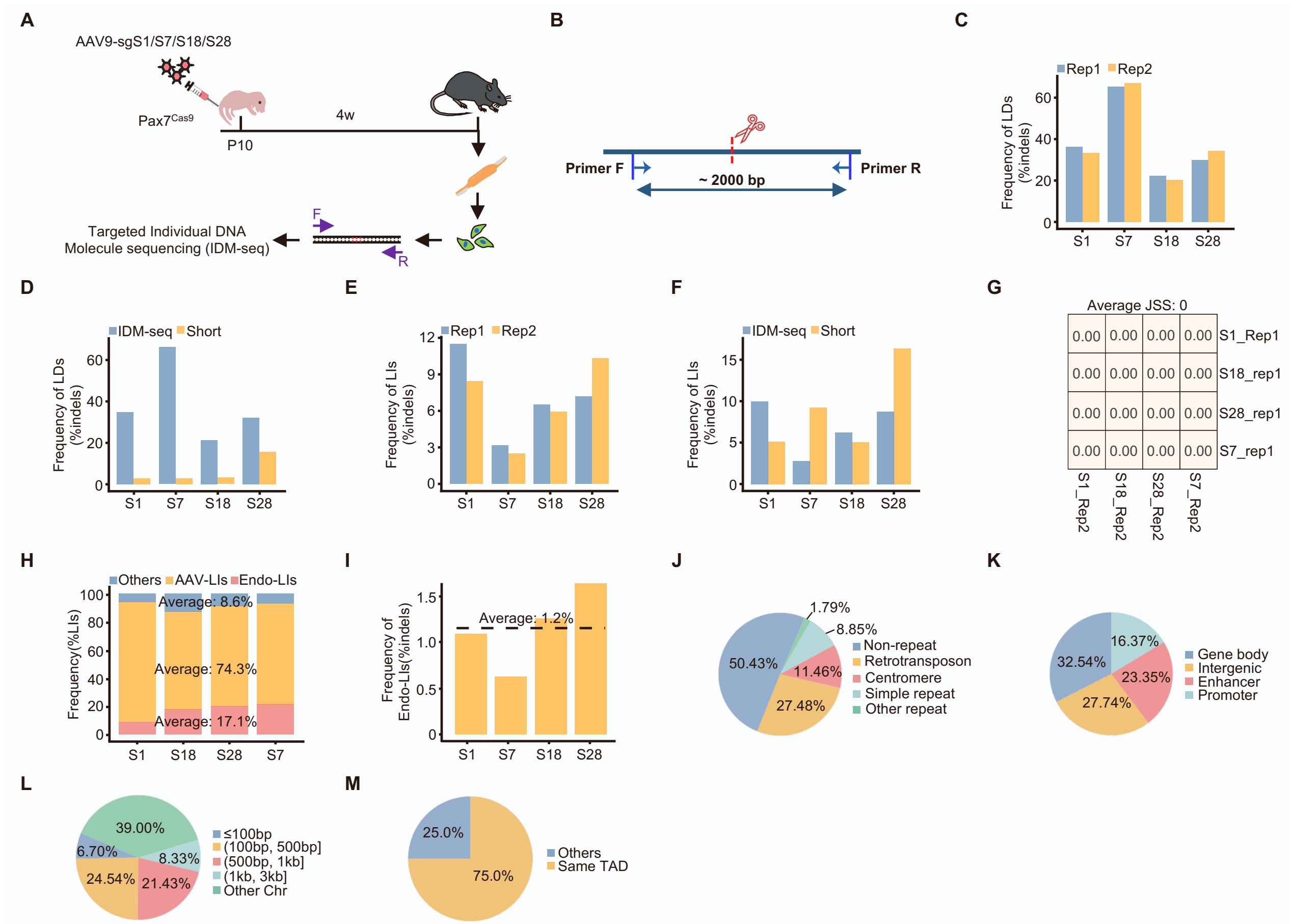

Figure S10. Detection of large on-target modifications by long-read sequencing. (**A**) Schematic showing experimental design for detecting large on-target modifications by IDM-seq at S1, S7, S18 and S28. 5×10^{11} vg of AAV9-dual sgRNA virus targeting each site was IM injected into Pax7^{Cas9} mouse at P10, followed by MuSC isolation four weeks after injection. Sequences encompassing each site were amplified and subjected to IDM-seq. (**B**) Schematic showing the amplification range for IDM-seq. An approximately 2,000 bp region flanking the Cas9 cut site was amplified for each site. (**C**) Frequency of ≥ 50 bp LDs for each site, calculated as percentage in total indels. (**D**) Comparison of the average LD frequency detected by IDM-seq vs. short-read NGS. (**E**) Frequency of ≥ 20 bp LIs for each site, calculated as percentage in total indels. (**F**) Comparison of the average LI frequency detected by IDM-seq vs. short-read NGS. (**G**) JSSs for LIs detected by IDM-seq. Each square shows the JSS between all LIs of corresponding targets. The average JSS of paired replicates across all targets is shown. (**H**) Frequency of different types of LIs.

The average frequency of each type across all sites is shown. (**I**) Frequency of ≥ 20 bp Endo-LIs for each site, calculated as percentage in total indels. The average frequency is shown by dash line. (**J**) Pie chart showing the frequency of donor sequence categorized by types of repetitive elements for Endo-LIs. (**K**) Pie chart showing the frequency of donor sequence categorized by genomic annotations for Endo-LIs. (**L**) Pie chart showing distribution of distances between insertion donors for Endo-LIs and associated DSBs. (**M**) Pie chart showing the frequency of donor sequences originating from the DSB-containing TADs or other loci on the same chromosome.

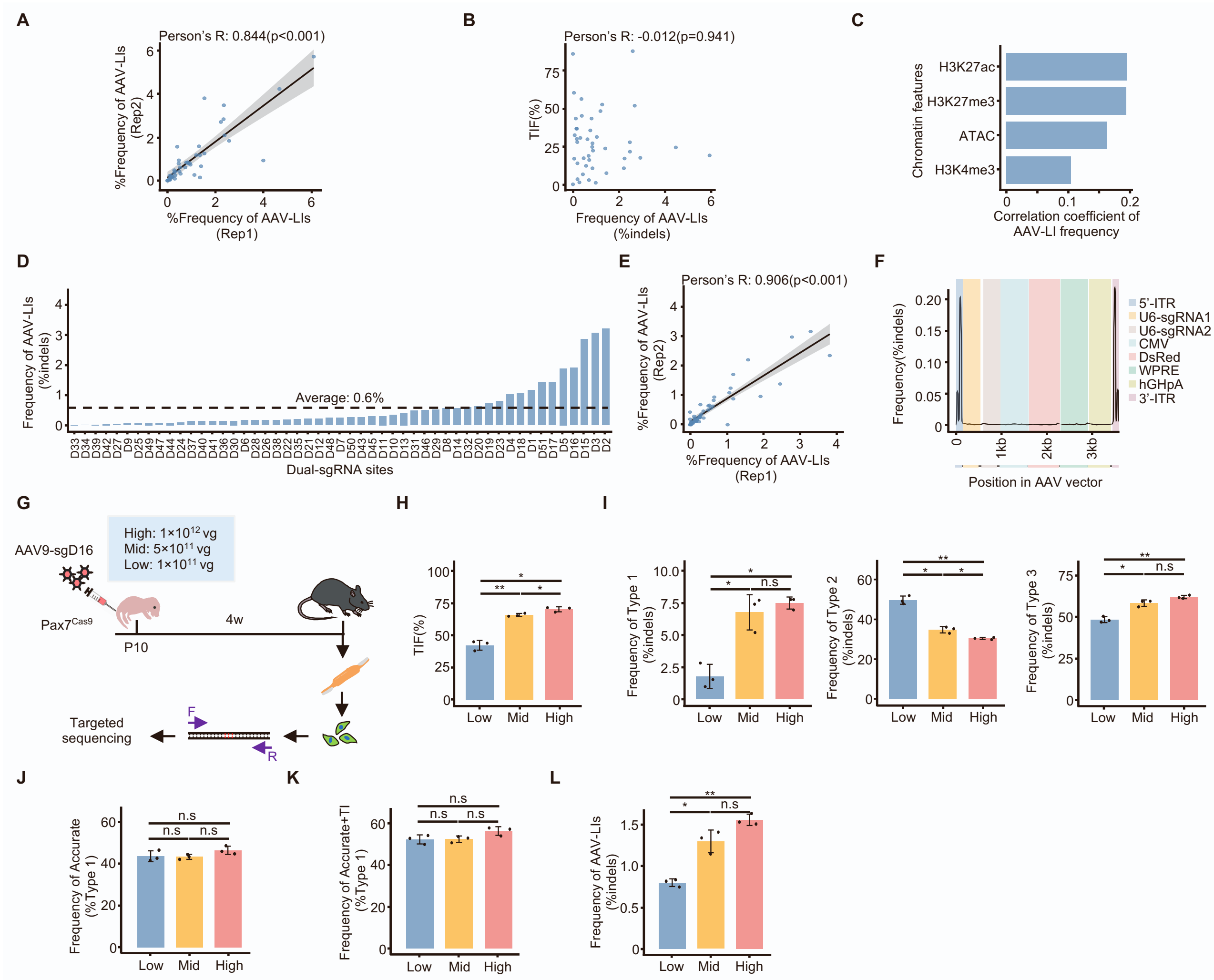

Figure S11. AAV integration is a general outcome of CRISPR/Cas9-induced editing in MuSCs *in vivo*. (**A**) Pearson's correlation of AAV-LI frequency in total indels between two biological replicates across single-sgRNA targets. (**B**) Pearson's correlation of TIF with AAV-LI frequency. (**C**) Spearman's correlation between AAV-LI frequency and the indicated chromatin features. (**D**) Frequency of AAV-LIs in dual-sgRNA group, calculated as percentage in total indels. The average frequency of AAV-LIs across all sites is shown by the dash line. (**E**) Pearson's correlation of AAV-LI frequency between two biological replicates across dual-sgRNA targets. (**F**) Average insertion frequency of indicated AAV-dual sgRNA vector regions. (**G**) Schematic showing experimental design for assessing the effect of AAV dosage on AAV-LI at the D16 site. High (1×10^{12} vg), Mid (5×10^{11} vg) or Low (1×10^{11} vg) dose of AAV9-dual sgRNA virus targeting the

D16 site were IM injected into $Pax7^{Cas9}$ mouse at P10 (n=3 mice), followed by MuSC isolation four weeks after injection. Sequences encompassing the D16 site were amplified and subjected to unbiased deep-sequencing and analysis. (**H**-**L**) Barplot illustrating TIF (**H**), frequency of Type 1 indels (**I**, Left), Type 2 indels (**I**, Middle), Type 3 indels (**I**, Right), Accurate events (**J**), Accurate + TI events (**K**) and AAV-LIs (**L**) for the three dosage groups (n=3 mice in each group). Statistical significance was calculated using a t-test for (**H**) - (**L**). *$p < 0.05$, **$p < 0.01$ and ns, no significance.

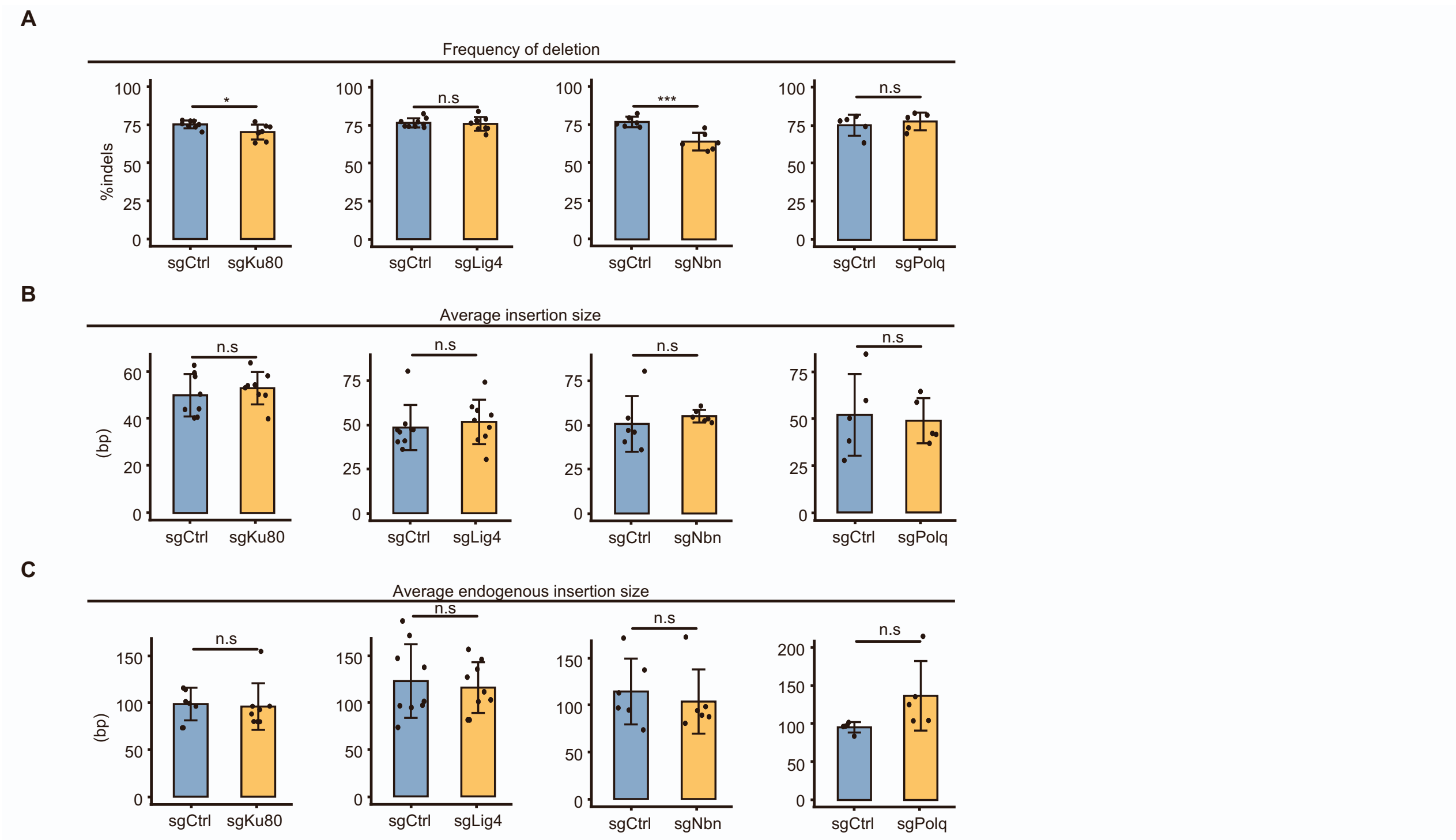

Figure S12. *In vivo* perturbation of NHEJ/MMEJ pathways can modulate CRISPR/Cas9 editing in MuSCs. (**A-C**) Frequency of deletions (**A**), average insertion size (**B**) and average endogenous insertion size (**C**) after *in vivo* depletion of KU80, LIG4, NBN or POLQ is shown. n=5 to 9 mice in each group. Statistical significance was calculated using a paired t-test for (**A**) - (**C**). *p < 0.05, ***p < 0.001 and ns, no significance.

Intended	Small deletion
	Small insertion
Un-intended	Large deletion
	Large insertion(Vector intergration; Endo-LI)
	Genomic rearrangement
	Chromosome truncation
	Copy-neutral loss of heterozygosity
	Whole chromosome loss
	Chromothripsis

Figure S13. Summary of main repair outcomes mediated by CRISPR/Cas9.

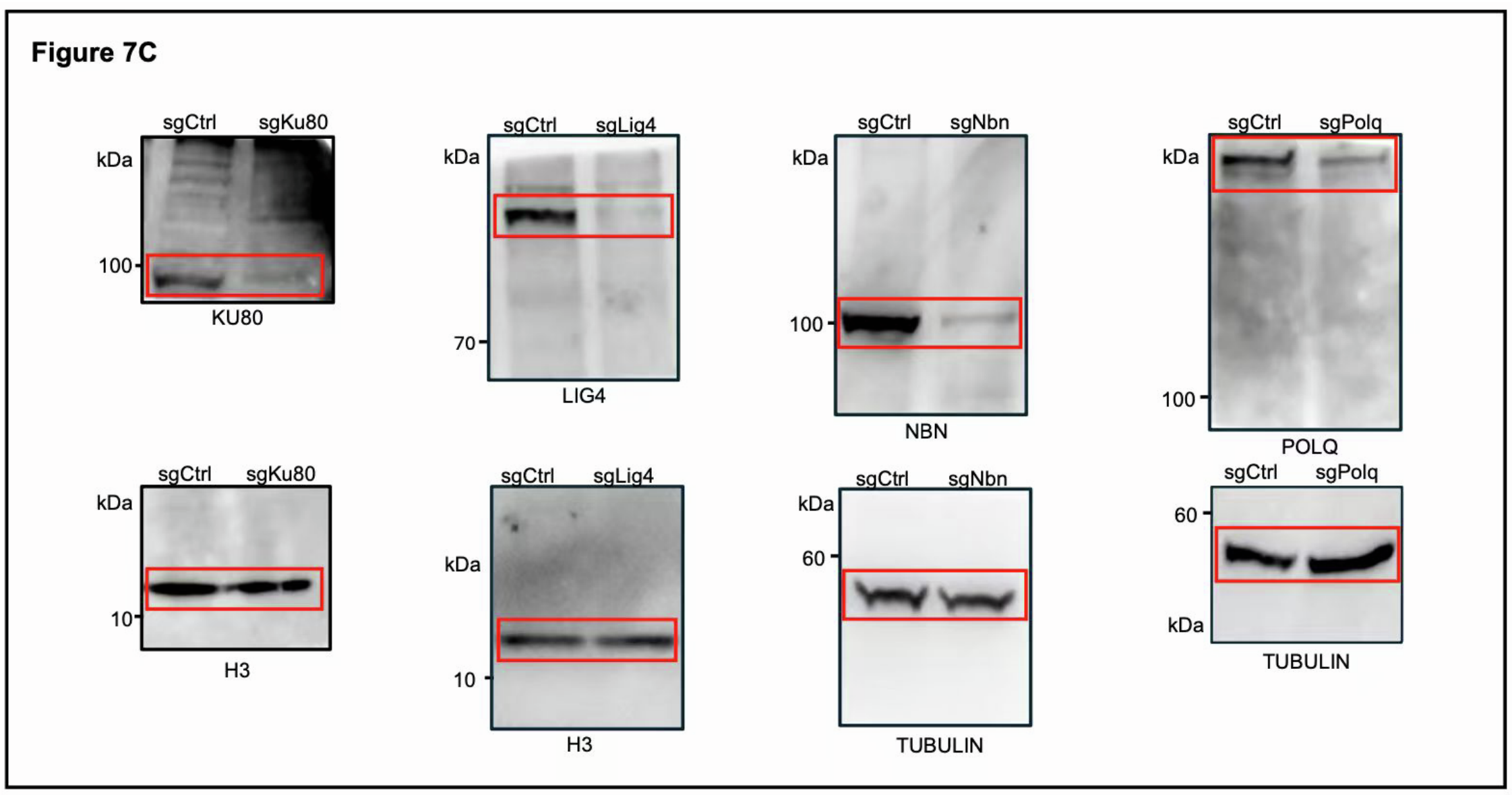

Figure S14. Uncropped Western blot images.
